# *N*-Terminal Cysteine Bioconjugation with (2-Cyanamidophenyl)boronic Acids Enables the Direct Formation of Benzodiazaborines on Peptides

**DOI:** 10.1021/acs.orglett.3c01835

**Published:** 2023-07-19

**Authors:** Rita Padanha, Rafaela A. N. Cavadas, Pedro Merino, João P. M. António, Pedro M. P. Gois

**Affiliations:** † Research Institute for Medicines (iMed.ULisboa), Faculty of Pharmacy, Universidade de Lisboa, Av. Prof. Gama Pinto, 1649-003 Lisboa, Portugal; ‡ Instituto de Biocomputación y Física de Sistemas Complejos (BIFI), Universidad de Zaragoza, 50009 Zaragoza, Spain

## Abstract

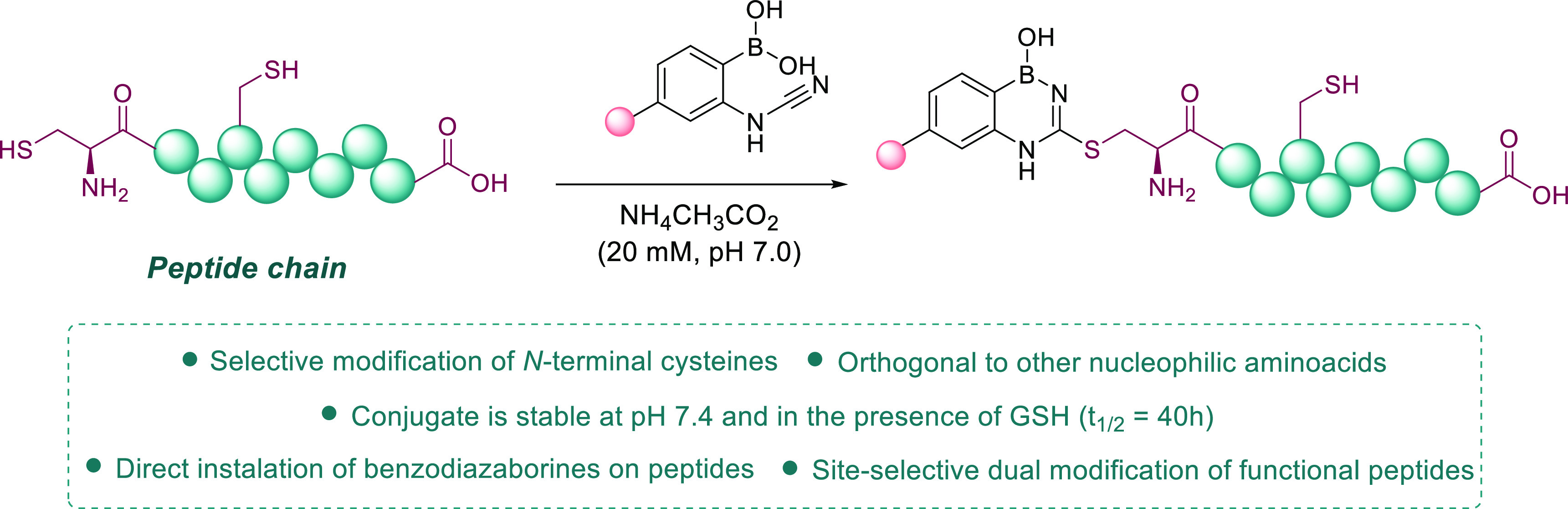

Benzodiazaborines (BDABs) have emerged as a valuable tool to produce stable and functional bioconjugates via a click-type transformation. However, the current available methods to install them on peptides lack bioorthogonality, limiting their applications. Here, we report a strategy to install BDABs directly on peptide chains using (2-cyanamidophenyl)boronic acids (2CyPBAs). The resulting BDAB is stabilized through the formation of a key intramolecular B–N bond. This technology was applied in the selective modification of *N*-terminal cysteine-containing functional peptides.

In recent years, boron compounds evolved from useful synthetic reagents to central actors of different fields.^1^ In chemical biology, the dynamic coordination profile of boron has been extensively explored to prepare biologically active compounds, to functionalize biomolecules, and to assemble bioconjugates with specific mechanisms to respond to different stimuli.^2−5^ Among the different families of boron-containing molecules, benzodiazaborines (BDABs) are a particularly noteworthy class of boronic acid derivatives because they elicit an array of biological properties and exhibit a naphthoid isoster structure that is now emerging as a powerful construction tool of functional bioconjugates.^6^

Dependent upon the substituent periphery, BDABs can exhibit very high stability in physiological conditions,^7^ responsiveness to reactive oxygen species,^8^ and a click-type synthesis using hydrazines and 2-carbonyl phenyl boronic acids.^9−11^ Despite these very positive features, the installation of BDABs on peptide chains requires the initial bioconjugation of hydrazine or 2-carbonyl phenyl boronic acid onto the peptide, followed by the addition of the second component. However, this reaction is poorly orthogonal because 2-carbonyl phenyl boronic acids are well-known to react with ε-amino groups of lysine^12,13^ and *N*-terminal residues, like cysteine,^14,15^ which can limit the post-functionalization with hydrazines. Therefore, the discovery of a bioconjugation reagent that could generate BDABs directly on the peptide chain, using natural amino acids as building blocks, would give direct access to this valuable functional core.

Cyanamides are electrophilic warheads that have been used in various inhibitors of cysteine proteases.^16,17^ However, this function has been mostly overlooked in the development of bioconjugation reagents targeting solvent-exposed cysteine residues. A likely explanation for this lack of use is the fact that, upon reaction with Cys, cyanamide generates a thiourea-like function that in dilute aqueous conditions rapidly hydrolyzes to form urea. Considering this structure, we envisioned that the incorporation of cyanamide in the *ortho* position of a phenyl boronic acid (2CyPBA) would generate a cysteine alkylation reagent that directly generates the BDAB core through the formation of an intramolecular B–N bond (Figure 1).

**Figure 1:**
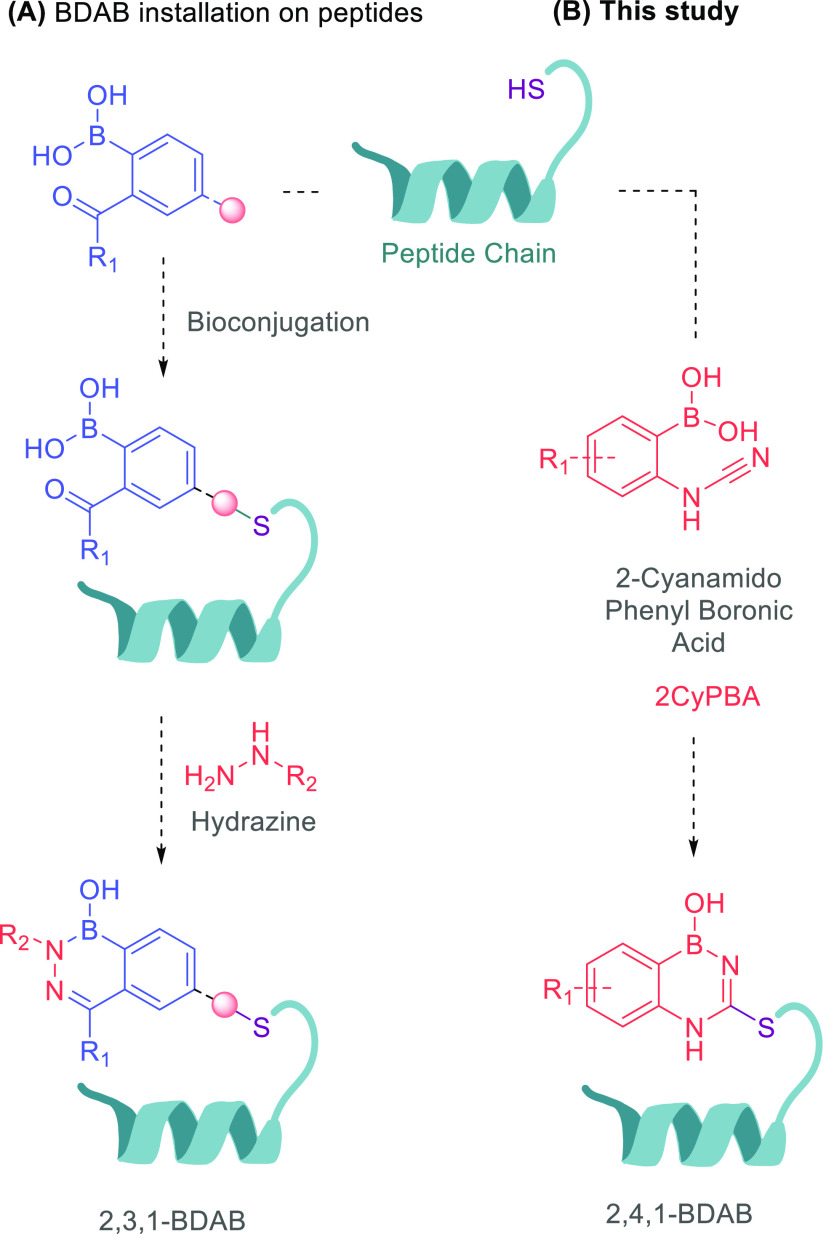
Proposed strategy to install BDABs on peptide chains using (2-cyanamidophenyl)boronic acid (2CyPBA).

To test this idea, we prepared 2CyPBA **1** and tested it in the reaction with 1.0 equiv of cysteine in NH_4_CH_3_CO_2_ (20 mM) at pH 7.0, and the reaction was monitored by electrospray ionization mass spectrometry (ESI–MS). After 24 h, proposed BDAB **2** was the major product observed (Figure 2B). Compound **2** was then isolated by semi-preparative high-performance liquid chromatography (HPLC) in 75% yield and fully characterized by nuclear magnetic resonance (NMR) and high-resolution mass spectrometry (HRMS). Differently, thiourea **4** is not stable under these conditions, which clearly showcases the importance of the proximal boronic acid function to favor the bioconjugation reaction (Figure 4A).

**Figure 2:**
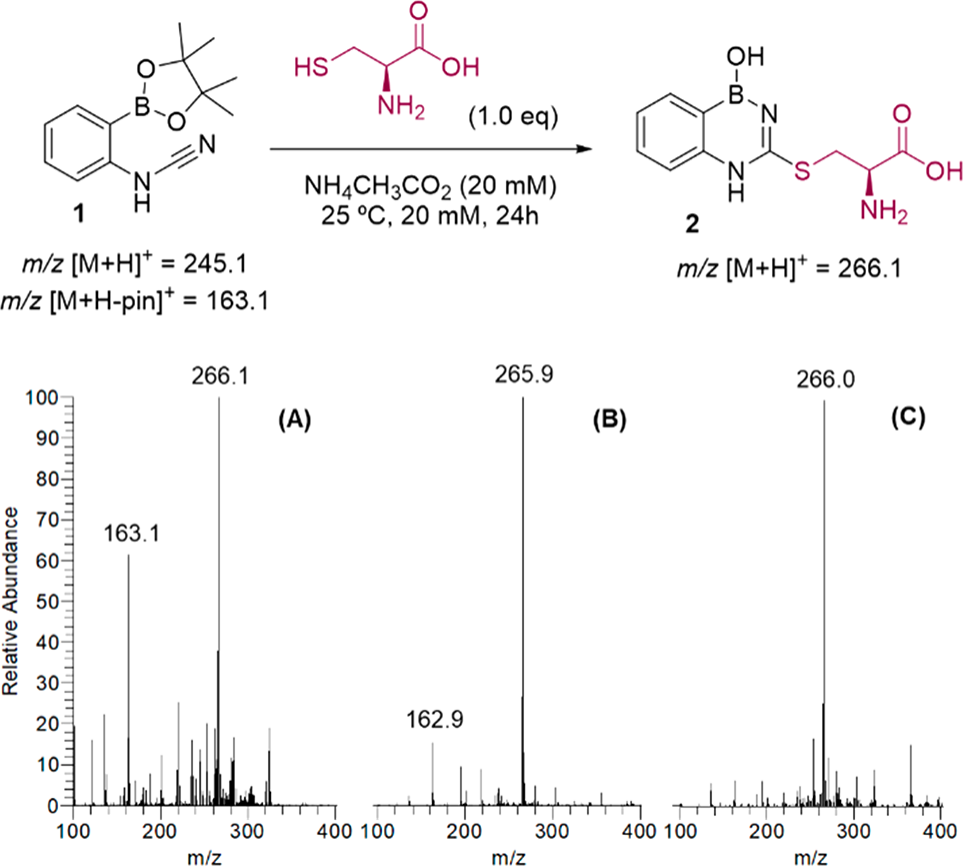
Reaction of cyanamide **1** with cysteine (1.0 equiv) in NH_4_CH_3_CO_2_ (20 mM), 25 °C, 20 mM, and 24 h at pH (A) 5.0, (B) 7.0, and (C) 8.0.

After our initial hypothesis was confirmed, we proceeded to optimize the reaction conditions. As shown in Figure 2, BDAB **2** was readily formed in a pH range of 5–8, with a preference for neutral and slightly basic pH values. Similarly, the reaction is favored at higher concentrations, with 20 mM achieving cleaner reaction profiles than 2 and 10 mM (section 4 of the Supporting Information).

Then, to evaluate the kinetics of this reaction, we prepared a 50 mM solution of cyanamide **1** in ND_4_CD_3_CO_2_ solution (20 mM) at pH 7.0 and added 10 equiv of cysteine (Figure 3A). The reaction was monitored by ^1^H NMR over 26 h, at 2 h intervals, and a clean reaction was observed, with no visible intermediate species/side products being formed (section 5 of the Supporting Information). From this experiment, a pseudo-first-order *k*_obs_ of 3.44 × 10^–5^ s^–1^ was calculated (Figure 3B). Moreover, BDAB **2** displayed adequate stability in physiological conditions with degradation half-lives of approximately 40 h in both phosphate-buffered saline (PBS) at pH 7.4 (Figure 3C) and the presence of 10 equiv of glutathione (section 6 of the Supporting Information).

**Figure 3:**
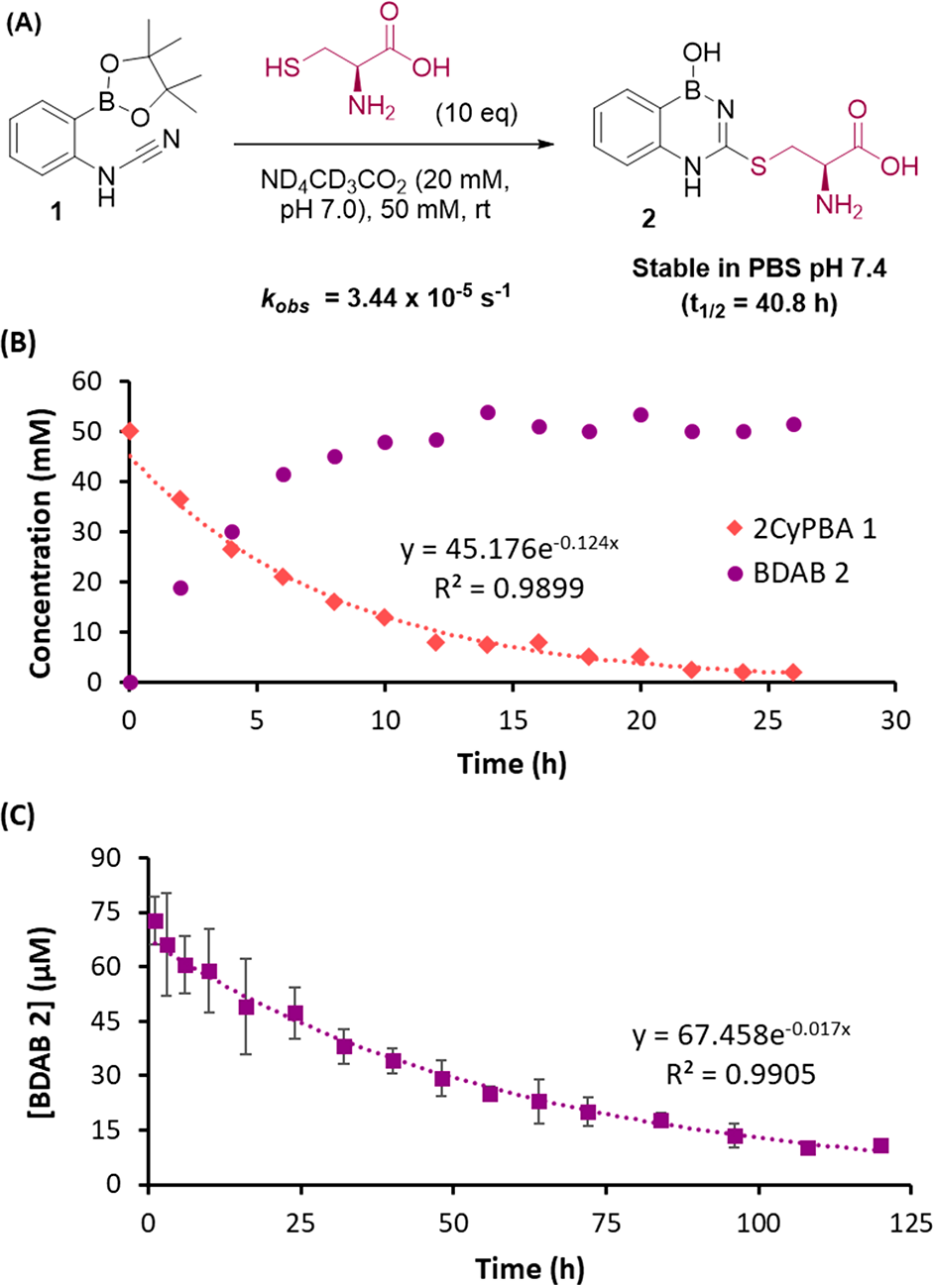
(A) Cyanamide **1** reacts with cysteine (10 equiv, 50 mM) in ND_4_CD_3_CO_2_ (20 mM, pH 7.0) at room temperature (rt) to generate BDAB **2**. (B) Reaction of compound **1** with cysteine (10 equiv) in ND_4_CD_3_CO_2_ (20 mM, pH 7.0) was followed by ^1^H NMR. (C) Stability of BDAB **2** (10 mM) was evaluated by HRMS in PBS at pH 7.4.

Next, we set out to expand the scope of the reaction and to understand its mechanism. As previously mentioned, boronic acid plays an important role in the reaction because, without it, the product formed is unstable and quickly hydrolyzed (Figure 4A). Likewise, the reaction of compound **2** with acetylcysteine appears to block 2CyPBA reactivity, because BDAB **5** was not detected by ESI–MS (Figure 4B). This result is very promising, because it unlocks the possibility of using this technology to selectively modify *N*-terminal cysteines in the presence of in-chain cysteines. Moreover, the presence of a methyl group in cyanamide (compound **6**) led to a sluggish reaction with a low apparent conversion after 24 h when compared to the reaction with cysteine (Figure 4C). Finally, the introduction of an ester group in position 4 was well-tolerated and may act as a derivatization handle for future applications of this technology (Figure 4D and section 7 of the Supporting Information).

**Figure 4:**
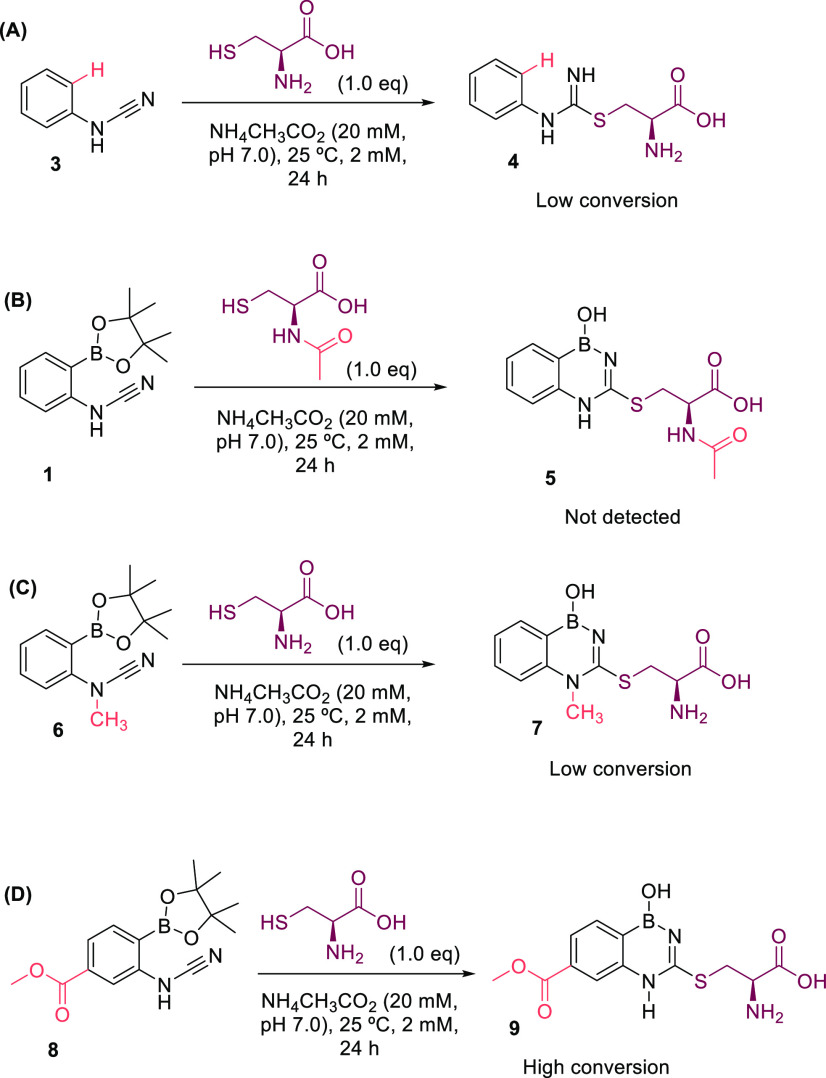
Scope and mechanistic studies by low-resolution mass spectrometry (LRMS). (A) Absence of proximal boronic acid renders thiourea **4** unstable and detected in low concentrations. (B) Compound **5** is not detected as a result of the lack of reactivity between *N*-acetyl cysteine and cyanamide **1**. (C) Methylation of cyanamide **6** hinders its reactivity, and compound **7** is detected with low conversion. (D) Introduction of an ester group in the *meta* position of cyanamide does not interfere with the formation of BDAB **9**.

Considering these results, we performed a detailed mechanistic study using density functional theory (DFT) calculations to rationalize the experimental findings. Therefore, we studied diazaborine formation with cyanamides **1** and **6**. For the *N*-methyl derivative **6**, the barrier is higher (28.5 kcal/mol versus 24.4 kcal/mol for compound **1**) as a result of steric hindrance exerted by the methyl group to the S attack, which closes the entry path (Figure 5A). On the other hand, two possible orientations of the *N*-cyano group were considered for 2CyPBA **1** (section 14 of the Supporting Information). As illustrated in Figure 5B, the most stable orientation model presents an additional interaction between the NH group and oxygen of boron ester. Furthermore, the reaction takes place through a hydrogen transfer from the thiol group mediated by the free amino group of cysteine, which should be basic enough to accept the proton and promote the hydrogen transfer to the cyano group. The observed α-amine effect may justifiy the absence of reactivity observed with *N*-acetyl cysteine, because the acetamido group is not protonated at pH 7 and is unable to undergo the initial hydrogen transfer step. After proton transfer and nucleophilic S attack, hydrolysis of the boronate group provides the final diazaborine.

**Figure 5:**
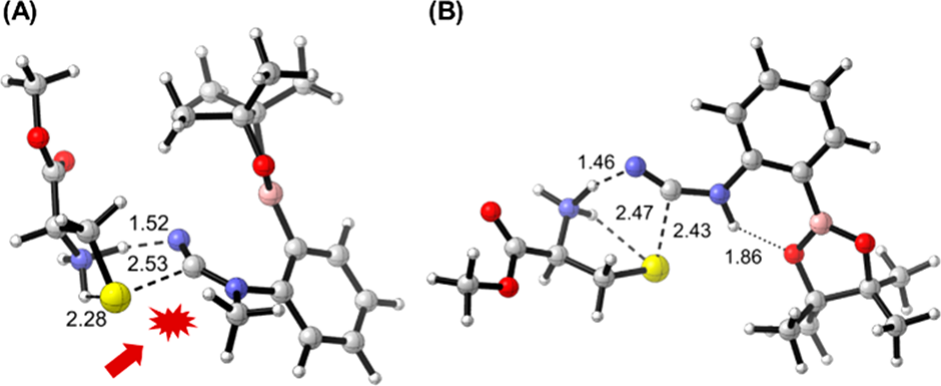
Transition structures corresponding to BDAB formation using cysteine and (A) cyanamide **6** and (B) cyanamide **1**.

After establishing the reaction mechanism, we began evaluating its performance in more complex systems. Various reports indicate that the neighboring amino acids may have an important effect in bioconjugation efficiency.^18,19^ As such, we prepared a small library of cysteine-containing dipeptides featuring hydrophobic, polar uncharged, and charged amino acids and evaluated their bioconjugation with boronated cyanamide **1**. The reaction proceeds with moderate to high conversion rates with most dipeptides tested, with the exception of Cys-Glu, where only 51% conversion is observed (Figure 6). Moreover, no cross-reactivity was observed with other nucleophilic amino acids, such as lysine, histidine, serine, threonine, and tyrosine, which confirmed the proposed selectivity for cysteine (section 8 of the Supporting Information).

**Figure 6:**
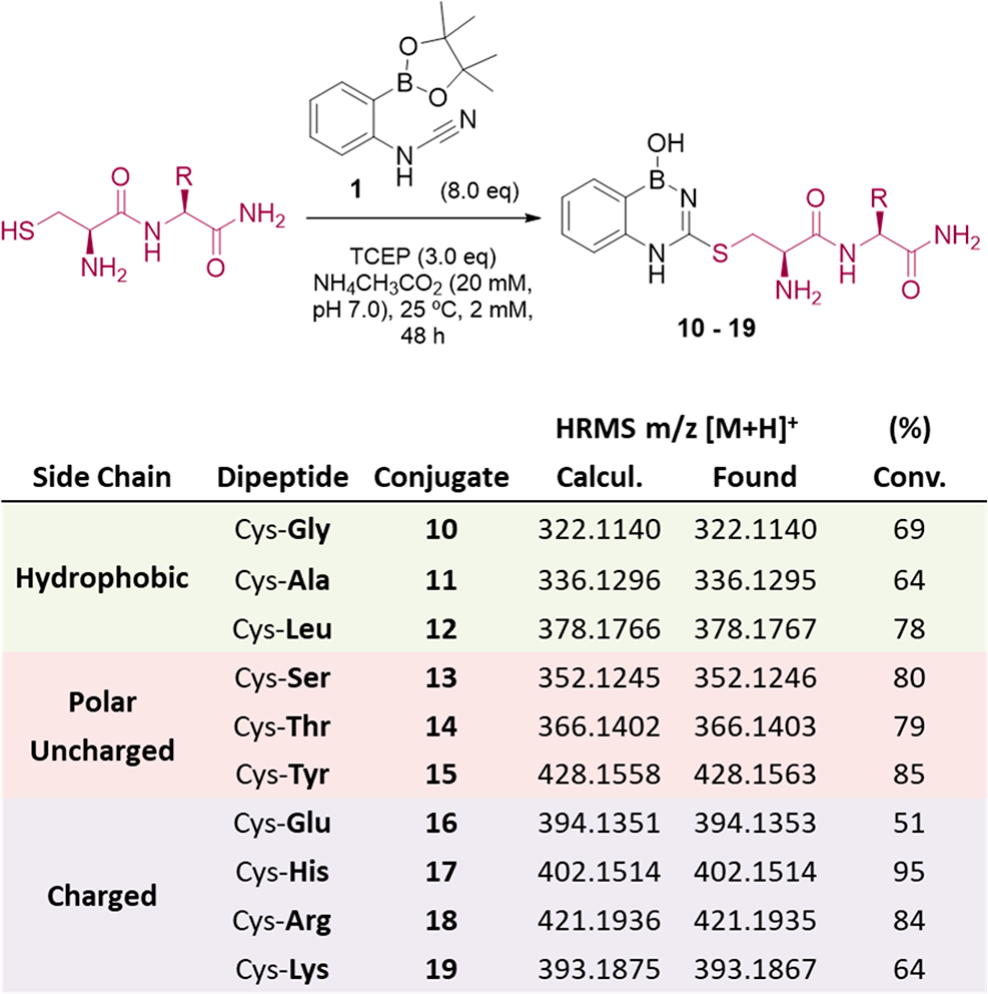
Effect of the neighboring amino acid on the conjugation efficiency with 2CyPBA **1**.

Finally, we advanced to the modification of more complex peptides. Cys-bombesin and *C*-ovalbumin, both featuring *N*-terminal cysteines, were incubated for 24 h with 10 equiv of 2CyPBA **1** at pH 7.0, and high conversions (99 and 76%, respectively) were observed with both peptides. However, when using the GV-1001 peptide, which displays a *C*-terminal cysteine, no conjugation is observed after 24 h of incubation with cyanamide **1** (Figure 7 and sections 9, 10, and 12 of the Supporting Information). These results validate the application of this technology for the modification of *N*-terminal cysteines.

**Figure 7:**
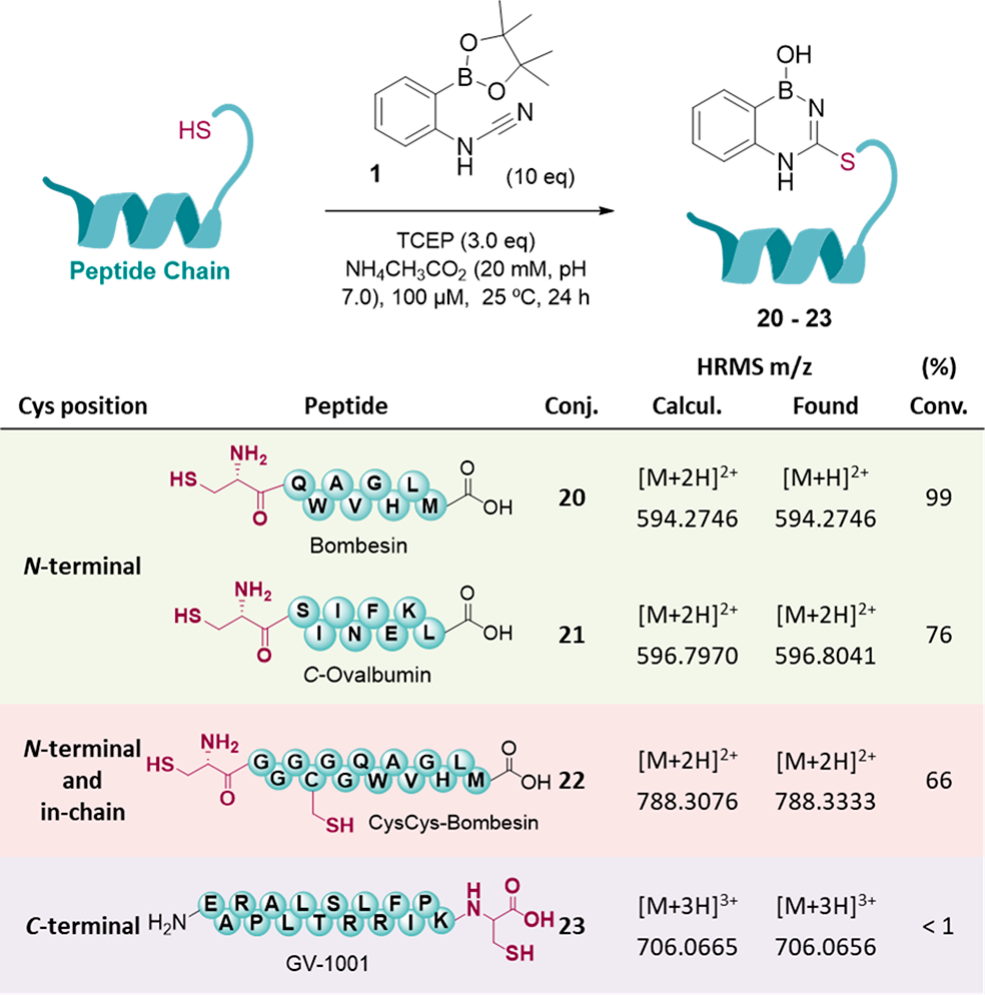
Reaction of cyanamide **1** with peptides featuring cysteine in different positions (*N*-terminal, in-chain, and both).

Then, we investigated the possibility of selectively modifying *N*-terminal cysteine in the presence of other cysteines. For this, we designed a modified bombesin that displays both *N*-terminal and in-chain cysteine (CysCys-bombesin). Upon incubation with 10 equiv of compound **1** for 24 h at pH 7.0, the major product observed corresponded to the single-modified conjugate with 67% conversion (section 11 of the Supporting Information).

After confirming the selective modification of the *N*-terminal cysteine, we envisioned the construction of a dual-modified functional conjugate. First, we accomplished the selective modification of CysCys-bombesin with cyanamide **8**, to obtain conjugate **24**. Then, the addition of 10 equiv of maleimide **25** promoted the alkylation of the in-chain cysteine, generating the desired dual-modified conjugate **26** (Figure 8 and section 13 of the Supporting Information).

**Figure 8:**
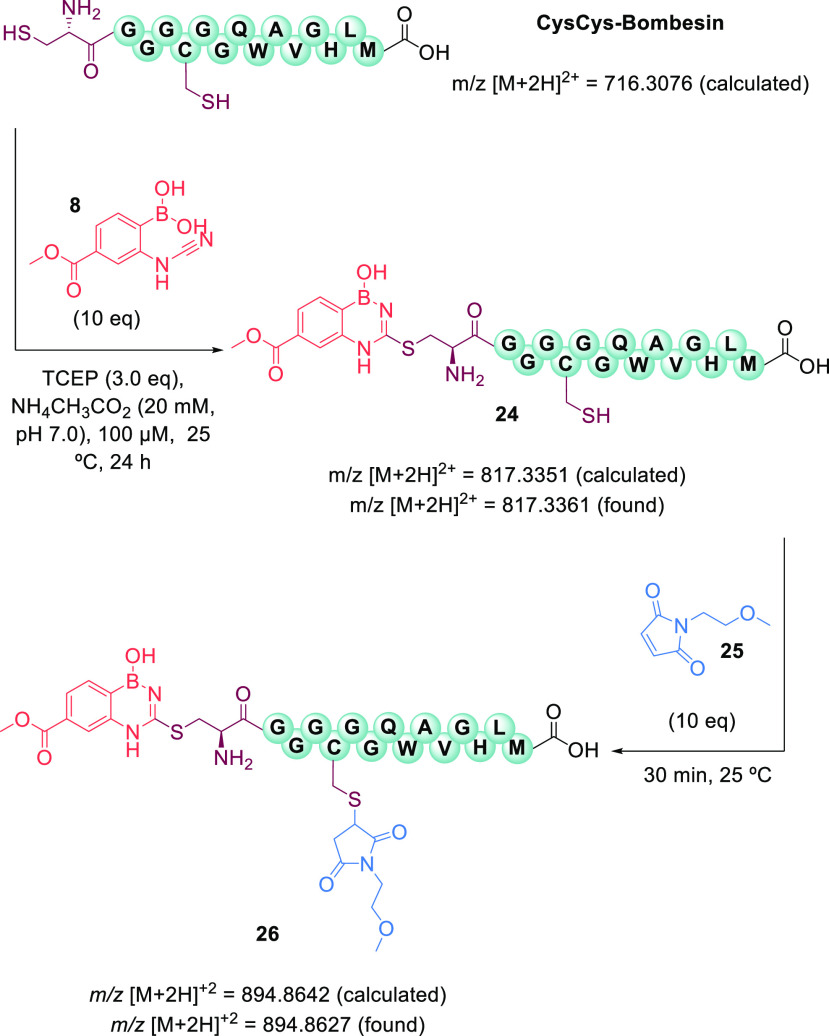
Sequential site-selective modification of CysCys-bombesin with boronated cyanamide **8** and maleimide **25** to obtain dual-modified conjugate **26**.

In summary, in this work, we developed a novel methodology that enables the direct installation of BDABs in peptides. This methodology is grounded on the role of proximal boronic acid in stabilizing the addition of a cysteine to cyanamides. We demonstrated the BDAB **2** formation in ammonium acetate solution preferably at neutral or slightly basic pH values and at higher reaction concentrations as well its stability under physiological conditions. A detailed DFT study was performed to elucidate the mechanism of BDAB formation, which revealed the importance of the B–N bond formation in the stabilization of the conjugate and the role of the free amine group of *N*-terminal cysteines for the conjugation success. This methodology is compatible with different amino acid side chains, resulting in dipeptide conjugates with moderate to high conversion rates. Particularly, these cysteine alkylation reagents are selective for *N*-terminal cysteines, showing reactivity toward Cys-bombesin and *C*-ovalbumin but not toward GV-1001. With this strategy, we obtained a dual modification of CysCys-bombesin with 2CyPBA **8** and maleimide **25** as derivatization handles. As future work, we intend to apply this strategy to protein modification. Although natural proteins containing a *N*-terminal cysteine are rare, several methods for the production of proteins with *N*-terminal cysteines are available, empowering the practical use of this site-specific bioconjugation strategy.

## Data Availability

The data underlying this study are available in the published article and its Supporting Information.

## Supporting Information Available

Experimental details, NMR spectra, ESI–MS spectra, and other materials (PDF)
