# Supplementary material for: N-Terminal Cysteine Bioconjugation with (2-Cyanamidophenyl)boronic Acids Enables the Direct Formation of Benzodiazaborines on Peptides
Source: Org Lett. 2023 Jul 19;25(29):5476–80. doi: 10.1021/acs.orglett.3c01835 (PMC10391619; doi:10.1021/acs.orglett.3c01835)
Supplement: Supplementary file 1 — ol3c01835_si_001.pdf [file ol3c01835_si_001.pdf]

Supplementary Information for

N-terminal Cysteine Bioconjugation with (2-Cyanamidophenyl)boronic Acids Enables the Direct Formation of Benzodiazaborines on Peptides

Rita Padanha[a], Rafaela A. N. Cavadas[a], João P. M. António[a], Pedro Merino[b] and Pedro M. P. Gois*[a]

[a]Research Institute for Medicines (iMed.ULisboa), Faculty of Pharmacy, Universidade de Lisboa, Av. Prof. Gama Pinto, 1649-003, Lisboa, Portugal

[b]Instituto de Biocomputación y Física de Sistemas Complejos (BIFI), Universidad de Zaragoza, 50009 Zaragoza, Spain.

Table of Contents

1. General Remarks 3
2. Synthesis of Cyanamides 4
    2.1. Cyanamides **1** and **3** 4
    2.2. Cyanamide **6** 5
    2.3. Cyanamide **8** 6
3. Synthesis of Benzodiazaborine **2** 11
4. Optimization of Benzodiazaborine **2** Formation 12
    4.1 pH Study 12
    4.2. Reaction Concentration Study 13
5. Kinetic Studies of Benzodiazaborine **2** Formation 15
6. Benzodiazaborine **2** Stability Studies 17
    6.1. Stability in PBS pH 7.4 17

6.2. Stability in Presence of Glutathione 18
7. ESI-MS Mechanistic Studies 20
7.1. ESI-MS Assays with Cyanamide **1** and *N*-Acetyl Cysteine 20
7.2. ESI-MS assays with Cyanamide **3** and *L*-Cysteine 21
7.3. ESI-MS Assays with Methylated Cyanamide **6** and *L*-Cysteine 23
7.4. ESI-MS Assays with Cyanamide **8** and *L*-Cysteine 24
8. General Procedure for the ESI-MS Assays with Cys Dipeptides and Cyanamide **1** 25
8.1. Cys-Gly 25
8.2. Cys-Ala 27
8.3. Cys-Leu 29
8.4. Cys-Ser 31
8.5. Cys-Thr 33
8.6. Cys-Tyr 35
8.7. Cys-Glu 37
8.8. Cys-His 39
8.9. Cys-Arg 41
8.10. Cys-Lys 43
9. ESI-HRMS Assays with Cys-Bombesin and Cyanamide **1** 45
10. ESI-HRMS Assays with *C*-Ovalbumin and Cyanamide **1** 47
11. ESI-HRMS Assays with CysCys-Bombesin and Cyanamide **1** 49
12. ESI-HRMS Assays with GV-1001 and Cyanamide **1** 51
13. ESI-MS Assays with CysCys-Bombesin and Cyanamide **8** 53
14. Computational Studies 57
15. References 82
16. NMR spectra 85

1. General Remarks

All reagents and solvents used were purchased from Fluorochem, Alfa Aesar, TCI or Sigma-Aldrich without further purifications. The solvents used in ESI-MS and LC-MS experiments were of spectroscopic quality. In case of air-sensitive reactions, solvents and triethylamine were obtained in anhydrous conditions by distillation under nitrogen. Particularly, anhydrous dichloromethane and tetrahydrofuran were obtained using a Pure Solv™ Micro 100 Liter solvent purification system with activated alumina column. Dipeptides were prepared by Ismael Compañón and Dr. Francisco Corzana from Departamento de Química, Centro de Investigación en Síntesis Química, Universidad de La Rioja. Cys-Bombesin, C-Ovalbumin, CysCys-Bombesin and GV-1001 peptides were purchased from GeneCust. Maleimide **25** was prepared according to a reported procedure.[1] All chemical procedures were performed in air at ambient temperature (~22 ºC) and pressure (1.0 atm) unless indicated otherwise. Reaction mixtures were analyzed by thin layer chromatography using Merck silica gel $60F_{254}$ aluminium plates and visualized by exposure to UV light or by dipping the plates in *p*-anisaldehyde or phosphomolybdic acid stains followed by heating. Column chromatography was performed with silica gel Geduran® Si 60 (0.040-0.063 mm) purchased from Merk.

NMR spectra were recorded in a Bruker Fourier 300 and 400 (Bruker, Massachusetts, USA) using $CDCl_3$ and D_2O as deuterated solvents. The NMR spectrometers are part of the National NMR Network (PTNMR) and are partially supported by Infrastructure Project Nº 022161c and ROTEIRO/0031/2013– PINFRA/22161/2016 (cofinanced by FEDER through COMPETE 2020, POCI and PORL and FCT through PIDDAC). All coupling constants (*J* values) are expressed in Hertz (Hz) and chemical shifts (δ) in parts per million (ppm). Multiplicities are given as: s (singlet), br (broad) d (doublet), dd (double doublet), dt (double triplet), t (triplet), tt (triple triplet), q (quartet), quint (quintuplet) and m (multiplet).

Low resolution mass spectra were recorded in a LCQ Fleet Ion Trap Mass Spectrometer (Thermo Fisher Scientific, Germany) equipped with an electrospray interface. High resolution mass spectra were carried on in a Thermo Scientific Q Exactive hybrid quadrupole-Orbitrap mass spectrometer (Thermo ScientificTM Q ExactiveTM Plus). The Liquid chromatography–mass spectrometry (LC-MS) runs were performed using a Dionex Ultimate 3000 UHPLC+ system equipped with a Multiple-Wavelength detector and a imChem Surf C18 TriF 100 Å 3 µm 100 x 2,1 mm column connected to Thermo Scientific Q Exactive hybrid quadrupole-Orbitrap mass spectrometer (Thermo ScientificTM Q ExactiveTM Plus). Semi-preparative RP HPLC was performed on a Dionex Ultimate 3000 system using a Phenomenex Luna® 10 µm S6 C18(2) 100 Å, LC Column 250 x 10 mm.

2. Synthesis of Cyanamides

2.1. Cyanamides **1** and **3**

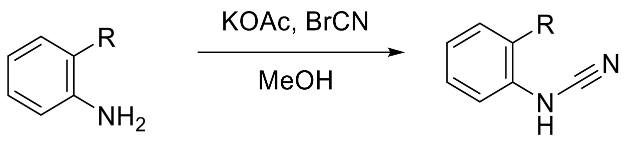

1: R = Bpin
3: R = H

The procedure used was adapted from one previously reported.[2] In a round bottom flask, aniline (1.1 mmol) and potassium acetate (1.7 mmol) were dissolved in methanol (7.0 mL). Cyanogen bromine (1.2 mmol) was added to the mixture at 0 °C and the reaction was stirred at room temperature for 16 h. Then, the solvent was evaporated and the crude was purified by column chromatography (0-10 % MeOH in CH_2Cl_2) to afford the title compounds. Spectral data of cyanamide **3** was in accordance with previous reports.[3]

Caution! Cyanogen bromine (BrCN) is a very toxic reagent. Thus, all of these operations should be conducted in an efficient fumehood.

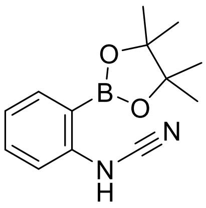

1

***N*-(2-(4,4,5,5-tetramethyl-1,3,2-dioxaborolan-2-yl)phenyl)cyanamide 1:** brown solid, 160 mg, 60 % yield. **^{1}H NMR** (300 MHz, $CDCl_3$): δ 8.09 (br s, 1H), 7.73 (dd, *J* = 7.5, 1.7 Hz, 1H), 7.52 – 7.44 (m, 1H), 7.27 – 7.19 (m, 1H), 7.04 (td, *J* = 7.4, 1.0 Hz, 1H), 1.36 (s, 12H). **^{13}C NMR** (75 MHz, $CDCl_3$): δ 144.1, 136.7, 133.5, 122.4, 114.3, 111.1, 84.8, 24.9. **HRMS:** *m/z* $[M+H]^+$ calculated for $C_{13}H_{18}BN_2O_2$ = 245.1456; found = 245.1459.

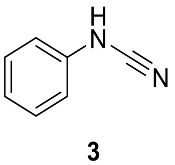

3

Phenylcyanamide 3: orange solid, 40 mg, 14 % yield. **^{1}H NMR** (300 MHz, $CDCl_3$): δ 7.29 - 7.21 (m, 2H), 7.01 (m, 1H), 6.92 (m, 2H), 5.70 (br s, 1H).

2.2. Cyanamide **6**

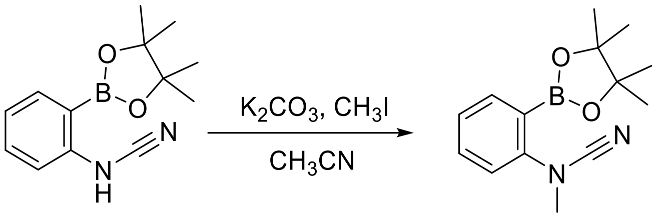

1 **6**

The procedure used was adapted from one previously reported.[4] In a round bottom flask, cyanamide **1** (100 mg, 0.410 mmol) and potassium carbonate (226 mg, 1.639 mmol) were suspended in dry acetonitrile (2,41 mL) under argon atmosphere. Then, iodomethane (255 µl, 4.10 mmol) was added and the reaction mixture was brought to 35 °C in an oil bath, for 3 h. The reaction was monitored by thin layer chromatography and, after conversion was complete, the mixture was filtered and the filtrate was evaporated to afford the title compound.

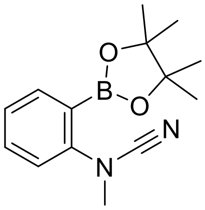

6

***N*-methyl-*N*-(2-(4,4,5,5-tetramethyl-1,3,2-dioxaborolan-2-yl)phenyl)cyanamide 6:** brown solid, 55 mg, 52 % yield. **^{1}H NMR** (300 MHz, $CDCl_3$): δ 7.71 (dd, *J* = 7.4, 1.8 Hz, 1H), 7.46 – 7.32 (m, 1H), 7.22 – 7.13 (m, 2H), 3.24 (s, 3H), 1.30 (s, 12H). **^{13}C NMR** (75 MHz, $CDCl_3$): δ 146.7, 138.0, 136.2, 133.3 (d, *J* = 6.1 Hz), 131.3, 127.3 (d, *J* = 6.0 Hz), 125.2, 123.7 (d, *J* = 6.3 Hz), 121.7, 117.2, 84.2, 42.6 (d, *J* = 11.5 Hz), 41.0, 29.9 – 21.5 (m). **HRMS:** *m/z* $[M+H]^+$ Calculated for $C_{14}H_{19}BN_2O_2$ = 257.1456; found = 257.1462.

2.3. Cyanamide **8**

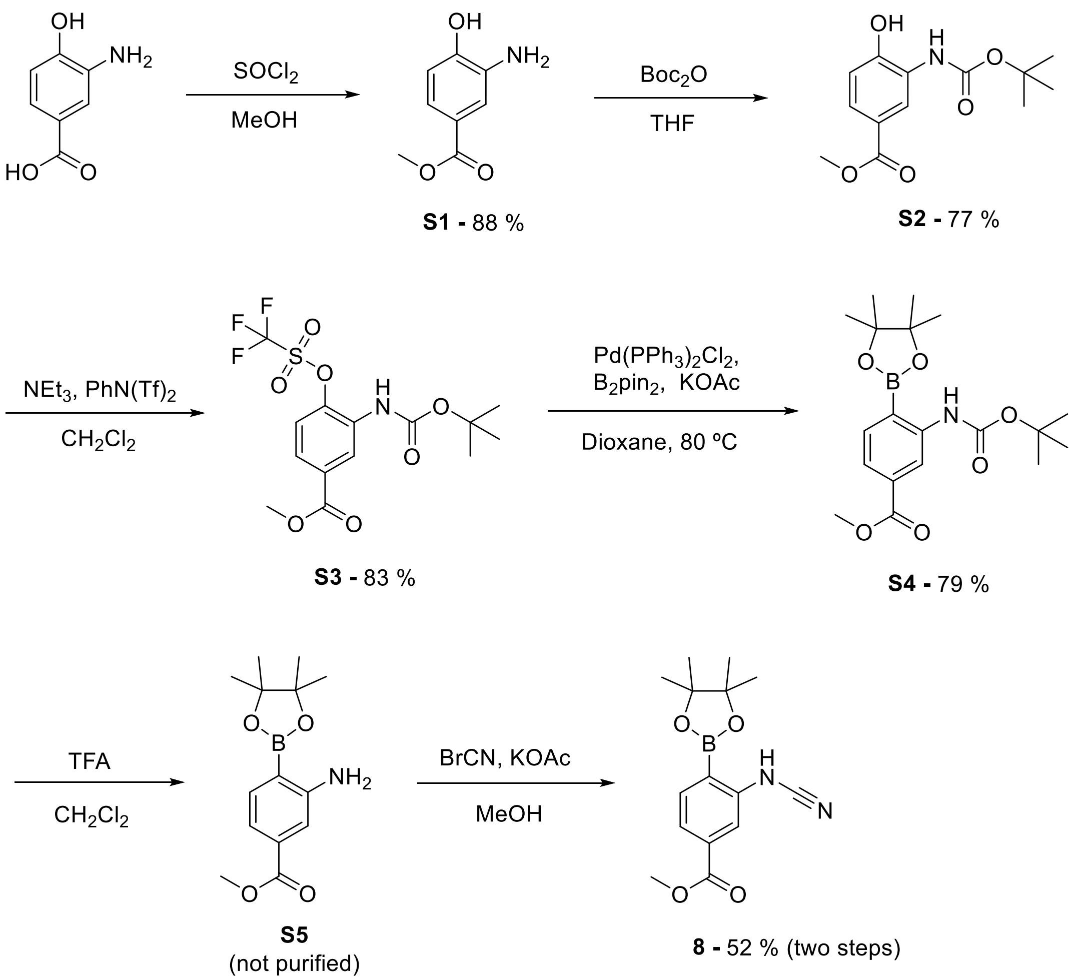

Figure S1 – General scheme for the synthesis of compound **8.**

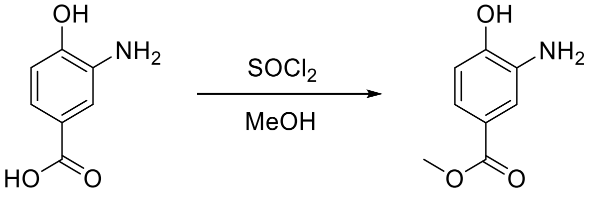

S1

The procedure used was adapted from one previously reported.[5] In a flame-dried round-bottomed flask, 3-amino-4-hydroxybenzoic acid (4.0 g, 26.1 mmol) was dissolved in dry methanol (80 mL). Then, thionyl chloride (4.77 mL, 65.3 mmol) was added dropwise at 0 °C, under argon atmosphere. The mixture was left under reflux overnight, in an oil bath. After cooling to room temperature, the reaction was neutralized by

addition of a saturated sodium bicarbonate aqueous solution. To afford the desired product, the mixture was extracted with ethyl acetate and the combined organic layers were washed with brine, dried over anhydrous sodium sulfate, filtered and the filtrate was evaporated under reduced pressure. Spectral data of compound **S1** was in accordance with previous reports.[6]

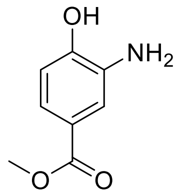

S1

Methyl 3-amino-4-hydroxybenzoate S1: brown solid, 3.85 g, 88 % yield. **^{1}H NMR** (300 MHz, $CDCl_3$): δ 7.49 – 7.40 (m, 2H), 6.75 (d, *J* = 6.0 Hz, 1H), 3.87 (s, 3H).

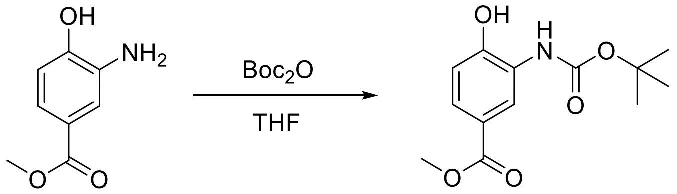

S1 **S2**

The procedure used was adapted from one previously reported.[5] In a flame-dried round-bottomed flask, intermediate **S1** (2.0 g, 11.96 mmol) was dissolved in dry tetrahydrofuran (34.0 mL). Then, di-tert-butyl carbonate (2.5 g, 14.36 mmol) was added and the reaction was stirred overnight at room temperature. Then, the solvent was evaporated and the crude was dissolved in water and extracted with ethyl acetate. The organic layer was washed with brine, dried over anhydrous sodium sulfate, filtered and the filtrate was evaporated under reduced pressure. The crude product was purified by column chromatography (0-70 % ethyl acetate in n-hexane) to afford the desired compound. Spectral data of compound **S2** in accordance with previous reports.[7]

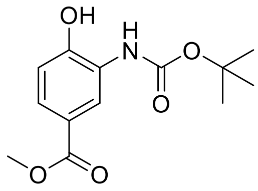

S2

Methyl 3-((tert-butoxycarbonyl)amino)-4-hydroxybenzoate S2: yellow solid, 2.5 g, 77 % yield. **^{1}H NMR** (300 MHz, $CDCl_3$): δ 9.15 (br s, 1H), 7.96 (d, *J* = 3.0 Hz, 1H), 7.70 (dd, *J* = 9.0, 3.0 Hz, 1H), 7.02 (br s, 1H), 6.96 (d, *J* = 9.0 Hz, 1H), 3.87 (s, 3H), 1.53 (s, 9H).

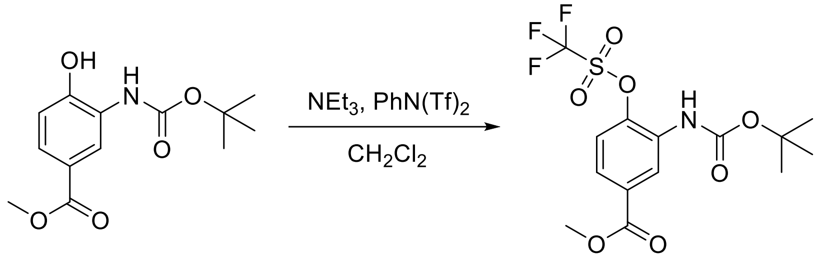

S2 **S3**

The procedure used was adapted from one previously reported.[8] In a flame-dried round-bottomed flask, intermediate **S2** (2.0 g, 7.48 mmol) was dissolved in dry dichloromethane (74.1 mL) and dry triethylamine (3.13 mL, 22.45 mmol) was added under argon atmosphere. Then, the solution was cooled at 0-5 °C and phenyl triflimide (3.48 g, 9.73 mmol) was added. The reaction mixture was allowed to stir at 0-5 °C for 2 h. After water addition, the reaction was extracted with dichloromethane and the organic phase was dried over anhydrous sodium sulfate, filtered and the filtrate was evaporated under reduced pressure. The resulting crude was purified by silica gel column chromatography (0-80 % ethyl acetate in n-hexane) to afford the title compound.

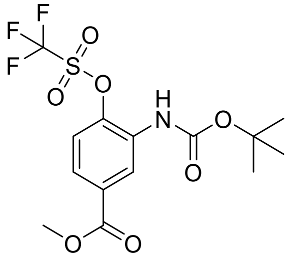

S3

methyl 3-((tert-butoxycarbonyl)amino)-4-(((trifluoromethyl)sulfonyl)oxy)benzoate S3: yellow solid, 2.5 g, 83 % yield. **^{1}H NMR** (300 MHz, $CDCl_3$): δ 8.75 (d, *J* = 3.1 Hz, 1H), 7.78 (dd, *J* = 8.7, 2.1 Hz, 1H), 7.35 (d, *J* = 8.6 Hz, 1H), 6.71 (s, 1H), 3.92 (s, 3H), 1.53 (s, 9H). **^{13}C NMR** (75 MHz, $CDCl_3$): δ 165.49, 151.89, 141.62 (d, *J* = 7.3 Hz), 130.91 (d, *J* = 18.8 Hz), 125.97, 125.14, 124.31, 123.39, 121.36, 120.52 (d, *J* = 23.7 Hz), 116.43, 82.05, 52.53, 28.49 (d, *J* = 55.3 Hz). **HRMS:** *m/z* $[M+H]^+$ Calculated for $C_{14}H_{16}F_3NO_7S$ = 400.0672; found = 400.0670.

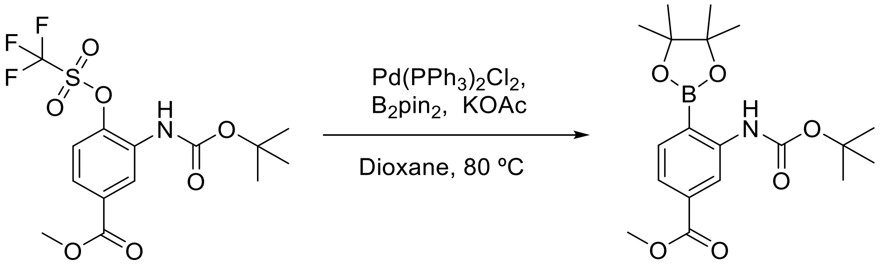

S3 **S4**

The procedure used was adapted from one previously reported.[8] In a flame-dried Schlenk flask, intermediate **S3** (700 mg, 1.753 mmol), potassium acetate (516 mg, 5.26 mmol), bis(pinacolato)diborane (579

mg, 2.279 mmol) and bis(triphenylphosphine)palladium(II) dichloride were suspended in dry dioxane (5.9 mL) under argon atmosphere. After degassed for 15 min, the reaction was stirred at 80 °C overnight, in an oil bath. After cooling to room temperature, the reaction was filtered by celite and the filtrate evaporated under reduced pressure. The resulting crude was purified by silica gel column chromatography (0-50 % ethyl acetate in n-hexane) to give the expected product.

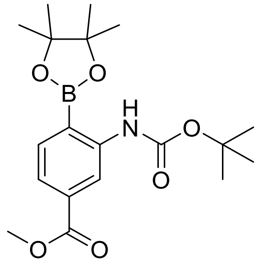

S4

methyl 3-((tert-butoxycarbonyl)amino)-4-(4,4,5,5-tetramethyl-1,3,2-dioxaborolan-2-yl)benzoate S4: white solid, 520 mg, 79 % yield. **^{1}H NMR** (300 MHz, $CDCl_3$): δ 8.79 (d, *J* = 1.5 Hz, 1H), 8.68 (s, 1H), 7.78 (d, *J* = 7.8 Hz, 1H), 7.64 (dd, *J* = 7.7, 1.5 Hz, 1H), 3.91 (s, 3H), 1.54 (s, 9H), 1.37 (s, 12H). **^{13}C NMR** (75 MHz, $CDCl_3$): δ 167.06, 153.54, 152.94, 145.25, 136.80, 136.10, 133.70, 129.31, 126.71, 123.21, 122.33, 118.25, 117.18, 115.45, 84.57, 80.19, 52.15, 28.28, 24.81. **HRMS:** *m/z* $[M+H]^+$ Calculated for $C_{19}H_{28}BNO_6$ = 378.2082; found = 378.2090.

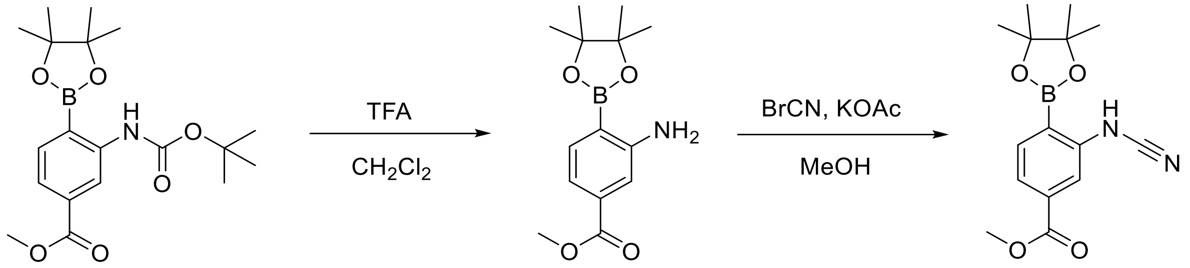

S4 **S5** **8**

The procedure used was adapted from one previously reported.[2,9] In a round bottom flask, trifluoroacetic acid (123 µL, 1.590 mmol) was added dropwise to a solution of the intermediate **S4** (100 mg, 0.265 mmol) in dichloromethane (530 µL) at 0 °C. The reaction was moved to room temperature and stirred for 3 h. Then, the solvent was evaporated partly under reduced pressure and saturated sodium bicarbonate aqueous solution was added to adjust the pH to 9-10. The mixture was extracted with ethyl acetate and the organic phase was washed with brine, dried over anhydrous sodium sulfate, filtered and the filtrate was evaporated under reduced pressure. The compound **S5** obtained was used in the next step without purification: in a round bottom flask, intermediate **S5** (100 mg, 0.361 mmol) and potassium acetate (56.7 mg, 0.577 mmol) were dissolved in methanol (2.41 mL). Cyanogen bromine (115 mg, 1.08 mmol) was added at 0 °C and the reaction was stirred at room temperature for 16 h. Then, the solvent was evaporated and the crude was purified by silica gel column chromatography (0-10 % MeOH in CH_2Cl_2) to afford the desired compound.

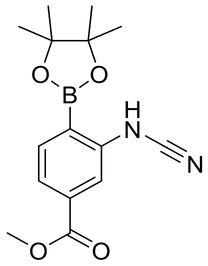

8

methyl 3-cyanamido-4-(4,4,5,5-tetramethyl-1,3,2-dioxaborolan-2-yl)benzoate 8: orange solid, 56 mg, 52 % yield. **^{1}H NMR** (300 MHz, $CDCl_3$): δ 8.10 (s, 1H), 7.85 – 7.67 (m, 2H), 7.62 (dd, *J* = 7.7, 1.4 Hz, 1H), 3.86 (s, 3H), 1.31 (s, 12H). **^{13}C NMR** (75 MHz, $CDCl_3$): δ 166.09, 144.22, 136.79, 134.62, 129.32, 126.82, 123.20, 114.89, 110.48, 85.23, 52.40, 24.77. **HRMS:** *m/z* $[M+H]^+$ Calculated for $C_{15}H_{19}BN_2O_4$ = 303.1511; found = 303.1516.

3. Synthesis of Benzodiazaborine **2**

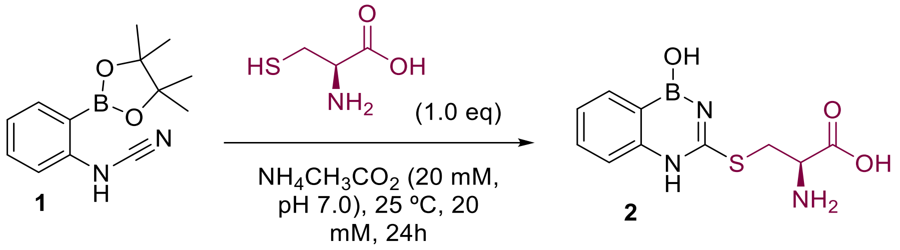

1.0 equivalent of *L*-cysteine (100 mM, 1.0 mL, 0.100 mmol) was added to a 20 mM solution of cyanamide **1** (100 mM, 1.0 mL, 0.100 mmol) in ammonium acetate 20 mM at pH 7.0 (5.00 mL). The reaction was stirred at 25 °C, for 24 h. Then, the mixture was purified by semi-preparative HPLC to afford the title compound. The HPLC runs were carried out with a gradient of A (Milli Q water containing 0.1 % v/v Formic acid, FA) and B (acetonitrile containing 0.1 % v/v FA, Honeywell HPLC-grade). The mobile phase was t = 0 min, 5 % B; t = 10 min, 50 % B; t = 12 - 13 min, 95 % B; t = 14 min, 5 % B; t = 15 min, stop at a flow rate of 5.0 mL/min. BDAB **2** : RT 5.84 min, detection UV (254 nm).

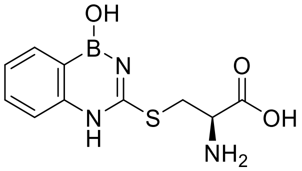

***S*-(1-hydroxy-1,4-dihydrobenzo[c][1,5,2]diazaborinin-3-yl)cysteine 2**: white solid, 2 mg, 75 % yield. **^{1}H NMR** (300 MHz, D_2O): δ 7.76 (m, 1H), 7.53 (t, *J* = 7.7 Hz, 1H), 7.40 (t, *J* = 7.4 Hz, 1H), 7.22 (d, *J* = 7.8 Hz, 1H), 4.32 (m, 1H), 3.87 (d, *J* = 5.1 Hz, 2H). **^{13}C NMR** (101 MHz, D_2O): δ 158.76, 139.38, 131.51, 129.90, 126.15, 116.31, 53.82, 32.43. **^{11}B NMR** (128 MHz, D_2O): δ 6.92. **HRMS:** *m/z* $[M+H]^+$ Calculated for $C_{10}H_{12}BN_3O_3S$ = 266.0765; found = 266.0756.

4. Optimization of Benzodiazaborine **2** Formation

4.1 pH Study

1.0 equivalent of *L*-cysteine (100 mM, 1.0 mL, 0.100 mmol) was added to 20 mM solutions of cyanamide **1** (100 mM, 1.0 mL, 0.100 mmol) in ammonium acetate 20 mM at pH 5.0, 7.0 and 8.0, respectively (5.00 mL). The three reactions were stirred at 25 °C and monitored by ESI-MS in Positive Mode. The pH was controlled after the preparation of each solution and adjusted if necessary.

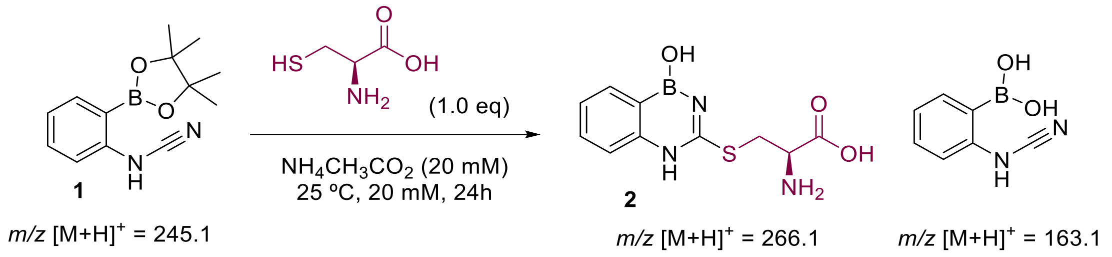

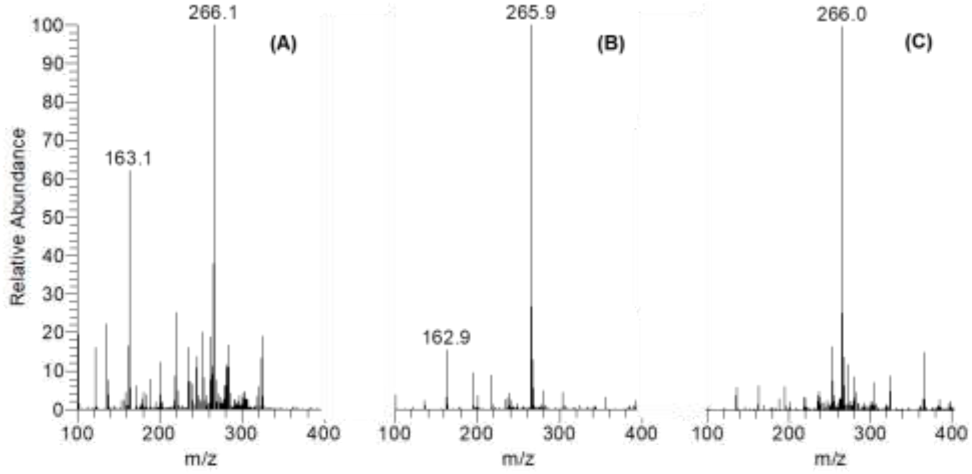

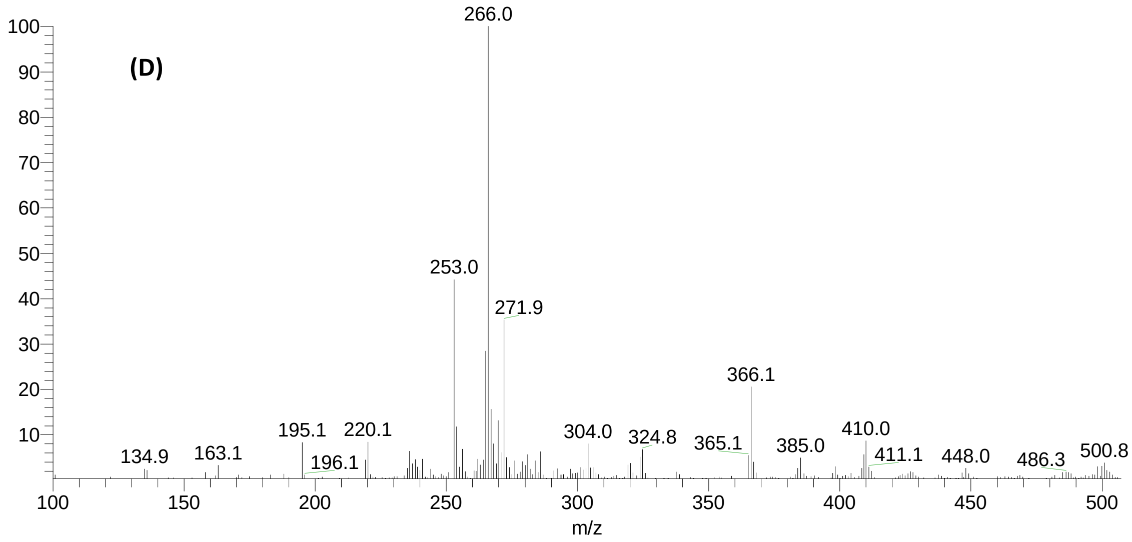

Figure S2. ESI$^+$-LRMS spectra of reaction between cyanamide **1** and *L*-cysteine after 24 h at **(A)** pH 5.0, **(B)** 7.0 and **(C)** 8.0. **(D)** Zoom in of spectrum C; At pH 8.0, cyanamide **1** (*m/z* [M-pin+H]$^+$ = 163.1) is only detected as a residual peak.

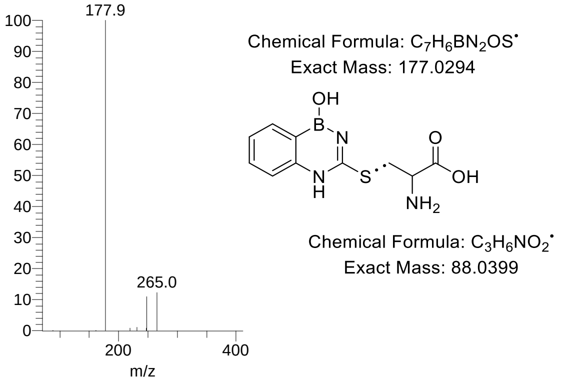

Figure S3. MS/MS fragmentation of compound **2** (*m/z* [M+H]$^+$ 266.1 peak). The proposed structure of fragment *m/z* 177.0 is coherent with thiol modification.

4.2. Reaction Concentration Study

1.0 equivalent of *L*-cysteine (100 mM, 1.0 mL, 0.100 mmol) was added to 2, 10 and 20 mM solutions of cyanamide **1** (100 mM, 1.0 mL, 0.100 mmol) in ammonium acetate 20 mM at pH 7.0 (5.0 mL). The three reactions were stirred at 25 °C and monitored by ESI-MS in Positive Mode.

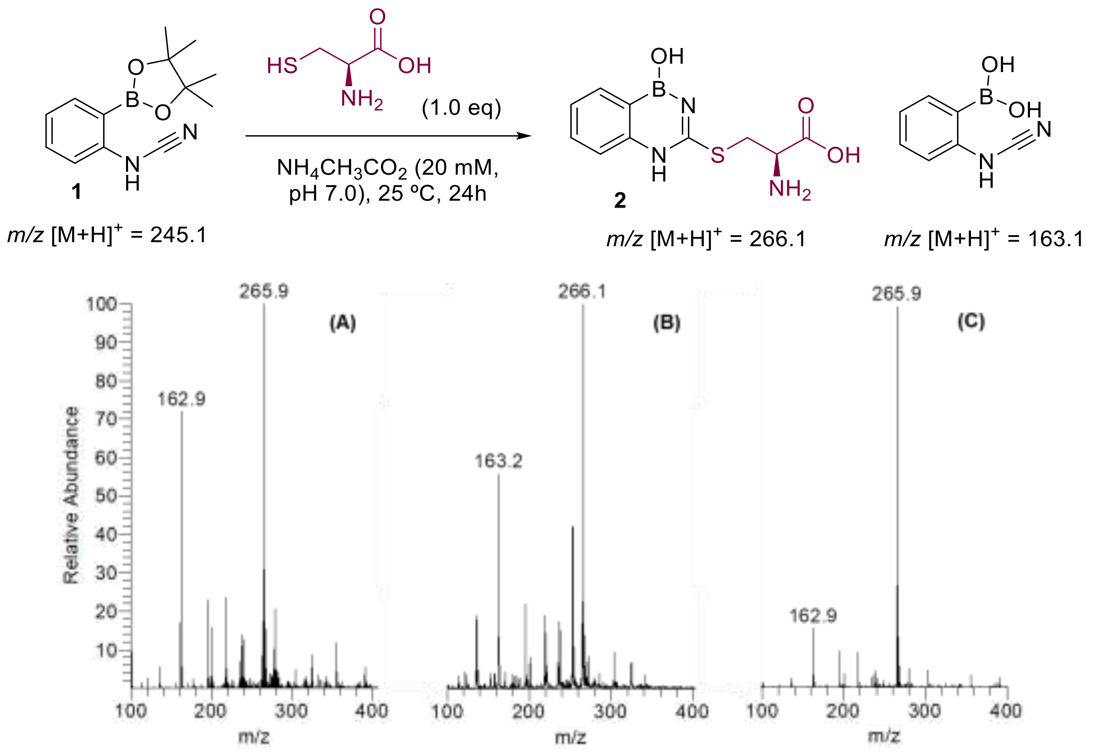

Figure S4. ESI$^+$-LRMS spectra of reaction between cyanamide **1** and *L*-cysteine after 24 h at **(A)** 2.0 mM, **(B)** 10 mM and **(C)** 20 mM.

5. Kinetic Studies of Benzodiazaborine **2** Formation

The following reaction was performed under pseudo-first order conditions in a NMR tube: 10 equivalents of *L*-cysteine (30 mg, 0.248 mmol) were added to a 50 mM solution of cyanamide **1** (6.04 mg, 0.025 mmol) in deuterated ammonium acetate 20 mM at pH 7.0 (0.50 mL). The formation of BDAB **2** was monitored by ^{1}H-NMR spectroscopy over 26 h at room temperature.

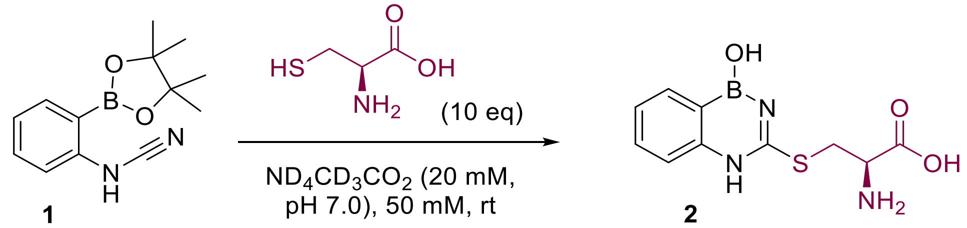

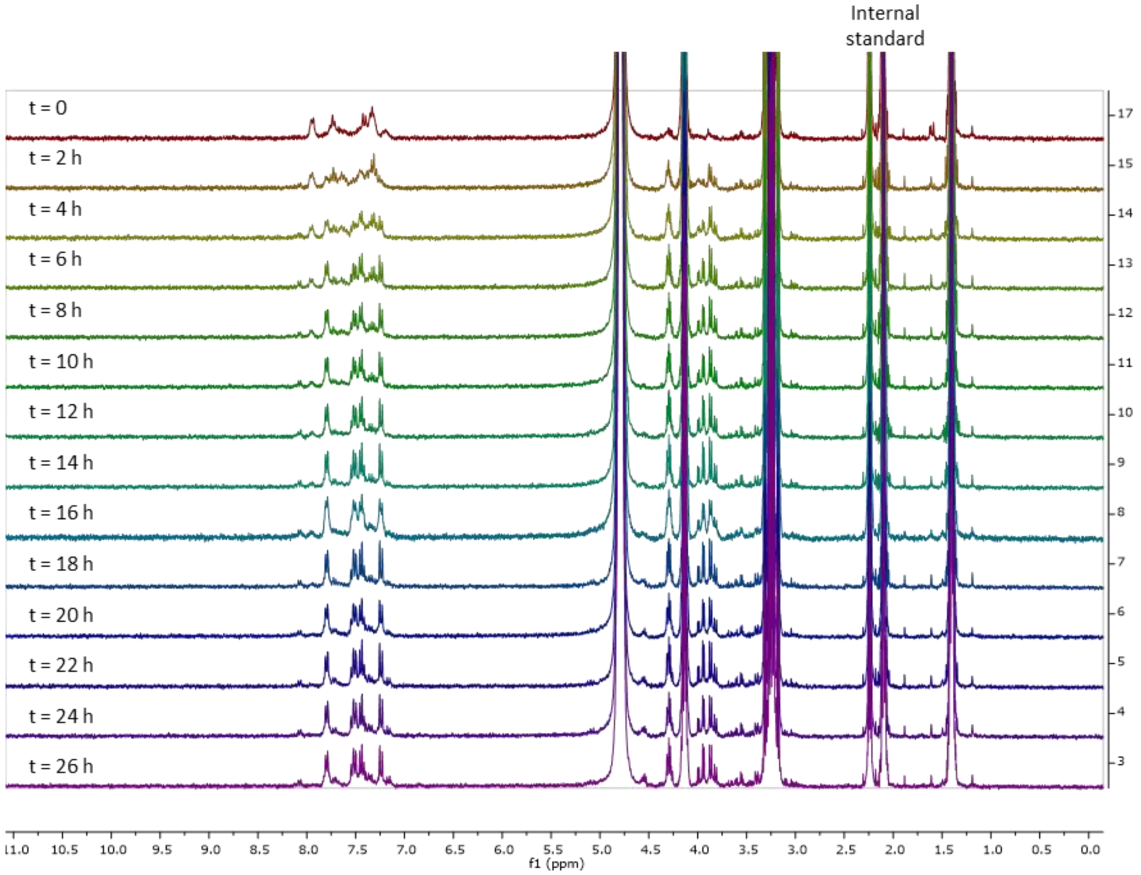

Figure S5. ^{1}H-NMR spectra of compound **2** formation as a function of time. Initial [**1**] = 50 mM, internal standard = deuterated ammonium acetate.

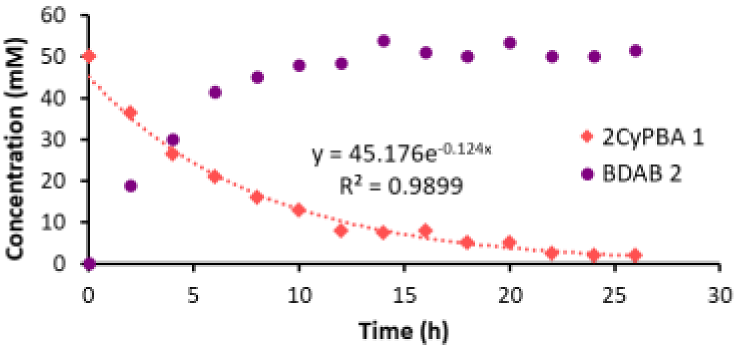

Figure S6. ^{1}H-NMR kinetic study**.** Cyanamide **1** and diazaborine **2** concentrations versus time data were acquired from the integration of the respective ^{1}H-NMR signals relative to deuterated ammonium acetate, utilized as an internal standard.

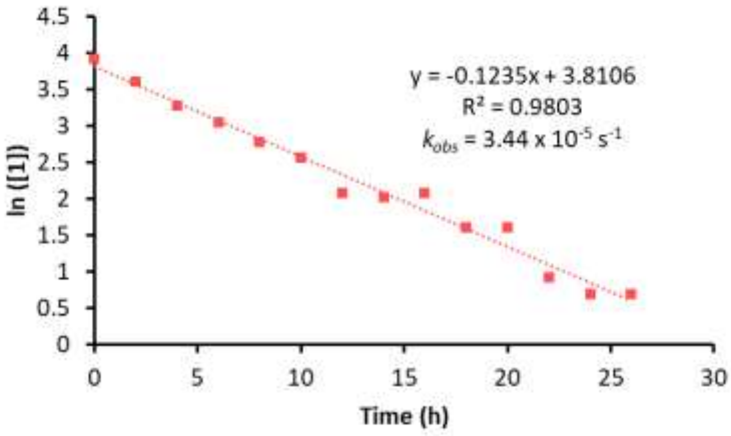

Figure S7. Linear fitting of cyanamide **1** kinetic data using the first order kinetic model. The plot of ln([**1**]) (mM) versus time (h) was used to determine the pseudo-first order constant (k_{obs}). 2CyPBA **1** concentration versus time data were acquired from the integration of the respective ^{1}H-NMR signals relative to deuterated ammonium acetate, utilized as an internal standard.

6. Benzodiazaborine **2** Stability Studies

6.1. Stability in PBS pH 7.4

Triplicated solutions of benzodiazaborine **2** (10 mM, 10 µL, 0.1 µmol) in PBS pH 7.4 (1.0 mL) were prepared and incubated at 25 °C for five days. 20 µL aliquots were taken over time for LC-MS analysis. The HPLC runs were carried out with a gradient of A (Milli Q water containing 0.1 % v/v Formic acid, FA) and B (acetonitrile containing 0.1 % v/v FA, Honeywell HPLC-grade). The mobile phase was t = 0-1 min, 5 % B; t = 10-11 min, 95.5 % B; t = 12 min, 5 % B; t = 15 min, stop at a flow rate of 0.2 mL / min. Diazaborine: RT 5.84 min, detection EIC. The peak areas of the EIC of **2** over time were converted into remaining concentration based on the following calibration curve (base peak *m/z* 266.0765, within 5 ppm range).

A

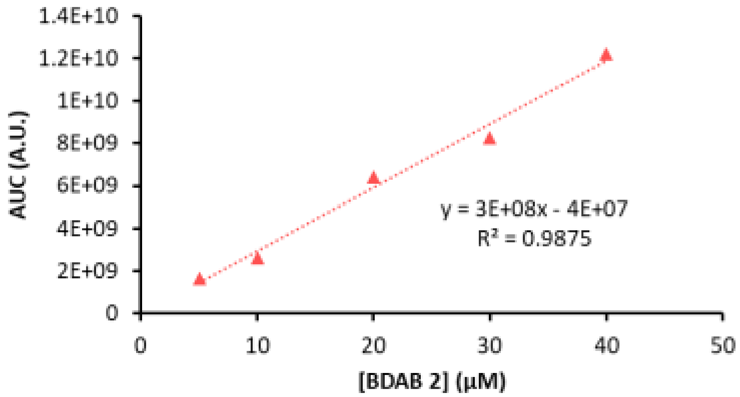

B

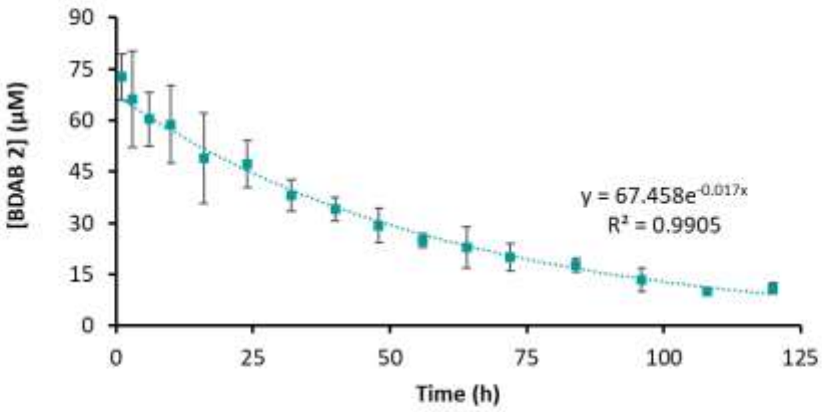

C

$y = A_1*e^{-kx}$	
A_1	67.46 ± 10.60
k_{obs}	0.017 ± 0.00094
$t_{1/2}$	40.77 h

Figure S8. Benzodiazaborine **2** stability in PBS pH 7.4. **A** – BDAB **2** calibration curve of EIC intensity (base peak *m/z* 266.0765, within 5 ppm range) *vs* **2** concentration (µM); **B** – The BDAB **2** hydrolysis kinetics was performed in PBS pH 7.4 to determine the k_{obs} from the exponential plot of **2** concentration *vs* time (h). **C** - Calculated k_{obs} and $t_{1/2}$ from the triplicate analysis. The k_{obs} corresponds to the k value from the nonlinear fitting equation: $y= A_1*e^{-kx}$ and $t_{1/2} = \ln 2 / k_{obs}$).

6.2. Stability in Presence of Glutathione

Triplicated solutions of benzodiazaborine **2** (10 mM, 10 µL, 0.1 µmol) in PBS pH 7.4 (1.0 mL) were prepared and incubated with 10 equivalents of glutathione (50 mM, 20 µL, 1.0 µmol) at 25 °C for three days. 20 µL aliquots were taken over time for LC-MS analysis. The HPLC runs were carried out with a gradient of A (Milli Q water containing 0.1 % v/v Formic acid, FA) and B (acetonitrile containing 0.1 % v/v FA, Honeywell HPLC-grade). The mobile phase was t = 0-1 min, 5 % B; t = 10-11 min, 95.5 % B; t = 12 min, 5 % B; t = 15 min, stop at a flow rate of 0.2 mL/min. Diazaborine: RT 5.84 min, detection EIC. The peak areas of the EIC of **2** over time were converted into remaining concentration based on the following calibration curve (base peak *m/z* 266.0765, within 5 ppm range).

A

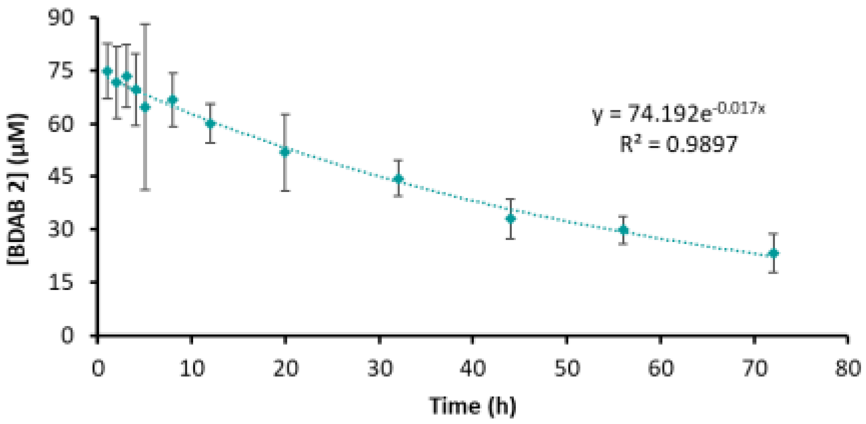

B

$y = A_1 * e^{-kx}$	
A_1	74.19 ± 10.37
k_{obs}	0.017 ± 0.00124
$t_{1/2}$	40.77 h

Figure S9. Benzodiazaborine **2** stability in presence of glutathione. **A** – The BDAB **2** hydrolysis kinetics was performed in PBS pH 7.4 in presence of 10 equivalents of GSH to determine the k_{obs} from the exponential plot of **2** concentration *vs* time (h). **B** - Calculated k_{obs} and $t_{1/2}$ from the triplicate analysis. The k_{obs} corresponds to the k value from the nonlinear fitting equation: $y= A_1*e^{-kx}$ and $t_{1/2} = \ln 2 / k_{obs}$).

7. ESI-MS Mechanistic Studies

The reactions between cyanamides **1**, **3**, **6** or **8** and cysteine models (*L*-cysteine and *N*-acetyl cysteine) were performed according to the following protocol: 1.0 equivalent of the cysteine model *L*-cysteine or *N*-acetyl cysteine (100 mM, 100 µL, 10.00 µmol) was added to a 2 mM solution of the respective cyanamide (100 mM, 100 µL, 10.00 µmol) in ammonium acetate solution 20 mM, pH 7.0 (500 µL). The reaction was stirred at 25 °C for 24 h and monitored by ESI-MS in Positive Mode. The reactions with cyanamides **1** and **3** were also monitored by LC-HRMS in Positive Mode. The HPLC runs were carried out with a gradient of A (Milli Q water containing 0.1 % v/v Formic acid, FA) and B (acetonitrile containing 0.1 % v/v FA, Honeywell HPLC-grade). The mobile phase was t = 0-1 min, 5 % B; t = 10-11 min, 95.5 % B; t = 12 min, 5 % B; t = 15 min, stop at a flow rate of 0.2 mL/min, detection EIC.

7.1. ESI-MS Assays with Cyanamide **1** and *N*-Acetyl Cysteine

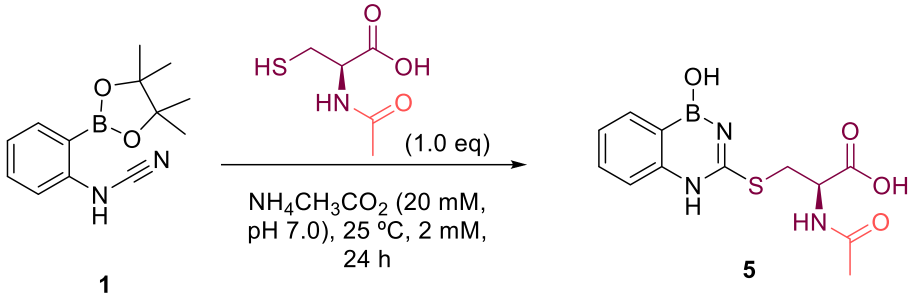

m/z $[M+H\text{-}pin]^+$= 163.0673

m/z $[M+H]^+$= 308.0871

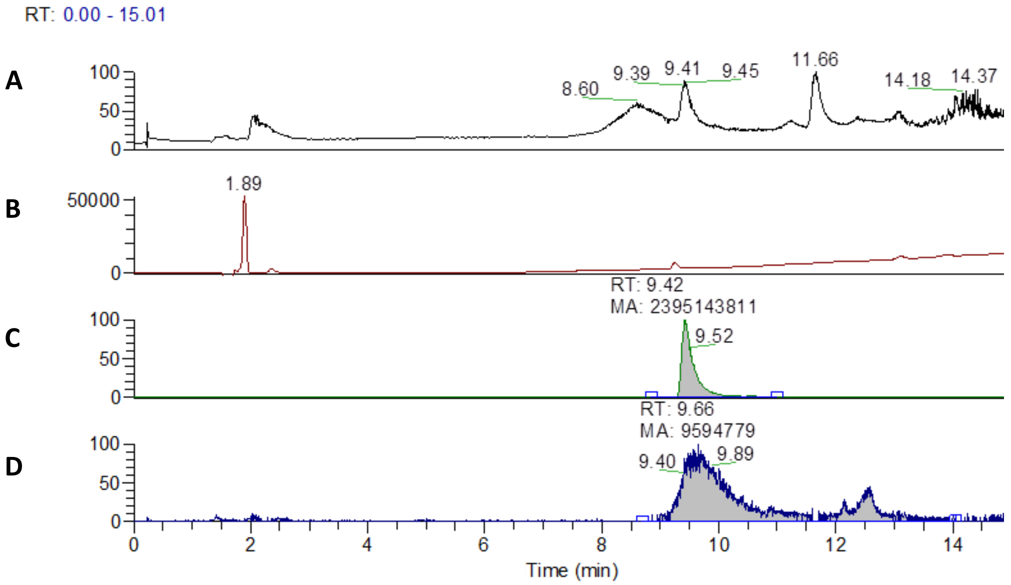

Figure S10. LC-MS chromatograms of BDAB **5** and cyanamide **1**. **A** – Reaction mixture TIC after 24 h; **B** – Reaction detection at 210 nm, after 24 h; **C** – Unreacted cyanamide **1** EIC (base peak *m/z* 163.0673); **D** – Residual conjugate **5** EIC (base peak *m/z* 308.0871) after 24 h reaction.

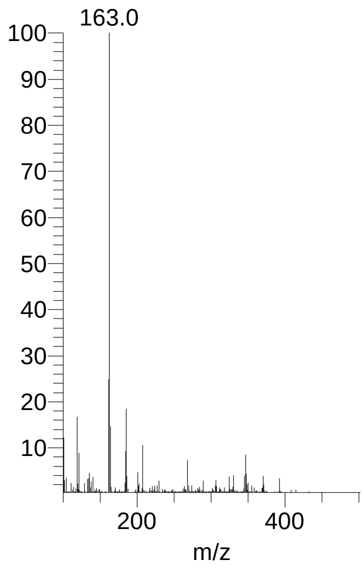

Figure S11. ESI^+-LRMS spectrum of reaction between 2CyPBA **1** and *N*-acetyl cysteine after 24 h.

7.2. ESI-MS assays with Cyanamide **3** and *L*-Cysteine

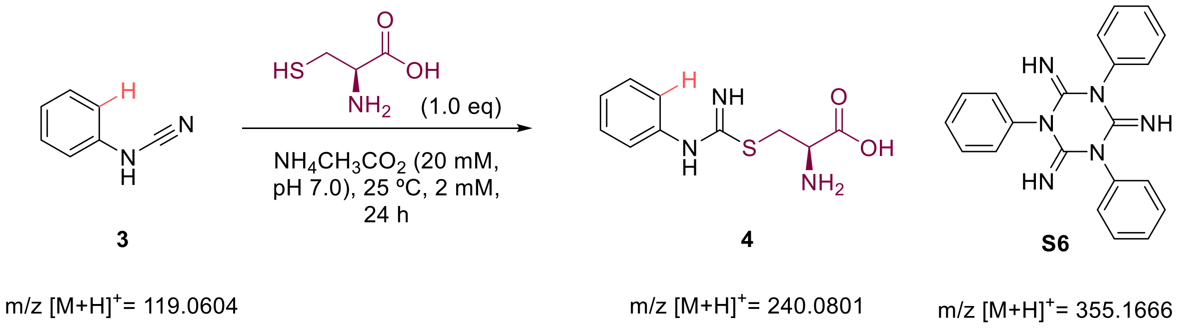

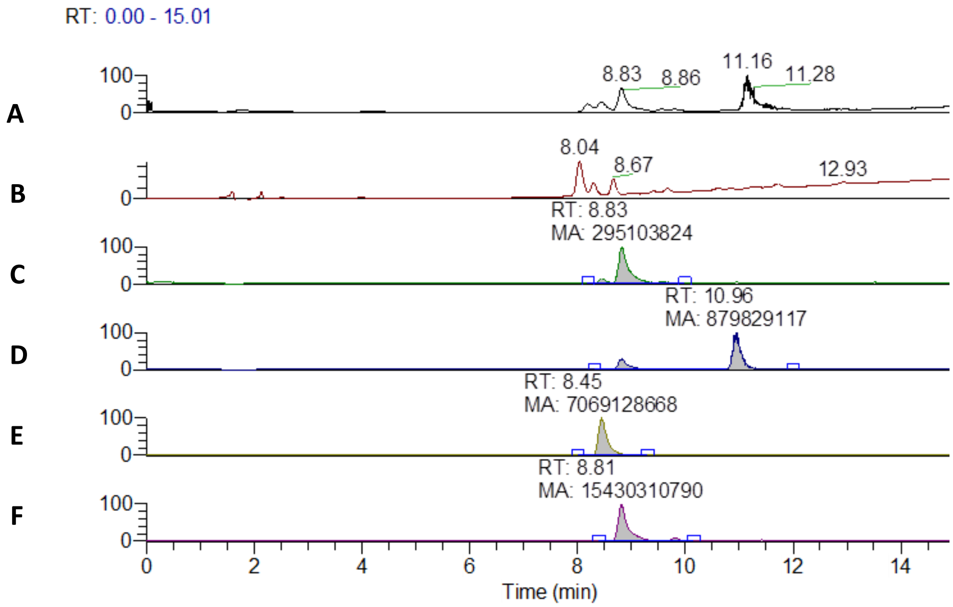

Figure S12. LC-MS chromatograms of thiourea **4** and cyanamide **3**. **A** – Reaction mixture TIC after 24 h; **B** – Reaction detection at 210 nm, after 48 h; **C** – Unreacted cyanamide 3 EIC after 24 h; **D** – Reaction t_0 – unreacted cynamide 3 EIC (base peak *m/z* 119.0604); **E** – Thiourea 4 EIC (base peak *m/z* 240.0801) after 24 h reaction. **F** – Trimer S6 EIC (base peak *m/z* 355.1666) after 24 h reaction.

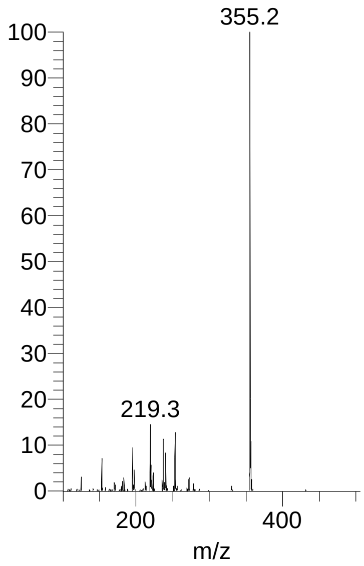

Figure S13. ESI^{+}-LRMS spectrum of reaction between phenylcyanamide **3** and *L*-cysteine after 24 h.

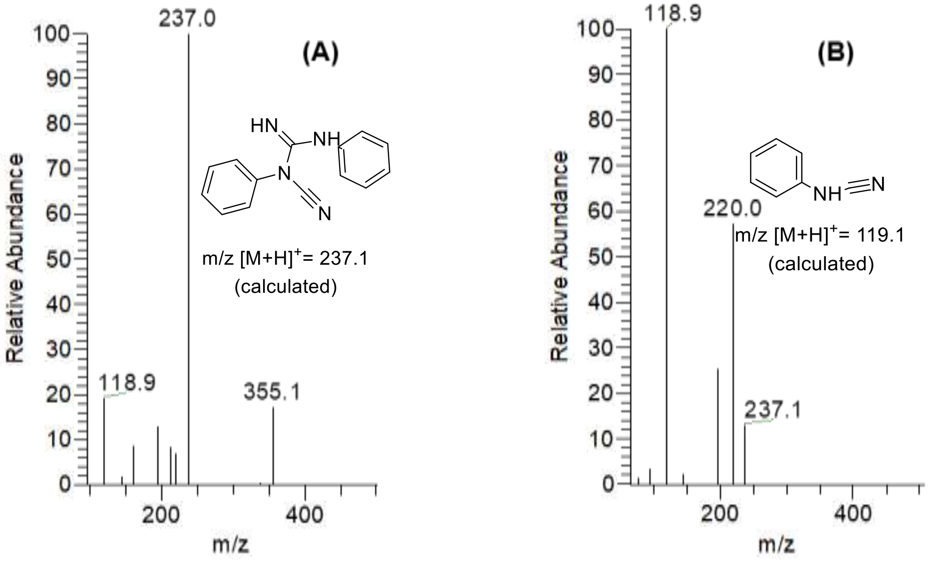

Figure S14. MS/MS fragmentation of **(A) S6** (*m/z* $[M+H]^+$ 355.2 peak) and **(B)** *m/z* $[M+H]^+$ 237.1 peak of **S6** fragmentation. The proposed structures of fragments *m/z* 237.0 and 118.9 are coherent with cyanamide polymerization.

7.3. ESI-MS Assays with Methylated Cyanamide **6** and *L*-Cysteine

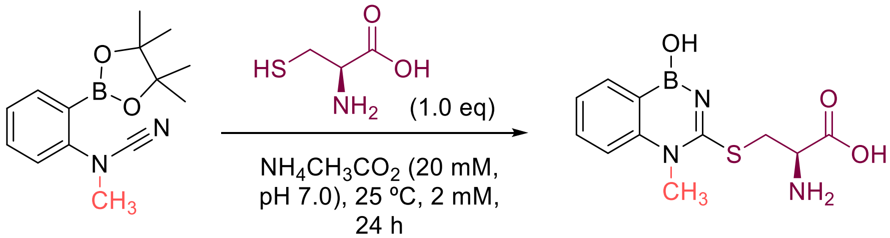

6

7

m/z $[M+H\text{-}pin]^+$= 177.1

m/z $[M+H]^+$= 280.1

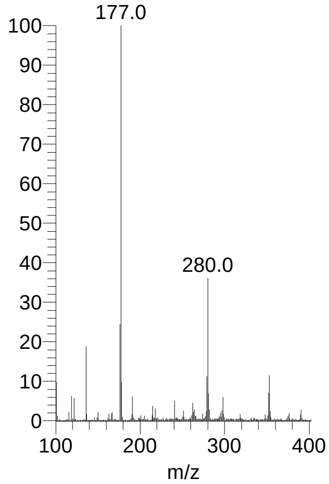

Figure S15. ESI$^+$-LRMS spectrum of reaction between 2CyPBA **6** and *L*-cysteine after 24 h.

7.4. ESI-MS Assays with Cyanamide **8** and *L*-Cysteine

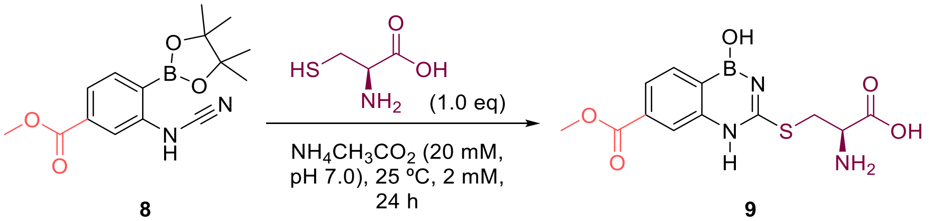

8 **9**

m/z [M+H-pin]$^+$ = 221.1 *m/z* [M+H]$^+$ = 324.1

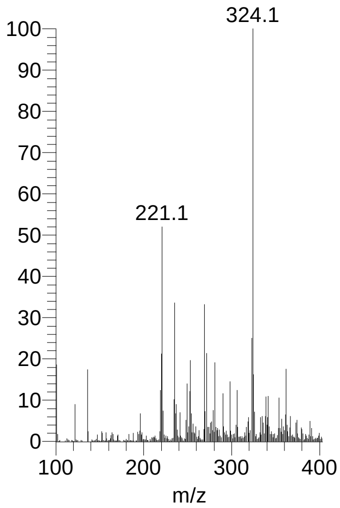

Figure S16. ESI$^+$-LRMS spectrum of reaction between 2CyPBA **6** and *L*-cysteine after 24 h.

8. General Procedure for the ESI-MS Assays with Cys Dipeptides and Cyanamide **1**

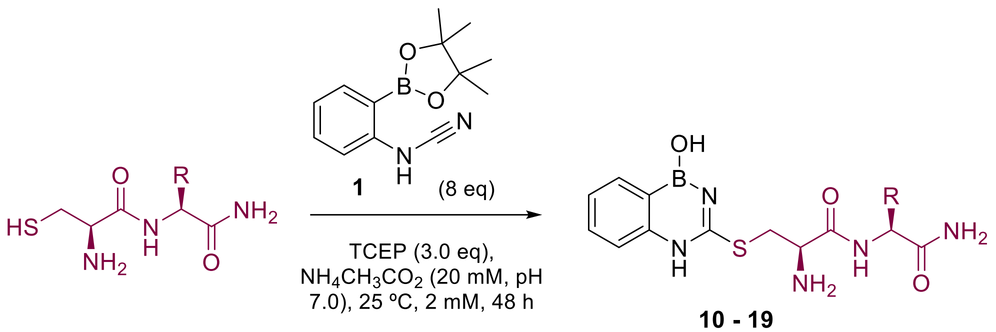

3.0 equivalents of tris-(2-carboxyethyl)phosphine hydrochloride (TCEP, 50 mM, 20 µL, 1.0 µM) were added to a 2 mM solution of dipeptide (Cys-AA, 50 mM, 10 µL, 0.5 µM) in ammonium acetate solution 20 mM, pH 8.0 (250 µL) and the mixture reacted at 25 °C for 1 h. Afterwards, 8.0 equivalents of cyanamide **1** (100 mM, 40 µL, 4.0 µM) were added. The reactions were monitored by ESI-MS at 5 min and 48 h and by LC-HRMS in Positive Mode. The HPLC runs were carried out with a gradient of A (Milli Q water containing 0.1 % v/v Formic acid, FA) and B (acetonitrile containing 0.1 % v/v FA, Honeywell HPLC-grade). The mobile phase was t = 0-1 min, 5 % B; t = 10-11 min, 95.5 % B; t = 12 min, 5 % B; t = 15 min, stop at a flow rate of 0.2 mL/min, detection EIC.

8.1. Cys-Gly

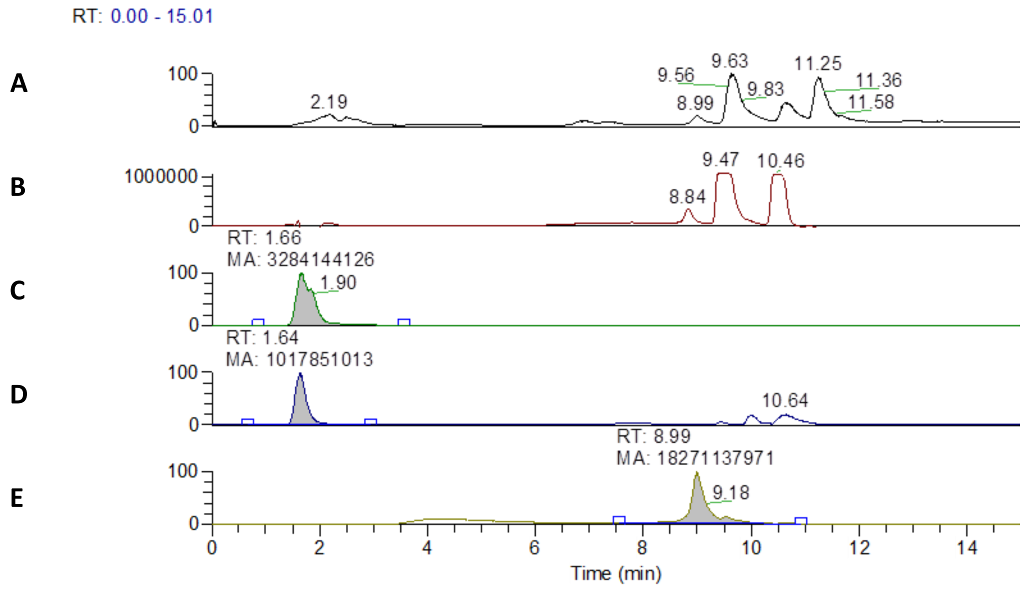

Figure S17. LC-MS chromatograms of Cys-Gly conjugate **10** and Cys-Gly dipeptide. **A** – Reaction mixture TIC after 48 h; **B** – Reaction detection at 210 nm, after 48 h; **C** – Reaction t_0 – unreacted Cys-Gly EIC (base peak *m/z* 178.0645); **D** – Unreacted Cys-Gly EIC after 48 h with 8.0 eq of **1**; **E** – Cys-Gly conjugate **10** EIC (base peak *m/z* 322.1140) after 48 h reaction. Cys-Gly conversion was calculated based on the EIC intensity (AUC) within δ 5 ppm range.

Table S1. Cys-Gly dipeptide conversion in 48 h reaction with cyanamide **1** according to AUC from EIC chromatograms of base peak *m/z* 178.0645, within δ 5 ppm range.

Dipeptide	t_0	48 h	Conversion
Cys-Gly (AUC A.U.)	3284144126	1017851013	69 %

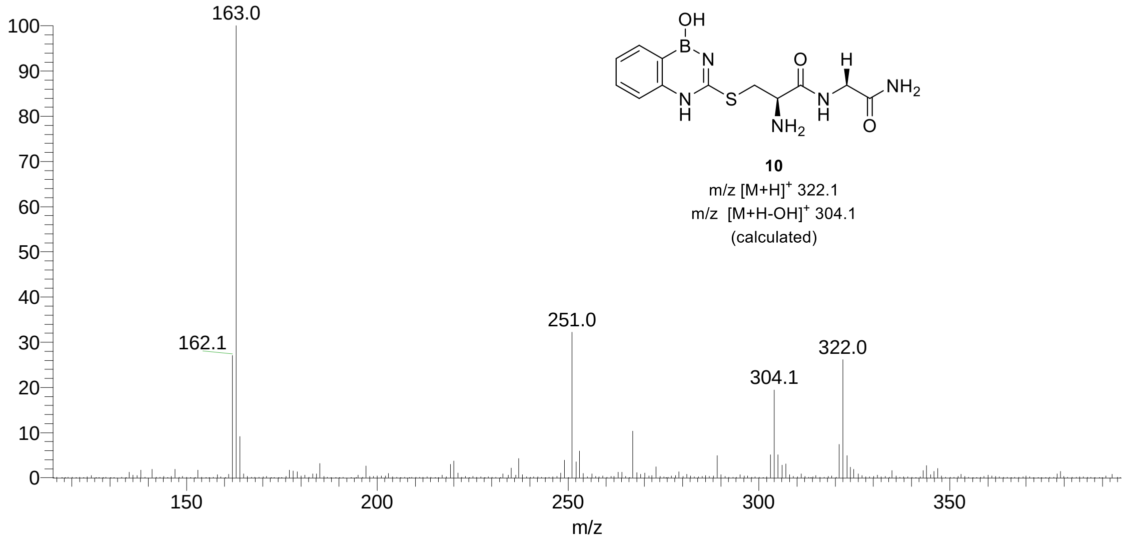

Figure S18. ESI$^+$-LRMS spectrum of reaction between Cys-Gly (calculated $C_5H_{11}N_3O_2S$ *m/z* $[M+H]^+$ 178.1 not found) and cyanamide **1** (calculated $C_7H_7BN_2O_2$ *m/z* $[M\text{-}pin+H]^+$ 163.1, found 163.0), and the resulting product **10** (calculated $C_{12}H_{16}BN_5O_3S$ *m/z* $[M+H]^+$ 322.1, found 322.0; *m/z* $[M+H\text{-}OH]^+$ 304.1).

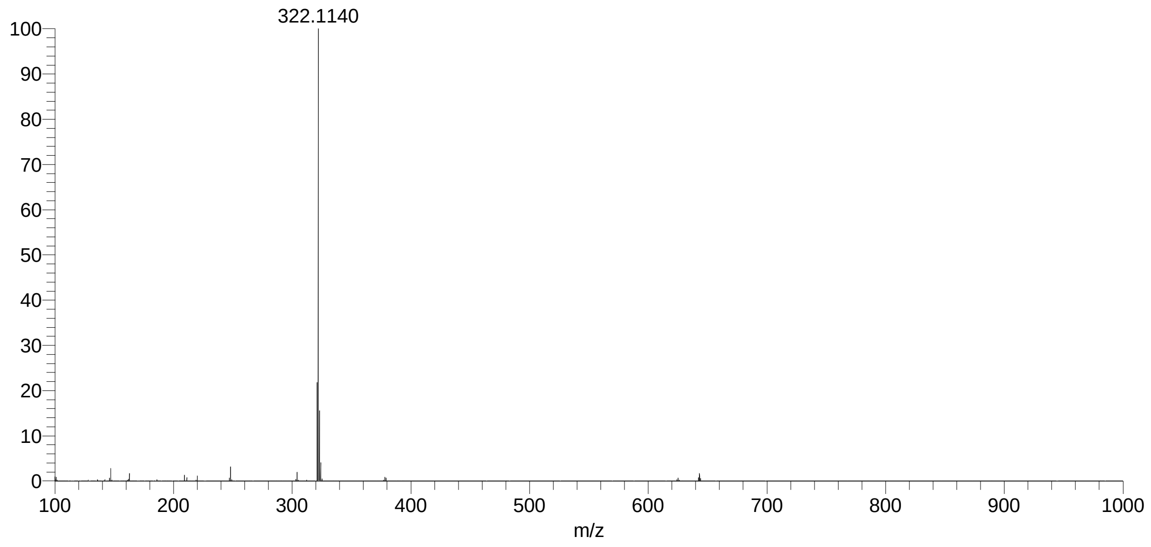

Figure S19. Full ESI$^+$-HRMS spectrum of Cys-Gly conjugate **10** (calculated $C_{12}H_{16}BN_5O_3S$ *m/z* $[M+H]^+$: 322.1140, found 322.1140 from TIC peak at RT 8.99 min).

8.2. Cys-Ala

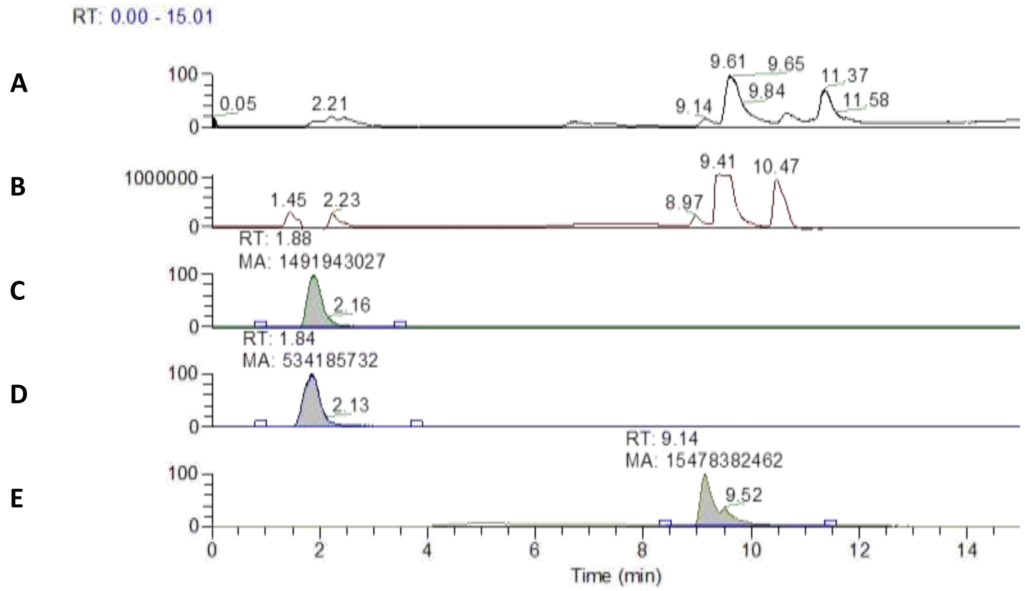

Figure S20. LC-MS chromatograms of Cys-Ala conjugate **11** and Cys-Ala dipeptide. **A** – Reaction mixture TIC after 48 h; **B** – Reaction detection at 210 nm, after 48 h; **C** – Reaction t_0 – unreacted Cys-Ala EIC (base peak *m/z* 192.0801); **D** – Unreacted Cys-Ala EIC after 48 h with 8.0 eq of **1**; **E** – Cys-Ala conjugate **11** EIC (base peak *m/z* 336.1296) after 48 h reaction. Cys-Ala conversion was calculated based on the EIC intensity (AUC) within δ 5 ppm range.

Table S2. Cys-Ala dipeptide conversion in 48 h reaction with cyanamide **1** according to AUC from EIC chromatograms of base peak *m/z* 192.0801, within δ 5 ppm range.

Dipeptide	t_0	48 h	Conversion
Cys-Ala (AUC A.U.)	1491943027	534185732	64 %

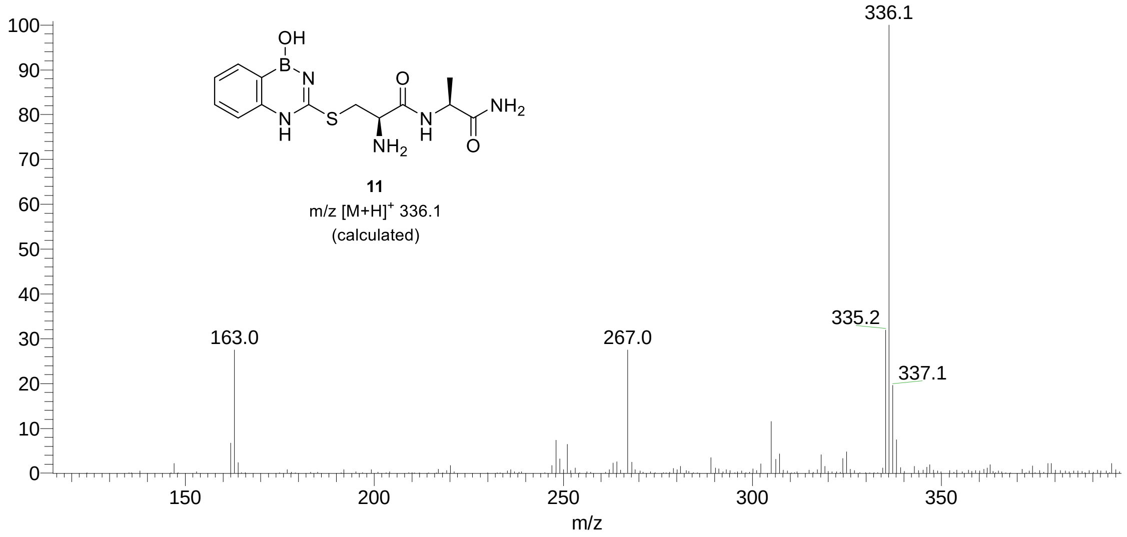

Figure S21. ESI$^+$-LRMS spectrum of reaction between Cys-Ala (calculated $C_6H_{13}N_3O_2S$ *m/z* $[M+H]^+$ 192.1 not found) and cyanamide **1** (calculated $C_7H_7BN_2O_2$ *m/z* $[M\text{-}pin+H]^+$ 163.1, found 163.0), and the resulting product **11** (calculated $C_{13}H_{18}BN_5O_3S$ *m/z* $[M+H]^+$ 336.1, found 336.1).

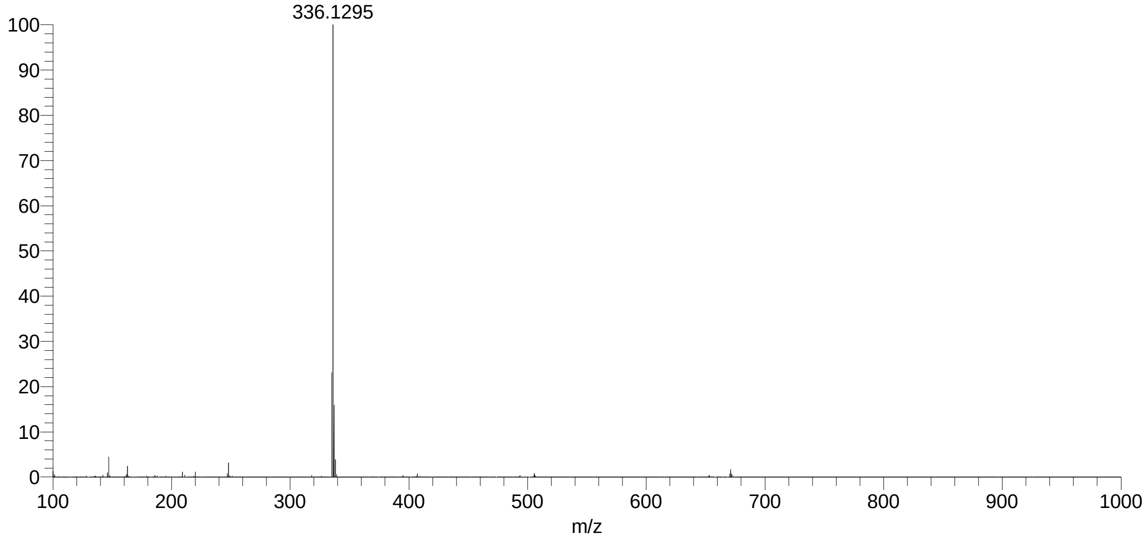

Figure S22. Full ESI$^+$-HRMS spectrum of Cys-Ala conjugate **11** (calculated $C_{13}H_{18}BN_5O_3S$ *m/z* $[M+H]^+$: 336.1296, found 336.1295 from TIC peak at RT 9.14 min).

8.3. Cys-Leu

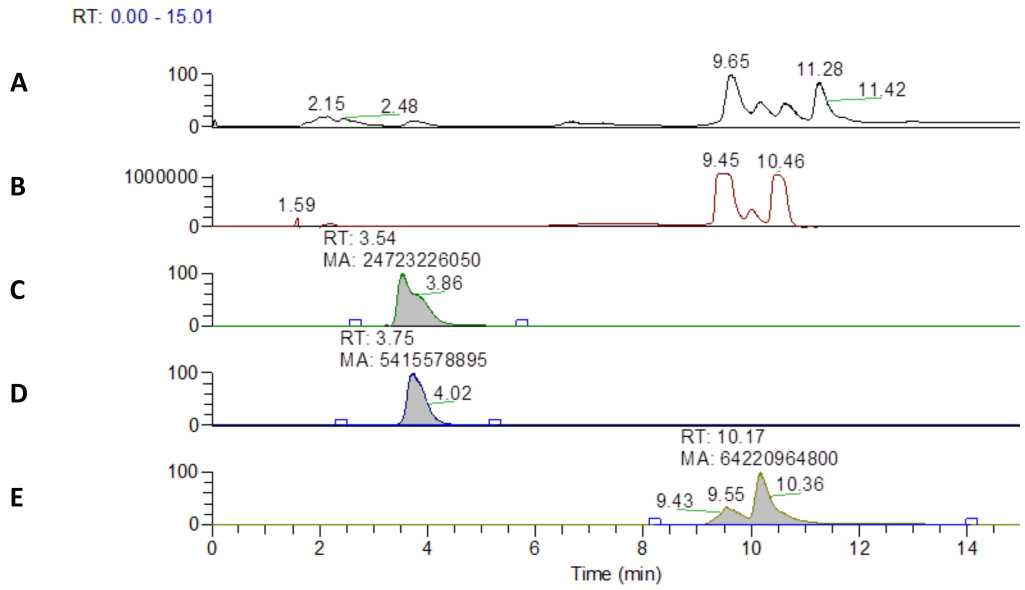

Figure S23. LC-MS chromatograms of Cys-Leu conjugate **13** and Cys-Leu dipeptide. **A** – Reaction mixture TIC after 48 h; **B** – Reaction detection at 210 nm, after 48 h; **C** – Reaction t_0 – unreacted Cys-Leu EIC (base peak *m/z* 234.1271); **D** – Unreacted Cys-Leu EIC after 48 h with 8.0 eq of **1**; **E** – Cys-Leu conjugate **13** EIC (base peak *m/z* 378.1766) after 48 h reaction. Cys-Leu conversion was calculated based on the EIC intensity (AUC) within δ 5 ppm range.

Table S3. Cys-Leu dipeptide conversion in 48 h reaction with cyanamide **1** according to AUC from EIC chromatograms of base peak *m/z* 234.1271, within δ 5 ppm range.

Dipeptide	t_0	48 h	Conversion
Cys-Leu (AUC A.U.)	24723226050	5415578895	78 %

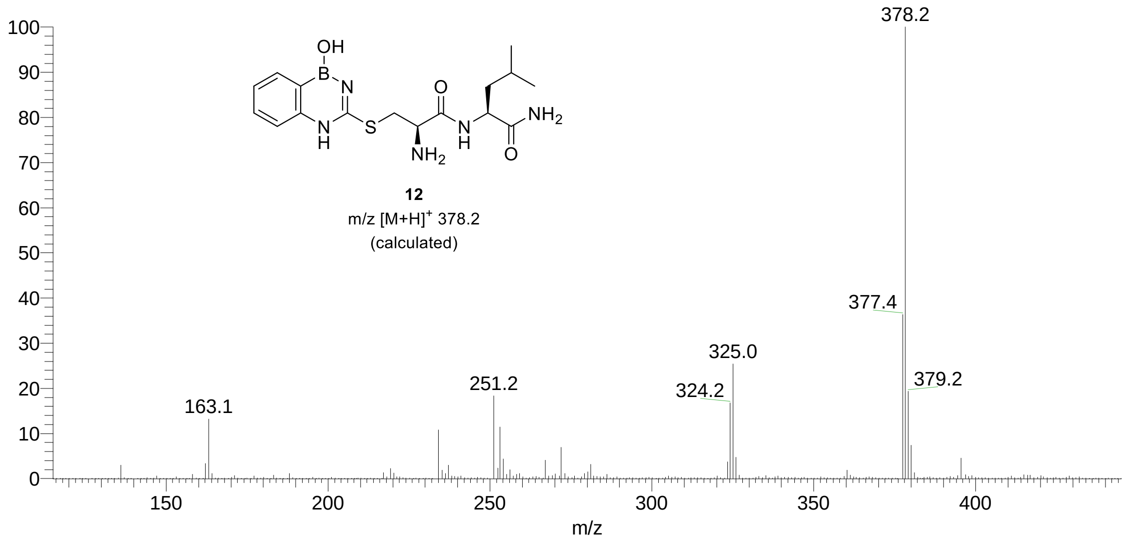

Figure S24. ESI$^+$-LRMS spectrum of reaction between Cys-Leu (calculated $C_9H_{19}N_3O_2S$ *m/z* $[M+H]^+$ 234.1, found 234.1 as a residual peak) and cyanamide **1** (calculated $C_7H_7BN_2O_2$ *m/z* $[M\text{-}pin+H]^+$ 163.1, found 163.1), and the resulting product **12** (calculated $C_{16}H_{24}BN_5O_3S$ *m/z* $[M+H]^+$ 378.2, found 378.2).

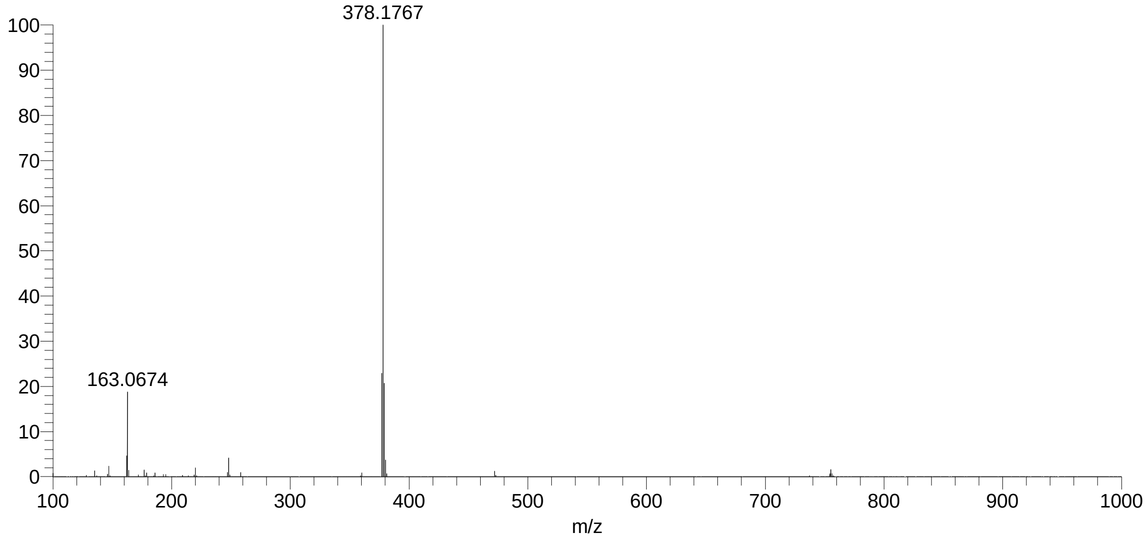

Figure S25. Full ESI$^+$-HRMS spectrum of Cys-Leu conjugate **12** (calculated $C_{16}H_{24}BN_5O_3S$ *m/z* $[M+H]^+$: 378.1766, found 378.1767 from TIC peak at RT 10.17 min).

8.4. Cys-Ser

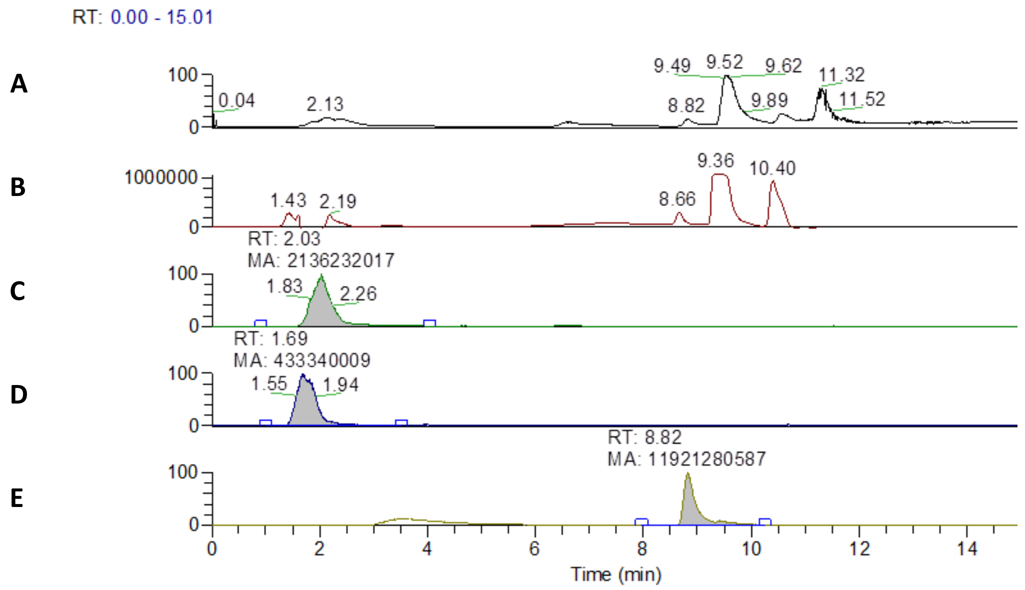

Figure S26. LC-MS chromatograms of Cys-Ser conjugate **13** and Cys-Ser dipeptide. **A** – Reaction mixture TIC after 48 h; **B** – Reaction detection at 210 nm, after 48 h; **C** – Reaction t_0 – unreacted Cys-Ser EIC (base peak *m/z* 208.0750); **D** – Unreacted Cys-Ser EIC after 48 h with 8.0 eq of **1**; **E** – Cys-Ser conjugate **13** EIC (base peak *m/z* 352.1245) after 48 h reaction. Cys-Ser conversion was calculated based on the EIC intensity (AUC) within δ 5 ppm range.

Table S4. Cys-Ser dipeptide conversion in 48 h reaction with cyanamide **1** according to AUC from EIC chromatograms of base peak *m/z* 208.0750, within δ 5 ppm range.

Dipeptide	t_0	48 h	Conversion
Cys-Ser (AUC A.U.)	2136232017	433340009	80 %

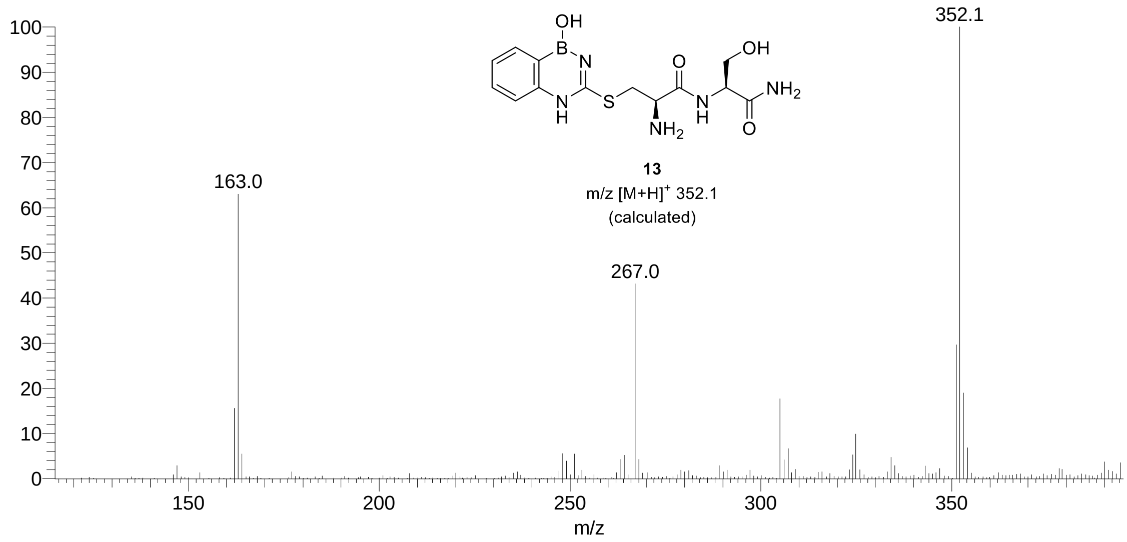

Figure S27. ESI$^+$-LRMS spectrum of reaction between Cys-Ser (calculated $C_6H_{13}N_3O_2S$ *m/z* $[M+H]^+$ 208.1 not found) and cyanamide **1** (calculated $C_7H_7BN_2O_2$ *m/z* $[M\text{-pin}+H]^+$ 163.1, found 163.0), and the resulting product **13** (calculated $C_{13}H_{18}BN_5O_4S$ *m/z* $[M+H]^+$ 352.1, found 352.1).

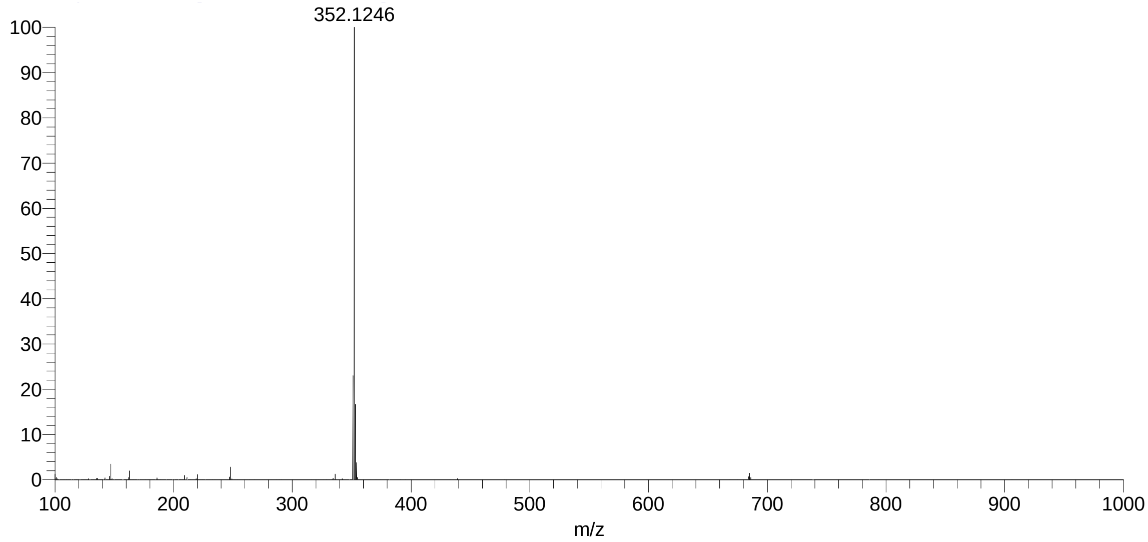

Figure S28. Full ESI$^+$-HRMS spectrum of Cys-Ser conjugate **13** (calculated $C_{13}H_{18}BN_5O_4S$ *m/z* $[M+H]^+$: 352.1245, found 352.1246 from TIC peak at RT 8.82 min).

8.5. Cys-Thr

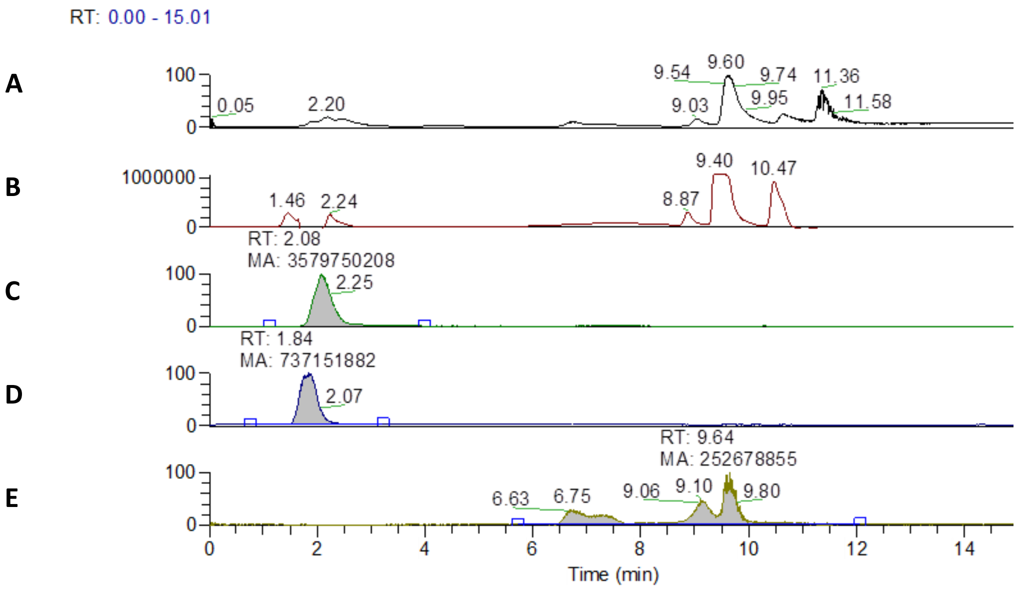

Figure S29. LC-MS chromatograms of Cys-Thr conjugate **14** and Cys-Thr dipeptide. **A** – Reaction mixture TIC after 48 h; **B** – Reaction detection at 210 nm, after 48 h; **C** – Reaction t_0 – unreacted Cys-Thr EIC (base peak *m/z* 222.0907); **D** – Unreacted Cys-Thr EIC after 48 h with 8.0 eq of **1**; **E** – Cys-Thr conjugate **14** EIC (base peak *m/z* 366.1402) after 48 h reaction. Cys-Thr conversion was calculated based on the EIC intensity (AUC) within δ 5 ppm range.

Table S5. Cys-Thr dipeptide conversion in 48 h reaction with cyanamide **1** according to AUC from EIC chromatograms of base peak *m/z* 222.0907, within δ 5 ppm range.

Dipeptide	t_0	48 h	Conversion
Cys-Thr (AUC A.U.)	3579750208	737151882	79 %

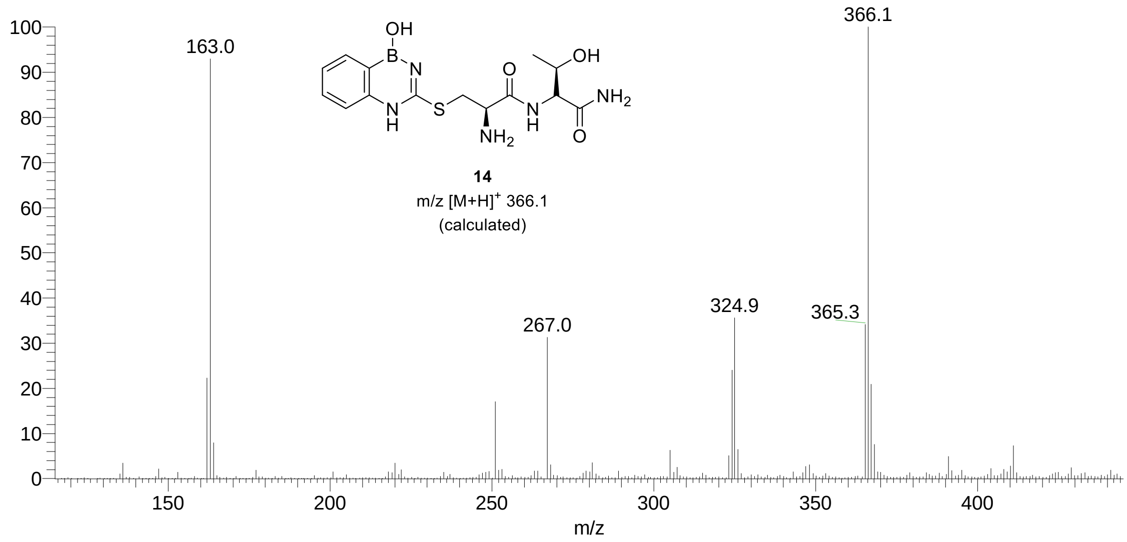

Figure S30. ESI$^+$-LRMS spectrum of reaction between Cys-Thr (calculated $C_7H_{15}N_3O_3S$ *m/z* $[M+H]^+$ 222.1 not found) and cyanamide **1** (calculated $C_7H_7BN_2O_2$ *m/z* $[M\text{-}pin+H]^+$ 163.1, found 163.0), and the resulting product **14** (calculated $C_{14}H_{20}BN_5O_4S$ *m/z* $[M+H]^+$ 366.1, found 366.1).

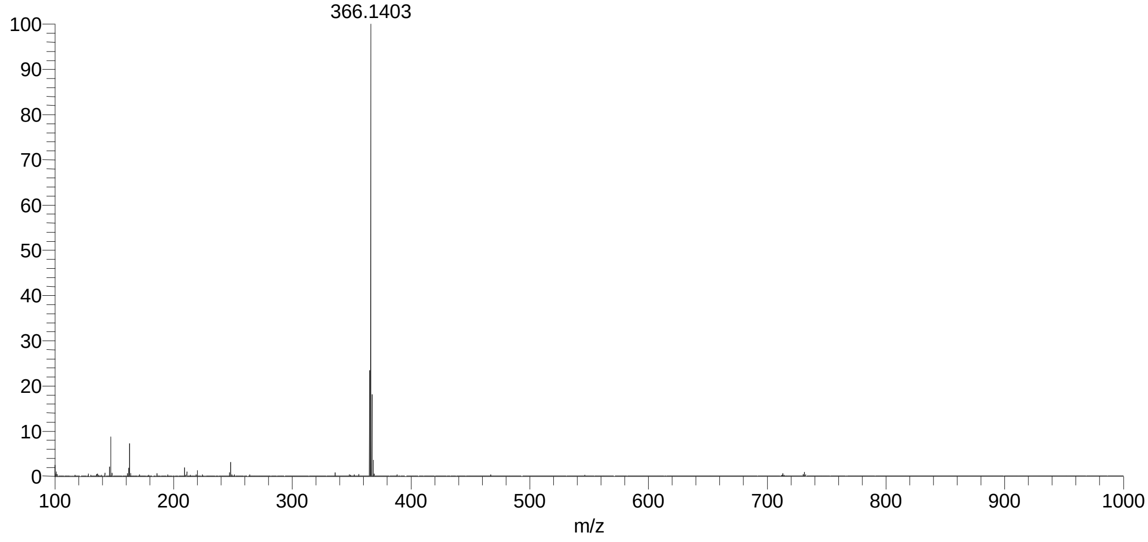

Figure S31. Full ESI$^+$-HRMS spectrum of Cys-Thr conjugate **14** (calculated $C_{14}H_{20}BN_5O_4S$ *m/z* $[M+H]^+$: 366.1402, found 366.1403 from TIC peak at RT 9.64 min).

8.6. Cys-Tyr

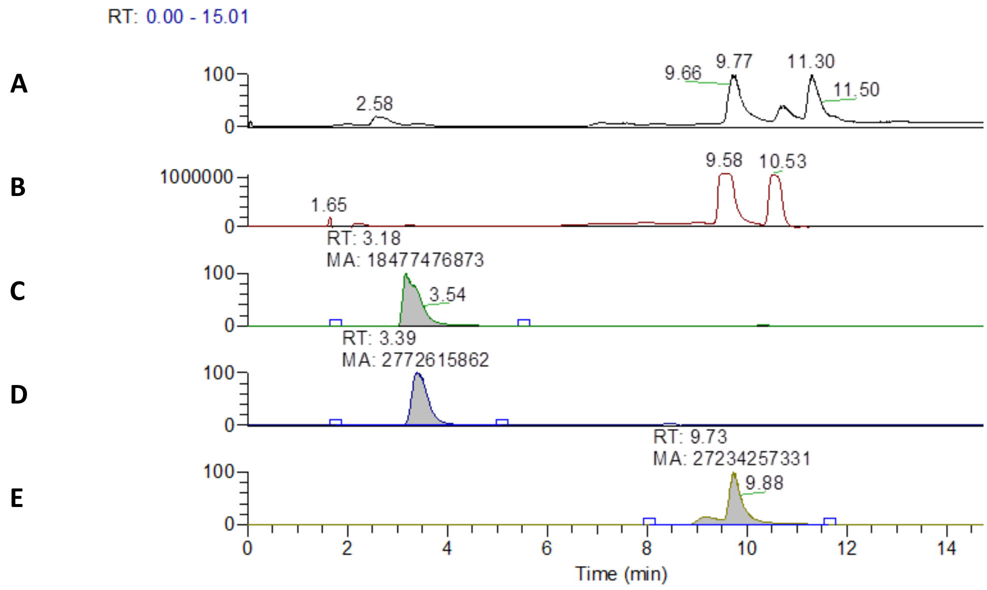

Figure S32. LC-MS chromatograms of Cys-Tyr conjugate **15** and Cys-Tyr dipeptide. **A** – Reaction mixture TIC after 48 h; **B** – Reaction detection at 210 nm, after 48 h; **C** – Reaction t_0 – unreacted Cys-Tyr EIC (base peak *m/z* 284.1063); **D** – Unreacted Cys-Tyr EIC after 48 h with 8.0 eq of **1**; **E** – Cys-Tyr conjugate **15** EIC (base peak *m/z* 428.1558) after 48 h reaction. Cys-Tyr conversion was calculated based on the EIC intensity (AUC) within δ 5 ppm range.

Table S6. Cys-Tyr dipeptide conversion in 48 h reaction with cyanamide **1** according to AUC from EIC chromatograms of base peak *m/z* 284.1063, within δ 5 ppm range.

Dipeptide	t_0	48 h	Conversion
Cys-Tyr (AUC A.U.)	18477476873	2772615862	85 %

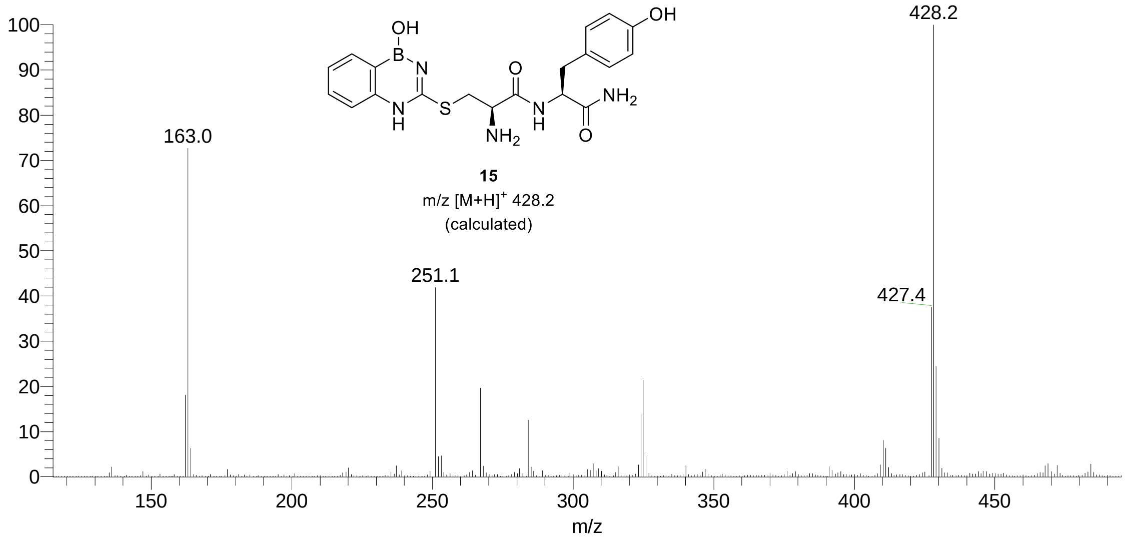

Figure S33. ESI$^+$-LRMS spectrum of reaction between Cys-Tyr (calculated $C_{12}H_{17}N_3O_3S$ *m/z* $[M+H]^+$ 284.1 not found) and cyanamide **1** (calculated $C_7H_7BN_2O_2$ *m/z* $[M\text{-}pin+H]^+$ 163.1, found 163.0), and the resulting product **15** (calculated $C_{19}H_{22}BN_5O_4S$ *m/z* $[M+H]^+$ 428.2, found 428.2).

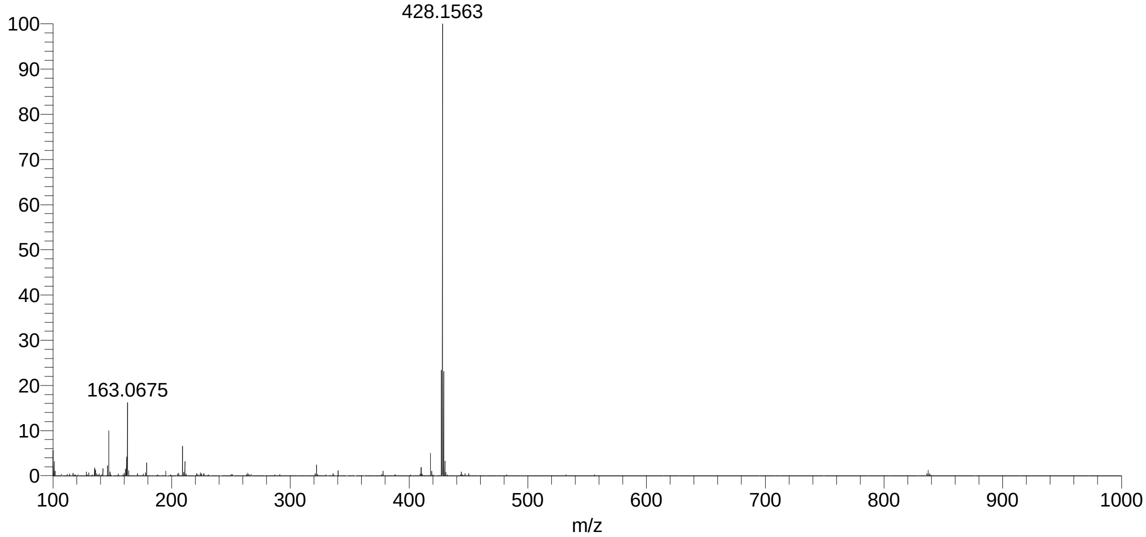

Figure S34. Full ESI$^+$-HRMS spectrum of Cys-Tyr conjugate **15** (calculated $C_{19}H_{22}BN_5O_4S$ *m/z* $[M+H]^+$: 428.1558, found 428.1563 from TIC peak at RT 9.73 min).

8.7. Cys-Glu

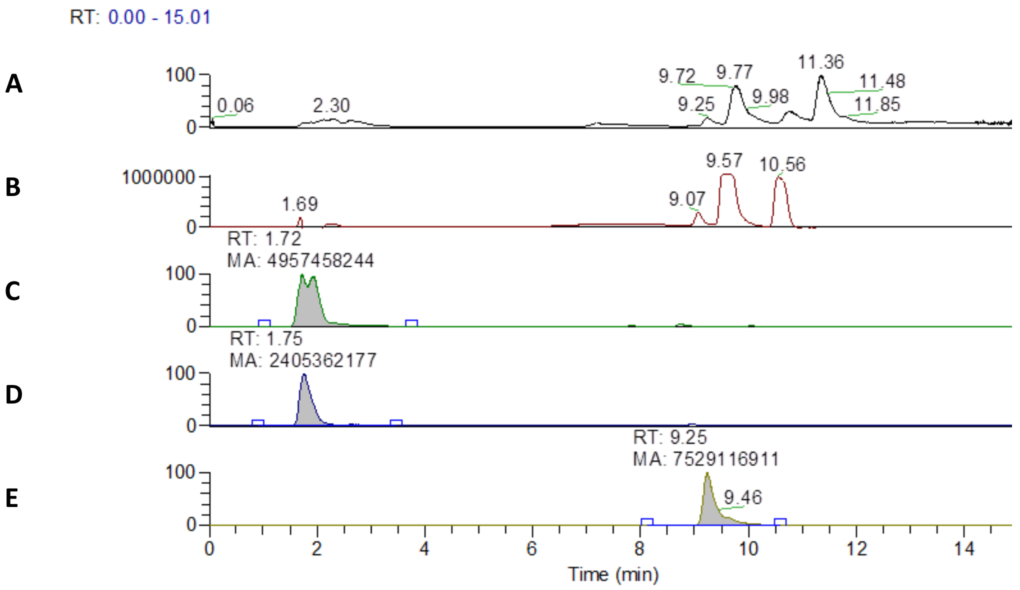

Figure S35. LC-MS chromatograms of Cys-Glu conjugate **16** and Cys-Glu dipeptide. **A** – Reaction mixture TIC after 48 h; **B** – Reaction detection at 210 nm, after 48 h; **C** – Reaction t_0 – unreacted Cys-Glu EIC (base peak *m/z* 250.0856); **D** – Unreacted Cys-Glu EIC after 48 h with 8.0 eq of **1**; **E** – Cys-Glu conjugate **16** EIC (base peak *m/z* 394.1351) after 48 h reaction. Cys-Glu conversion was calculated based on the EIC intensity (AUC) within δ 5 ppm range.

Table S7. Cys-Glu dipeptide conversion in 48 h reaction with cyanamide **1** according to AUC from EIC chromatograms of base peak *m/z* 250.0856, within δ 5 ppm range.

Dipeptide	t_0	48 h	Conversion
Cys-Glu (AUC A.U.)	4957458244	2405362177	51 %

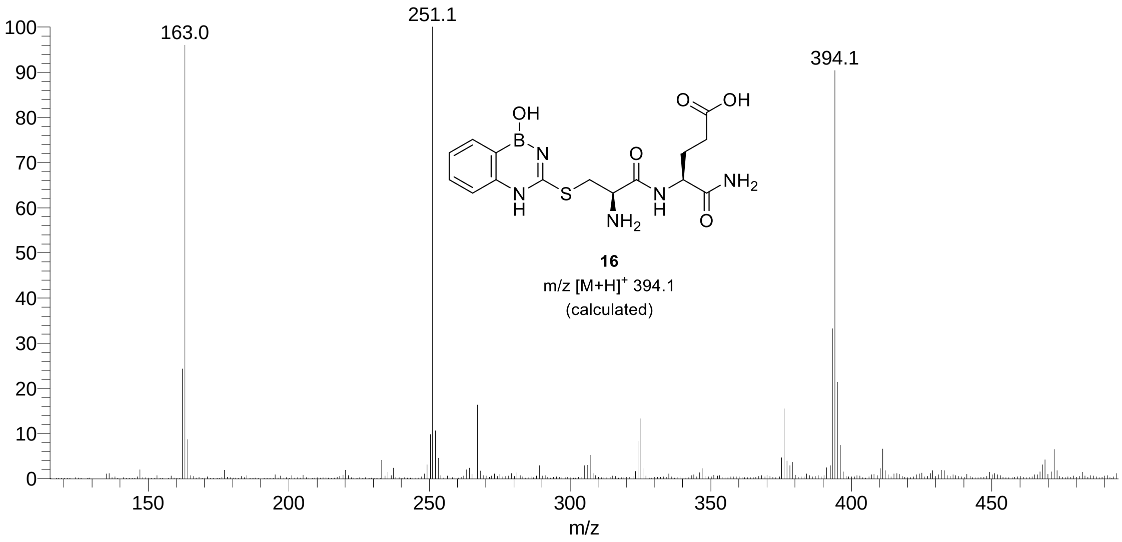

Figure S36. ESI$^+$-LRMS spectrum of reaction between Cys-Glu (calculated $C_8H_{15}N_3O_4S$ *m/z* $[M+H]^+$ 250.1 not found) and cyanamide **1** (calculated $C_7H_7BN_2O_2$ *m/z* $[M\text{-pin}+H]^+$ 163.1, found 163.0), and the resulting product **16** (calculated $C_{15}H_{20}BN_5O_5S$ *m/z* $[M+H]^+$ 394.1, found 394.1).

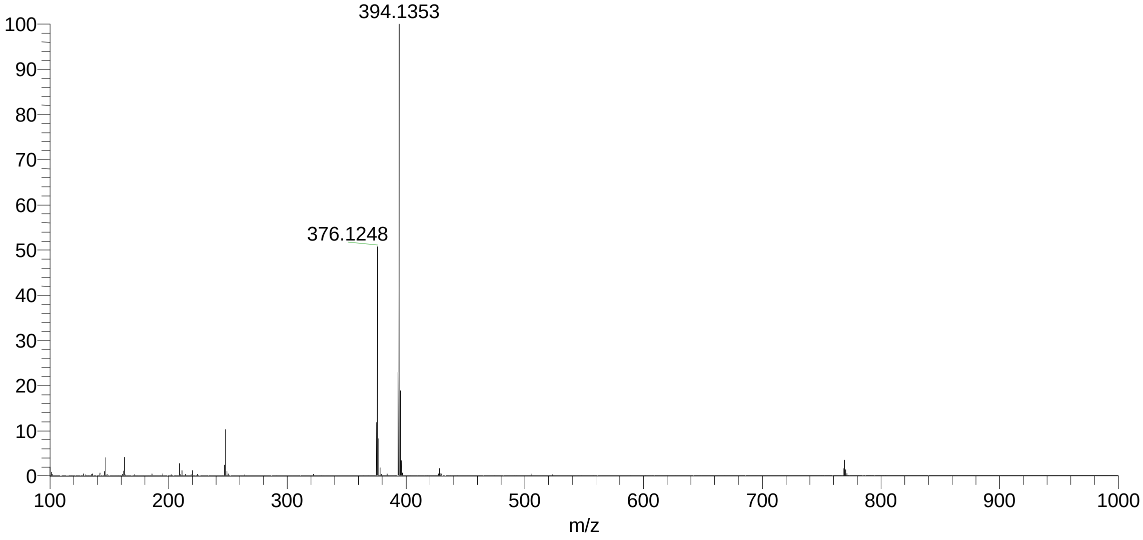

Figure S37. Full ESI$^+$-HRMS spectrum of Cys-Glu conjugate **16** (calculated $C_{15}H_{20}BN_5O_5S$ *m/z* $[M+H]^+$: 394.1351, found 394.1353; $[M\text{-}OH+H]^+$: 376.1245, found 376.1248 from TIC peak at RT 9.25 min).

8.8. Cys-His

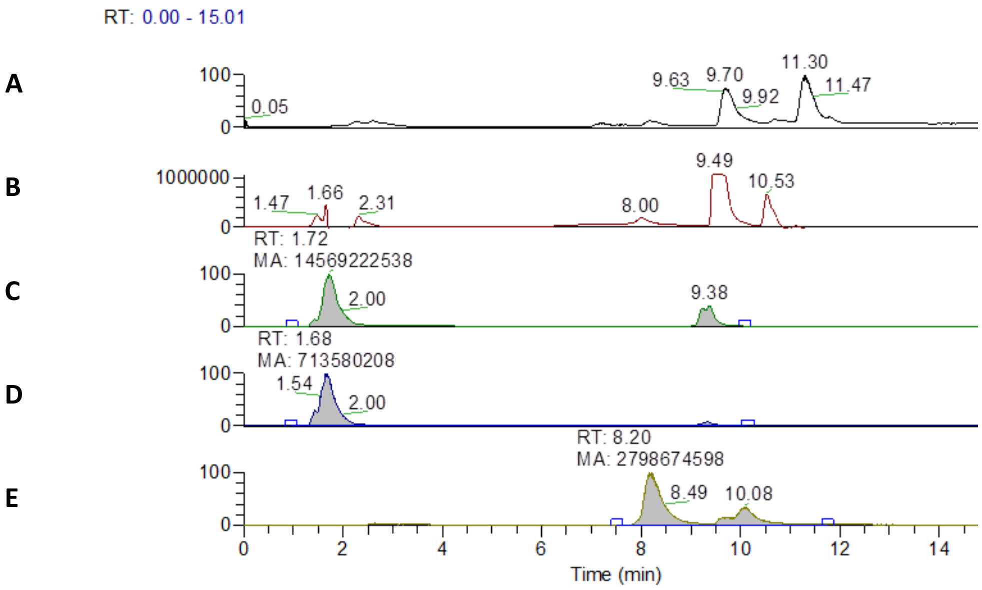

Figure S38. LC-MS chromatograms of Cys-His conjugate **17** and Cys-His dipeptide. **A** – Reaction mixture TIC after 48 h; **B** – Reaction detection at 210 nm, after 48 h; **C** – Reaction t_0 – unreacted Cys-His EIC (base peak *m/z* 258.1019); **D** – Unreacted Cys-His EIC after 48 h with 8.0 eq of **1**; **E** – Cys-His conjugate **17** EIC (base peak *m/z* 402.1514) after 48 h reaction. Cys-His conversion was calculated based on the EIC intensity (AUC) within δ 5 ppm range.

Table S8. Cys-His dipeptide conversion in 48 h reaction with cyanamide **1** according to AUC from EIC chromatograms of base peak *m/z* 258.1019, within δ 5 ppm range.

Dipeptide	t_0	48 h	Conversion
Cys-His (AUC A.U.)	14569222538	713580208	95 %

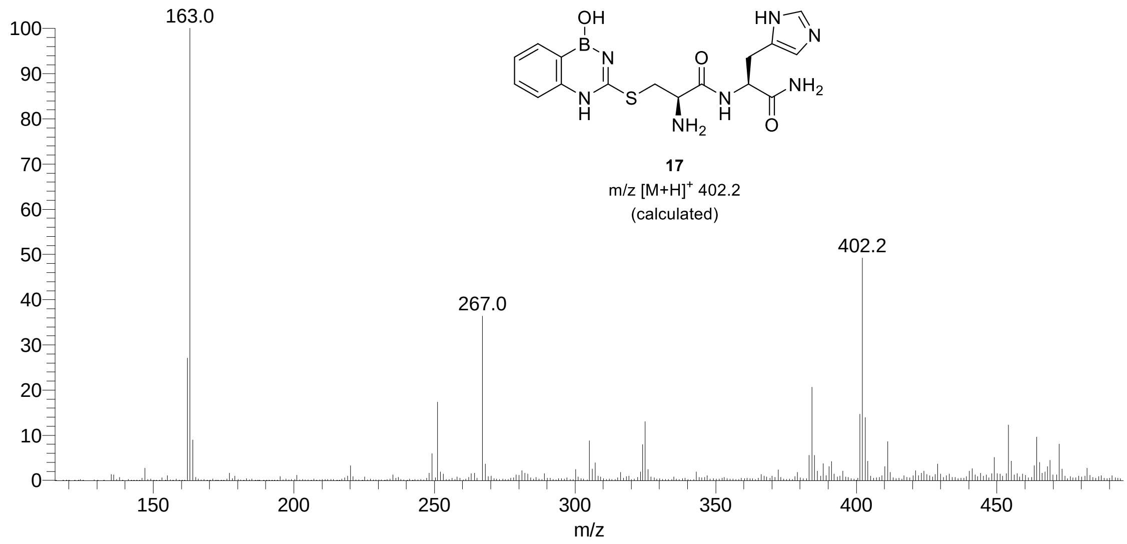

Figure S39. ESI$^+$-LRMS spectrum of reaction between Cys-His (calculated $C_9H_{15}N_5O_2S$ *m/z* [M+H]$^+$ 258.1 not found) and cyanamide **1** (calculated $C_7H_7BN_2O_2$ *m/z* [M-pin+H]$^+$ 163.1, found 163.0), and the resulting product **17** (calculated $C_{16}H_{20}BN_7O_3S$ *m/z* [M+H]$^+$ 402.2, found 402.2).

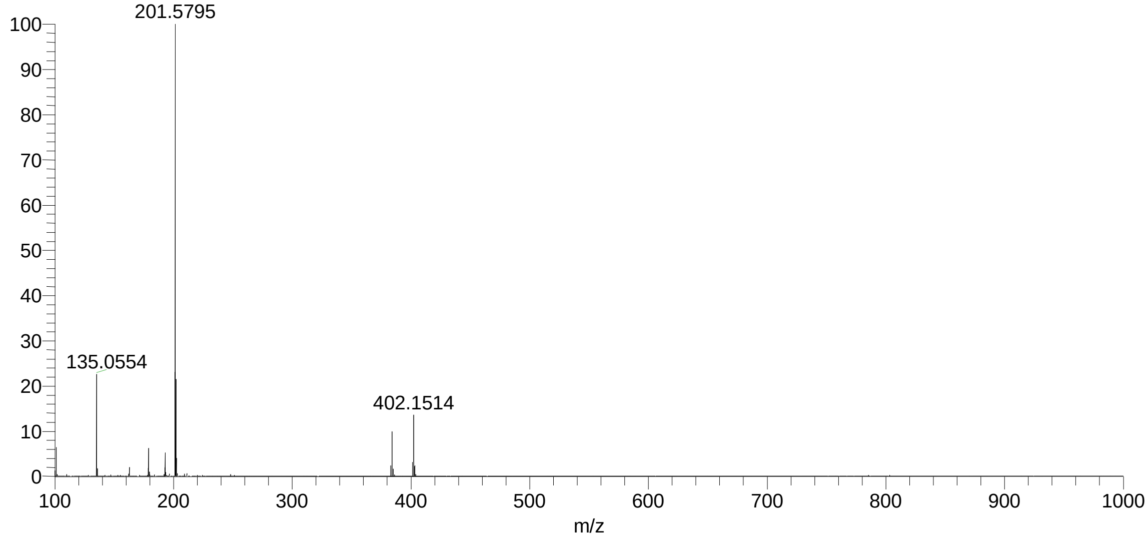

Figure S40. Full ESI$^+$-HRMS spectrum of Cys-His conjugate **17** (calculated $C_{16}H_{20}BN_7O_3S$ *m/z* [M+H]$^+$: 402.1514, found 402.1514; *m/z* [M+2H]$^{2+}$: 201.5793, found 201.5793 from TIC peak at RT 8.20 min).

8.9. Cys-Arg

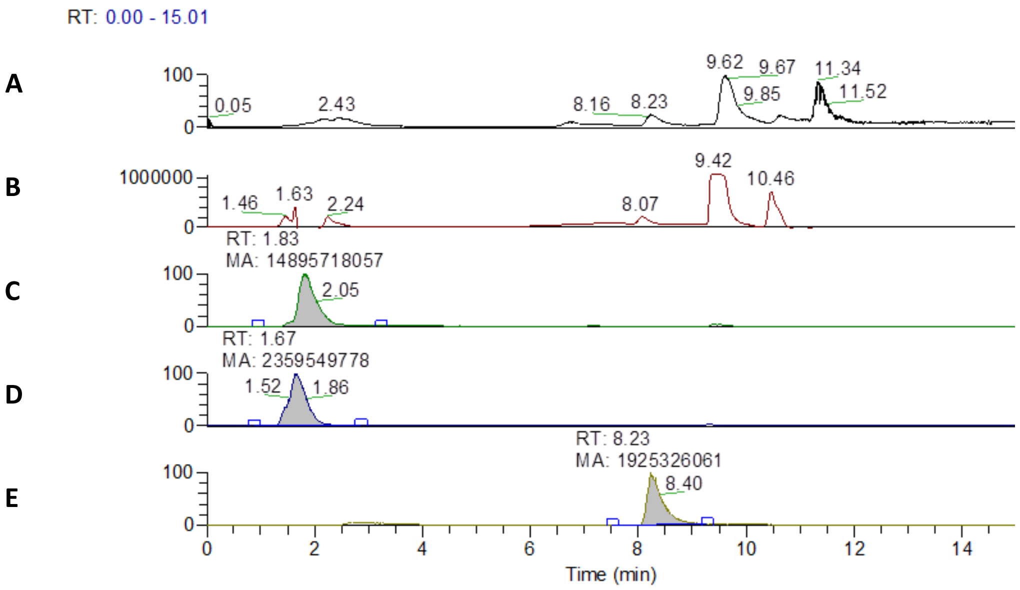

Figure S41. LC-MS chromatograms of Cys-Arg conjugate **18** and Cys-Arg dipeptide. **A** – Reaction mixture TIC after 48 h; **B** – Reaction detection at 210 nm, after 48 h; **C** – Reaction t_0 – unreacted Cys-Arg EIC (base peak *m/z* 277.1441); **D** – Unreacted Cys-Arg EIC after 48 h with 8.0 eq of **1**; **E** – Cys-Arg conjugate **18** EIC (base peak *m/z* 421.1936) after 48 h reaction. Cys-Arg conversion was calculated based on the EIC intensity (AUC) within δ 5 ppm range.

Table S9. Cys-Arg dipeptide conversion in 48 h reaction with cyanamide **1** according to AUC from EIC chromatograms of base peak *m/z* 277.1441, within δ 5 ppm range.

Dipeptide	t_0	48 h	Conversion
Cys-Arg (AUC A.U.)	14895718057	2359549778	84 %

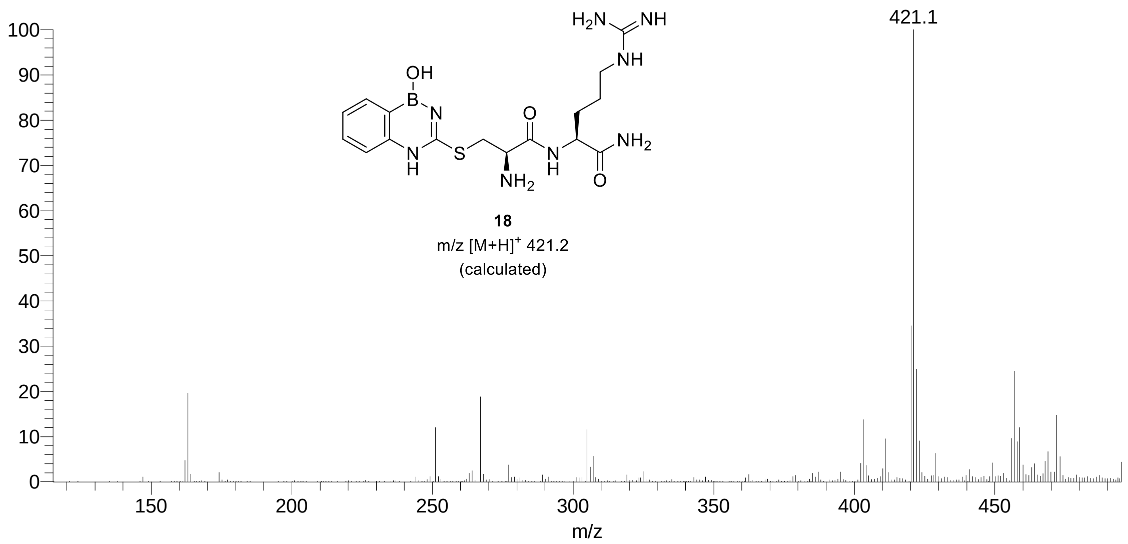

Figure S42. ESI^{+}-LRMS spectrum of reaction between Cys-Arg (calculated $C_9H_{20}N_6O_2S$ *m/z* $[M+H]^+$ 277.1 not found) and cyanamide **1** (calculated $C_7H_7BN_2O_2$ *m/z* $[M\text{-pin}+H]^+$ 163.1, found 163.0), and the resulting product **18** (calculated $C_{16}H_{25}BN_8O_3S$ *m/z* $[M+H]^+$ 421.2, found 421.1).

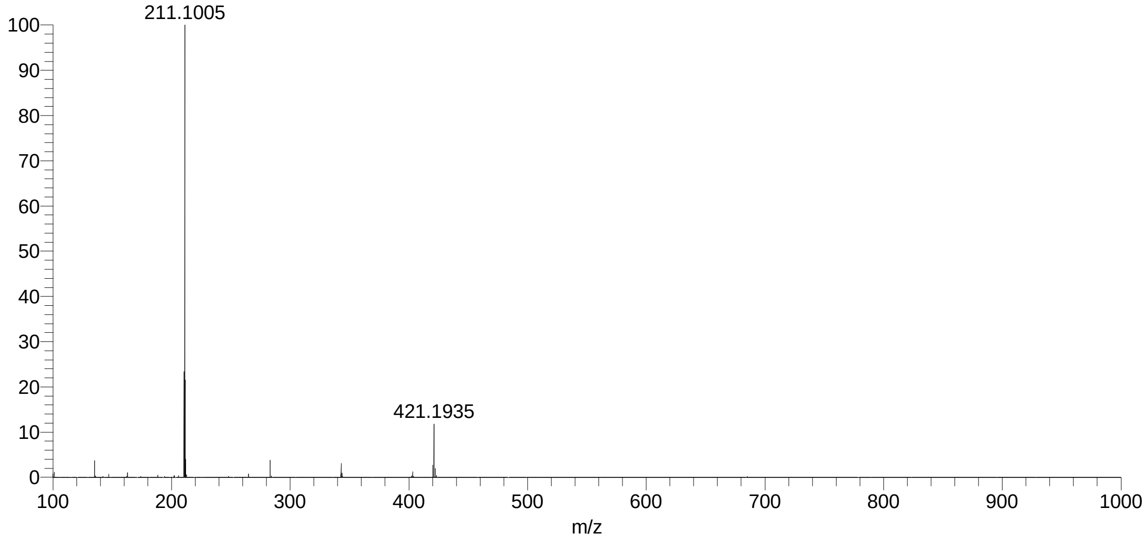

Figure S43. Full ESI^{+}-HRMS spectrum of Cys-Arg conjugate **18** (calculated $C_{16}H_{25}BN_8O_3S$ *m/z* $[M+H]^+$: 421.1936, found 421.1935; *m/z* $[M+2H]^{2+}$: 211.1005 from TIC peak at RT 8.23 min).

8.10. Cys-Lys

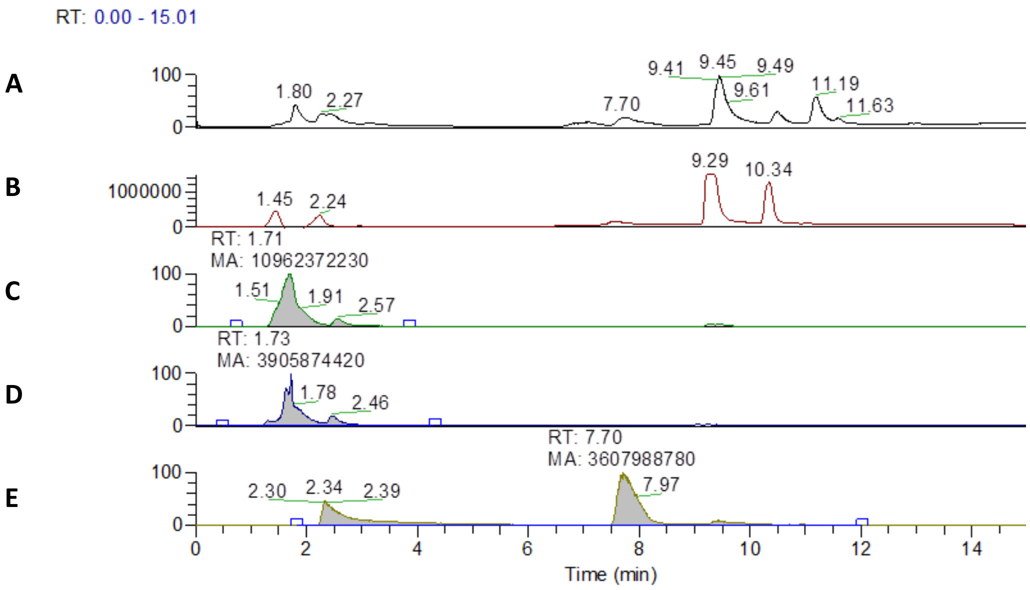

Figure S44. LC-MS chromatograms of Cys-Lys conjugate **19** and Cys-Lys dipeptide. **A** – Reaction mixture TIC after 48 h; **B** – Reaction detection at 210 nm, after 48 h; **C** – Reaction t_0 – unreacted Cys-Lys EIC (base peak *m/z* 249.1380); **D** – Unreacted Cys-Lys EIC after 48 h with 8.0 eq of **1**; **E** – Cys-Lys conjugate **19** EIC (base peak *m/z* 393.1875) after 48 h reaction. Cys-Lys conversion was calculated based on the EIC intensity (AUC) within δ 5 ppm range.

Table S10. Cys-Lys dipeptide conversion in 48 h reaction with cyanamide **1** according to AUC from EIC chromatograms of base peak *m/z* 249.1380, within δ 5 ppm range.

Dipeptide	t_0	48 h	Conversion
Cys-Lys (AUC A.U.)	10962372230	3905874420	64 %

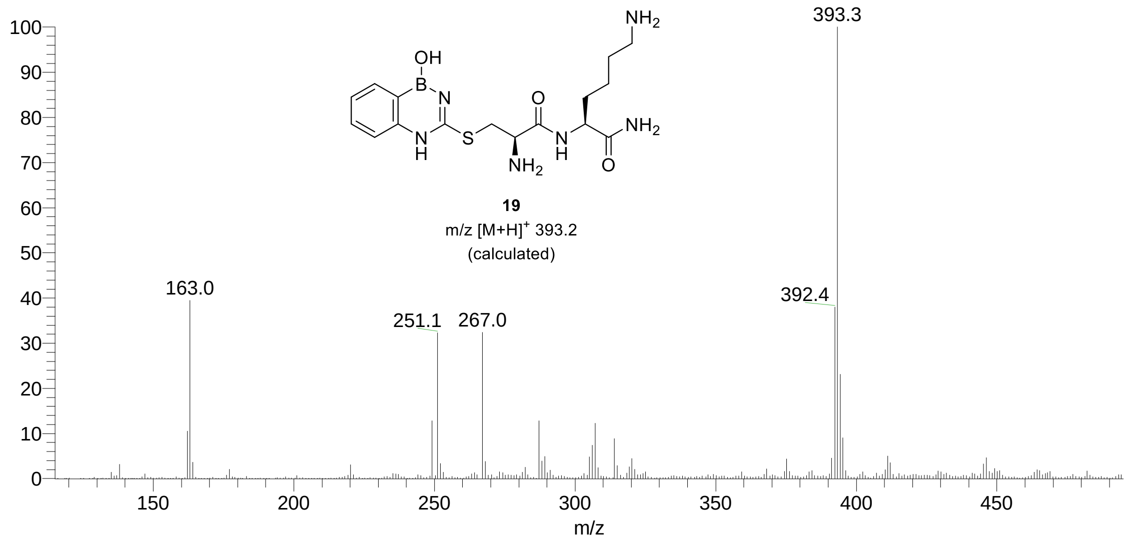

Figure S45. ESI$^+$-LRMS spectrum of reaction between Cys-Lys (calculated $C_9H_{20}N_4O_2S$ *m/z* $[M+H]^+$ 249.1, found 249.0 as a residual peak) and cyanamide **1** (calculated $C_7H_7BN_2O_2$ *m/z* $[M\text{-}pin+H]^+$ 163.1, found 163.0), and the resulting product **19** (calculated $C_{16}H_{25}BN_6O_3S$ *m/z* $[M+H]^+$ 393.2, found 393.3).

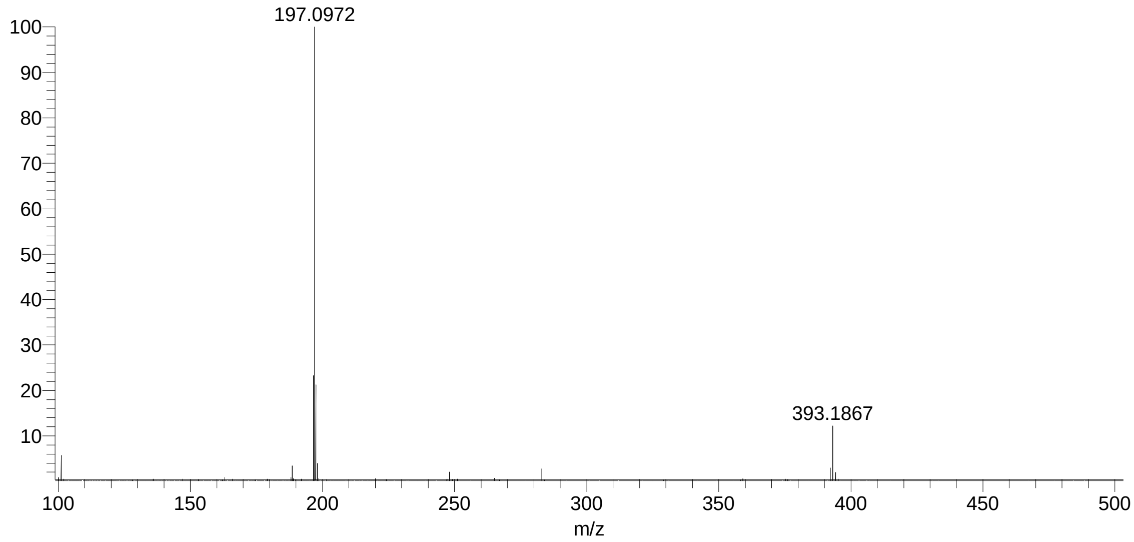

Figure S46. Full ESI$^+$-HRMS spectrum of Cys-Lys conjugate **19** (calculated $C_{16}H_{25}BN_6O_3S$ *m/z* $[M+H]^+$: 393.1875, found 393.1867; *m/z* $[M+2H]^{2+}$: 197.0974, found 197.0972 from TIC peak at RT 7.70 min).

9. ESI-HRMS Assays with Cys-Bombesin and Cyanamide **1**

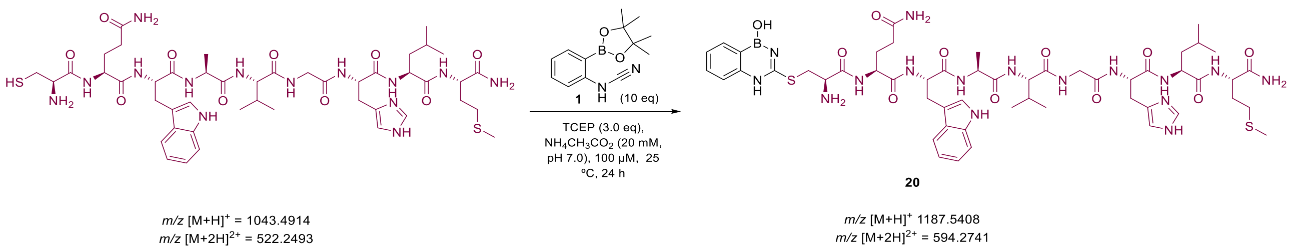

3.0 equivalents of tris-(2-carboxyethyl)phosphine hydrochloride (TCEP, 50 mM, 5 µL, 0.25 µM) were added to a 100 µM solution of Cys-Bombesin (1 mM, 50 µL, 0.05 µM) in ammonium acetate solution 20 mM, pH 7.0 (500 µL) and the mixture reacted at 25 °C for 1 h. Afterwards, 10 equivalents of cyanamide **1** (100 mM, 5.0 µL, 0.50 µM) were added. The reaction was monitored by LC-HRMS in Positive Mode. The HPLC runs were carried out with a gradient of A (Milli Q water containing 0.1 % v/v Formic acid, FA) and B (acetonitrile containing 0.1 % v/v FA, Honeywell HPLC-grade). The mobile phase was t = 0 min, 5 % B; t = 10-15 min, 95 % B; t = 17 min, 5 % B; t = 20 min, stop at a flow rate of 0.2 mL/min, detection EIC.

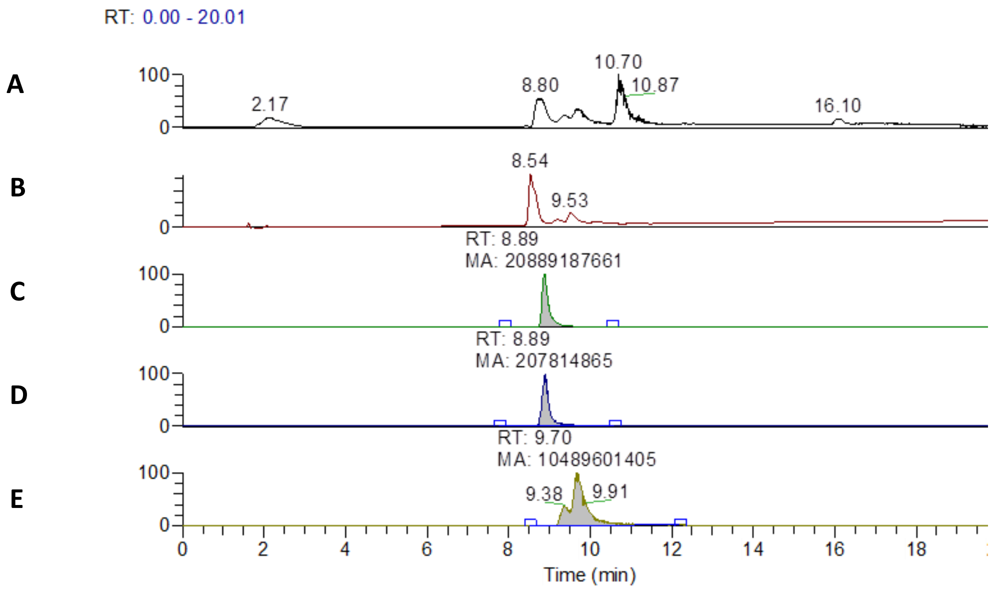

Figure S47. LC-MS chromatograms of Cys-Bombesin conjugate **20** and Cys-Bombesin peptide. **A** – Reaction mixture TIC after 24 h; **B** – Reaction detection at 210 nm, after 24 h; **C** – Reaction t_0 – unreacted Cys-Bombesin EIC (base peak *m/z* 522.2485); **D** – Unreacted Cys-Bombesin EIC after 24 h with 10 equivalents of **1**; **E** – Cys-Bombesin conjugate **20** EIC (base peak *m/z* 594.2741) after 24 h reaction. Cys-Bombesin conversion was calculated based on the EIC intensity (AUC) within δ 5 ppm range.

Table S11. Cys-Bombesin peptide **20** conversion in 24 h reaction with cyanamide **1** according to AUC from EIC chromatograms of base peak *m/z* 522.2485, within δ 5 ppm range.

Peptide	t_0	24 h	Conversion
Cys-Bombesin (AUC A.U.)	20889187661	207814865	99 %

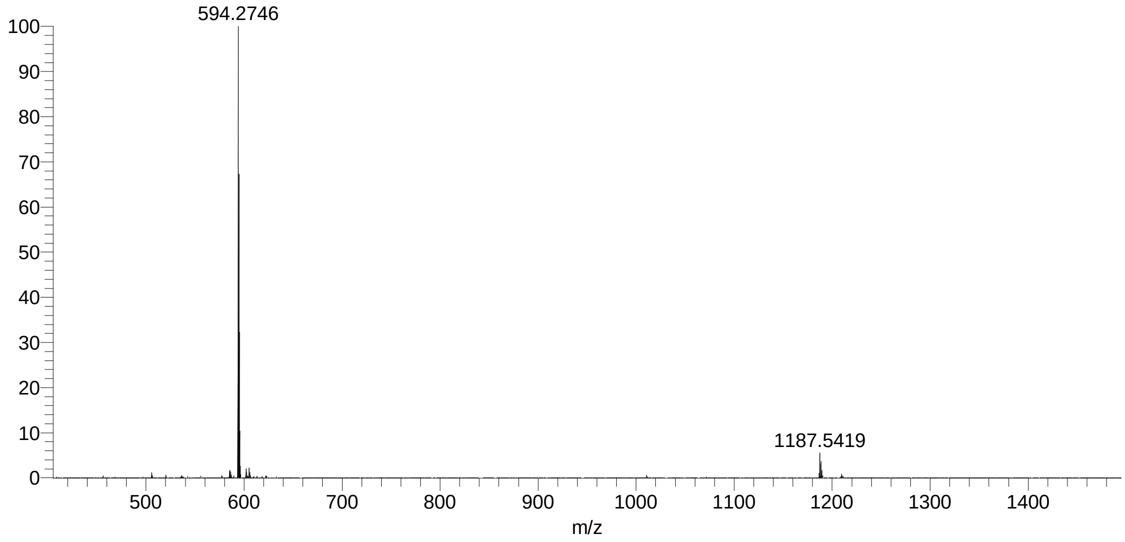

Figure S48. Full ESI$^+$-HRMS spectrum of Cys-Bombesin conjugate **20** (calculated $C_{53}H_{76}BN_{16}O_{11}S_2$ *m/z* $[M+H]^+$: 1187.5408, found 1187.5419; *m/z* $[M+2H]^{2+}$: 594.2741, found 594.2746 from TIC peak at RT 9.70 min).

10. ESI-HRMS Assays with *C*-Ovalbumin and Cyanamide **1**

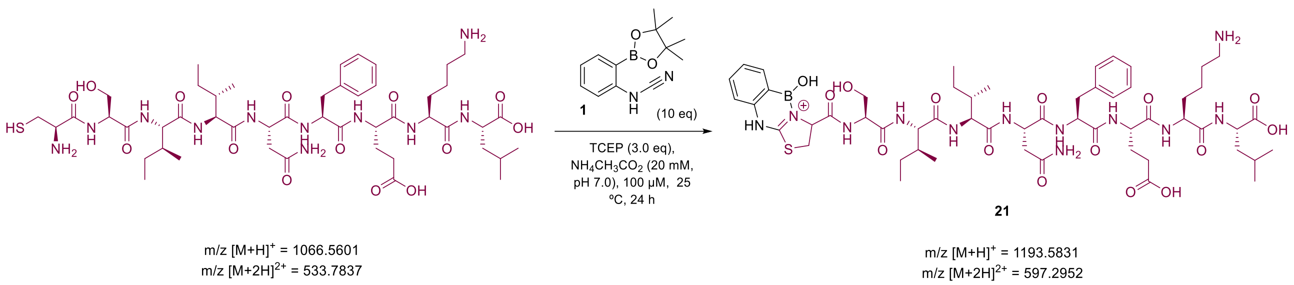

3.0 equivalents of tris-(2-carboxyethyl)phosphine hydrochloride (TCEP, 50 mM, 5 µL, 0.25 µM) were added to a 100 µM solution of *C*-Ovalbumin (1 mM, 50 µL, 0.05 µM) in ammonium acetate solution 20 mM, pH 7.0 (500 µL) and the mixture reacted at 25 °C for 1 h. Afterwards, 10 equivalents of cyanamide **1** (100 mM, 5.0 µL, 0.50 µM) were added. The reaction was monitored by LC-HRMS in Positive Mode. The HPLC runs were carried out with a gradient of A (Milli Q water containing 0.1 % v/v Formic acid, FA) and B (acetonitrile containing 0.1 % v/v FA, Honeywell HPLC-grade). The mobile phase was t = 0 min, 5 % B; t = 10-15 min, 95 % B; t = 17 min, 5 % B; t = 20 min, stop at a flow rate of 0.2 mL/min, detection EIC.

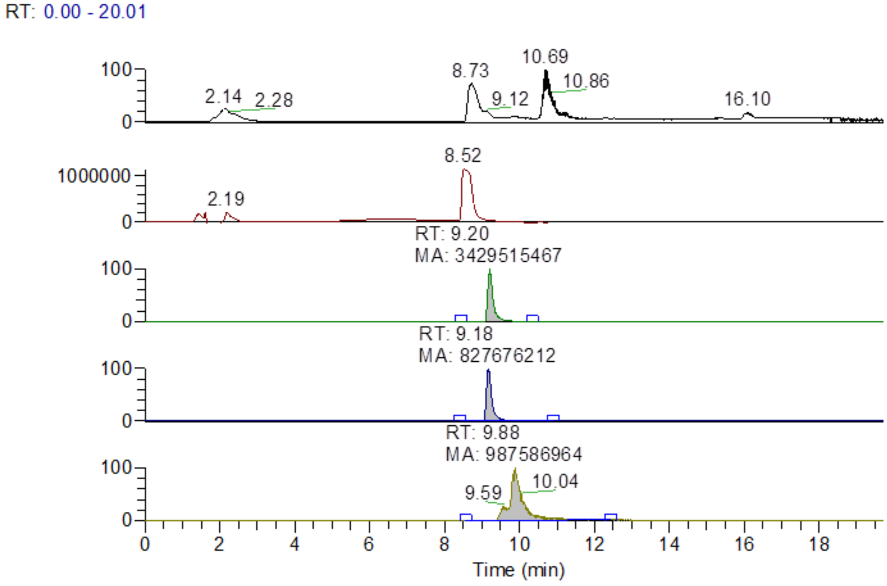

Figure S49. LC-MS chromatograms of *C*-Ovalbumin conjugate **21** and *C*-Ovalbumin peptide. **A** – Reaction mixture TIC after 24 h; **B** – Reaction detection at 210 nm, after 24 h; **C** – Reaction t_0 – unreacted *C*-Ovalbumin EIC (base peak *m/z* 533.7837); **D** – Unreacted *C*-Ovalbumin EIC after 24 h with 10 equivalents of **1**; **E** – *C*-Ovalbumin conjugate **21** EIC (base peak *m/z* 597.2952) after 24 h reaction. *C*-Ovalbumin conversion was calculated based on the EIC intensity (AUC) within δ 5 ppm range.

Table S12. *C*-Ovalbumin peptide conversion in 24 h reaction with cyanamide **1** according to AUC from EIC chromatograms of base peak *m/z* 533.7837, within δ 5 ppm range.

Peptide	t_0	24 h	Conversion
C-Ovalbumin (AUC A.U.)	3429515467	827676212	76 %

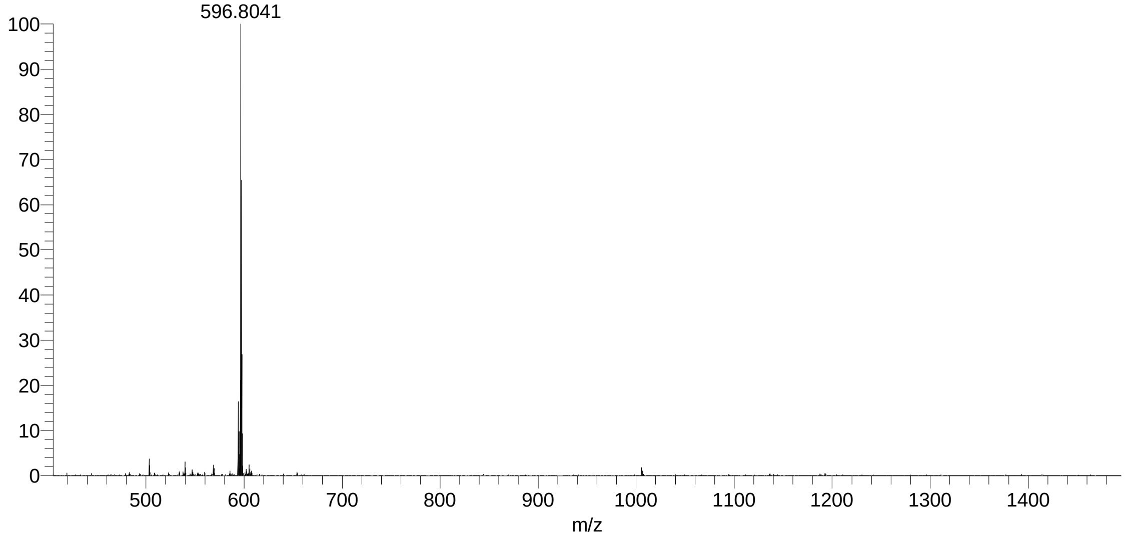

Figure S50. Full ESI$^+$-HRMS spectrum of *C*-Ovalbumin conjugate **21** (calculated $C_{55}H_{83}BN_{12}O_{15}S^+$ *m/z* $[M+2H]^{2+}$: 596.7970, found 596.8041 from TIC peak at RT 9.88 min).

11. ESI-HRMS Assays with CysCys-Bombesin and Cyanamide **1**

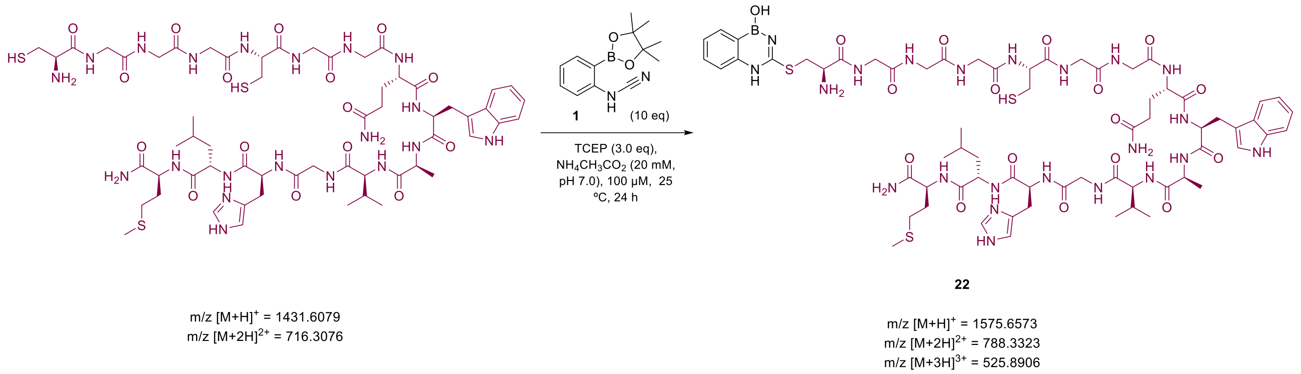

3.0 equivalents of tris-(2-carboxyethyl)phosphine hydrochloride (TCEP, 50 mM, 5 µL, 0.25 µM) were added to a 100 µM solution of CysCys-Bombesin (1 mM, 50 µL, 0.05 µM) in ammonium acetate solution 20 mM, pH 7.0 (500 µL) and the mixture reacted at 25 °C for 1 h. Afterwards, 10 equivalents of cyanamide **1** (100 mM, 5.0 µL, 0.50 µM) were added. The reaction was monitored by LC-HRMS in Positive Mode. The HPLC runs were carried out with a gradient of A (Milli Q water containing 0.1 % v/v Formic acid, FA) and B (acetonitrile containing 0.1 % v/v FA, Honeywell HPLC-grade). The mobile phase was t = 0 min, 5 % B; t = 10-15 min, 95 % B; t = 17 min, 5 % B; t = 20 min, stop at a flow rate of 0.2 mL/min, detection EIC.

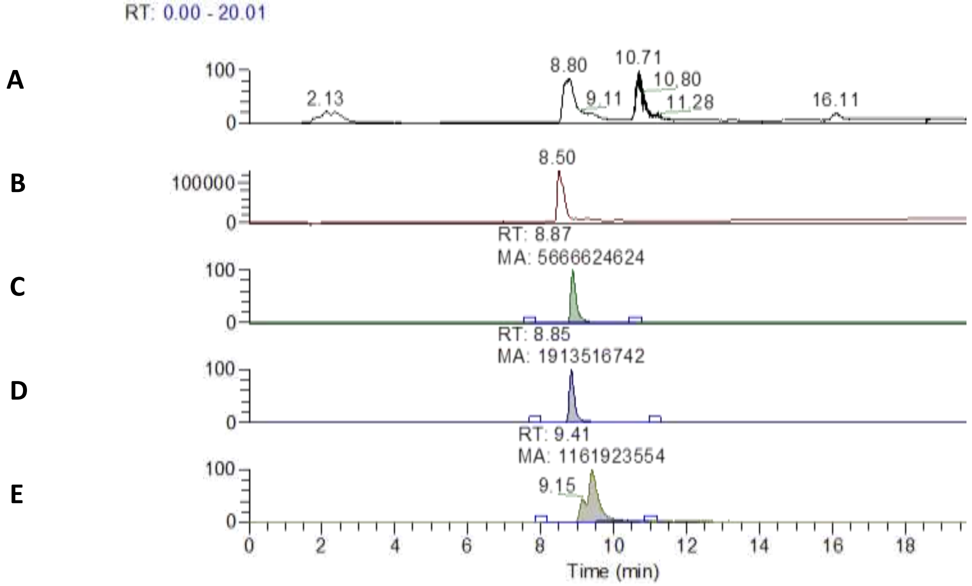

Figure S51. LC-MS chromatograms of CysCys-Bombesin conjugate **22** and CysCys-Bombesin peptide. **A** – Reaction mixture TIC after 24 h; **B** – Reaction detection at 210 nm, after 24 h; **C** – Reaction t_0 – unreacted CysCys-Bombesin EIC (base peak *m/z* 716.3076); **D** – Unreacted CysCys-Bombesin EIC after 24 h with 10 equivalents of **1**; **E** – CysCys-Bombesin conjugate **22** EIC (base peak *m/z* 788.3323) after 24 h reaction. CysCys-Bombesin conversion was calculated based on the EIC intensity (AUC) within δ 5 ppm range.

Table S13. CysCys-Bombesin peptide conversion in 24 h reaction with cyanamide **1** according to AUC from EIC chromatograms of base peak *m/z* 716.3076, within δ 5 ppm range.

Peptide	t_0	24 h	Conversion
CysCys-Bombesin (AUC A.U.)	5666624624	1913516742	66 %

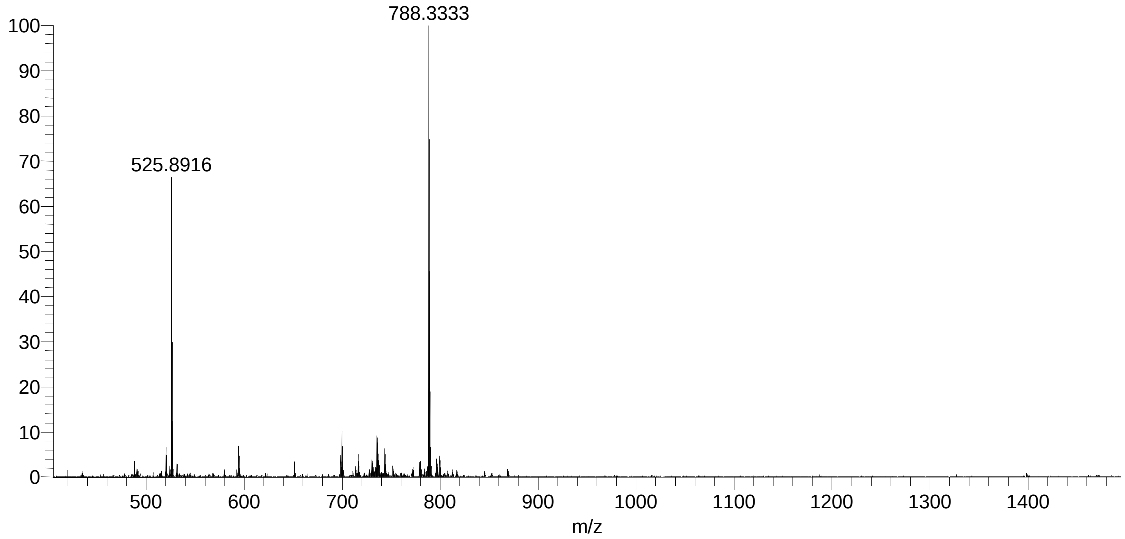

Figure S52. Full ESI$^+$-HRMS spectrum of CysCys-Bombesin conjugate **22** (calculated $C_{66}H_{98}BN_{22}O_{17}S_3$ *m/z* $[M+2H]^{2+}$: 788.3076, found 788.3333; *m/z* $[M+3H]^{3+}$: 525.8906, found 525.8916 from TIC at RT 9.41 min).

12. ESI-HRMS Assays with GV-1001 and Cyanamide **1**

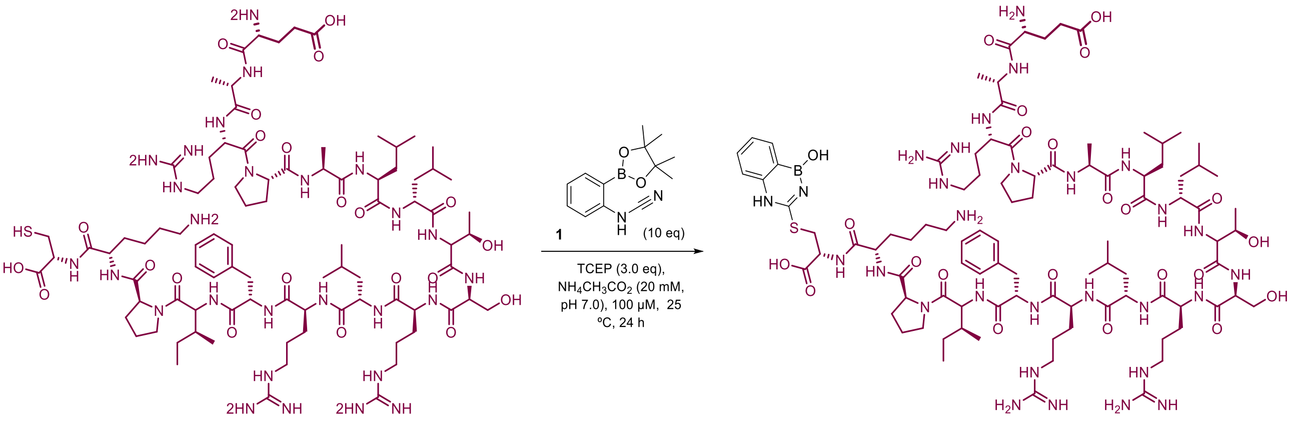

23

m/z $[M+H]^+$ = 1971.1320
m/z $[M+2H]^{2+}$ = 986.0697
m/z $[M+3H]^{3+}$ = 658.0500
m/z $[M+4H]^{4+}$ = 493.7886
m/z $[M+5H]^{5+}$ = 395.0322

m/z $[M+H]^+$ = 2115.1815
m/z $[M+2H]^{2+}$ = 1058.0944
m/z $[M+3H]^{3+}$ = 706.0665
m/z $[M+4H]^{4+}$ = 529.5508
m/z $[M+5H]^{5+}$ = 424.0428

3.0 equivalents of tris-(2-carboxyethyl)phosphine hydrochloride (TCEP, 50 mM, 5 µL, 0.25 µM) were added to a 100 µM solution of GV-1001 (1 mM, 50 µL, 0.05 µM) in ammonium acetate solution 20 mM, pH 7.0 (500 µL) and the mixture reacted at 25 °C for 1 h. Afterwards, 10 equivalents of cyanamide **1** (100 mM, 5.0 µL, 0.50 µM) were added. The reaction was monitored by LC-HRMS in Positive Mode. The HPLC runs were carried out with a gradient of A (Milli Q water containing 0.1 % v/v Formic acid, FA) and B (acetonitrile containing 0.1 % v/v FA, Honeywell HPLC-grade). The mobile phase was t = 0 min, 5 % B; t = 10-15 min, 95 % B; t = 17 min, 5 % B; t = 20 min, stop at a flow rate of 0.2 mL/min, detection EIC.

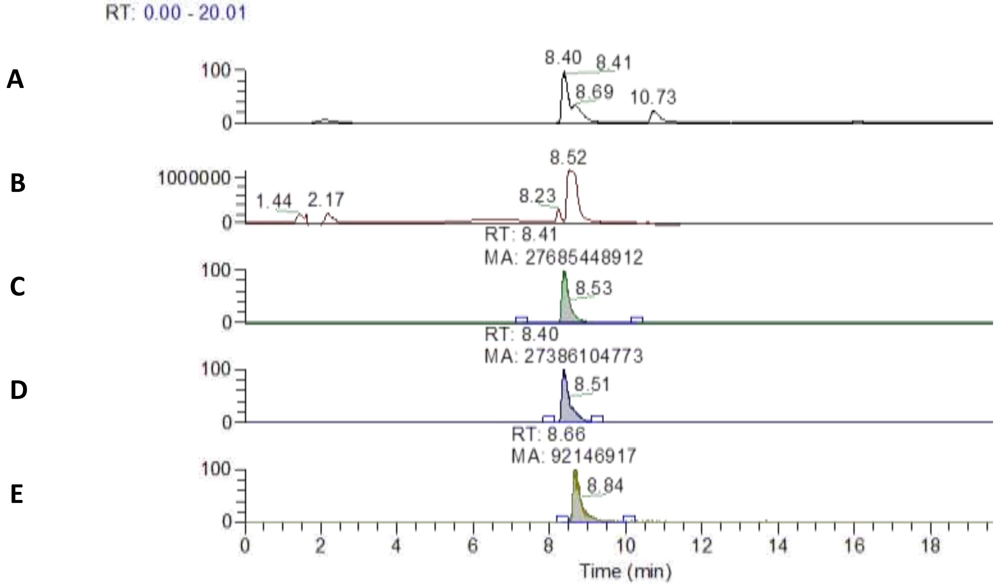

Figure S53. LC-MS chromatograms of GV-1001 conjugate **23** and GV-1001 peptide. **A** – Reaction mixture TIC after 24 h; **B** – Reaction detection at 210 nm, after 24 h; **C** – Reaction t_0 – unreacted GV-1001 EIC (base peak *m/z* 493.7886); **D** – Unreacted GV-1001 EIC after 24 h with 10 equivalents of **1**; **E** – GV-1001 conjugate **23** EIC (base peak *m/z* 706.0665) after 24 h reaction. GV-1001 conversion was calculated based on the EIC intensity (AUC) within δ 5 ppm range.

Table S14. GV-1001 peptide conversion in 24 h reaction with cyanamide **1** according to AUC from EIC chromatograms of base peak *m/z* 493.7886, within δ 5 ppm range.

Peptide	t_0	24 h	Conversion
GV-1001 (AUC A.U.)	27685448912	27386104773	0.1 %

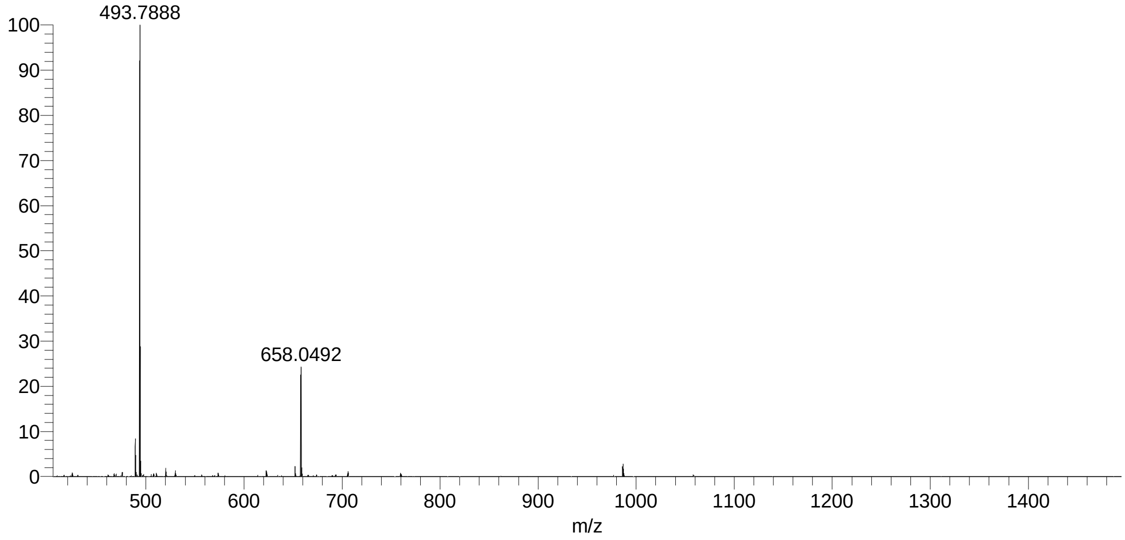

Figure S54. Full ESI$^+$-HRMS spectrum of GV-1001 conjugate **23** (calculated $C_{95}H_{158}BN_{29}O_{23}S$ *m/z* $[M+3H]^{3+}$: 706.0665, found 706.0656 as a residual peak) and GV-1001 peptide (calculated $C_{88}H_{154}BN_{27}O_{22}S$ *m/z* $[M+4H]^{4+}$: 493.7893, found 493.7888; *m/z* $[M+3H]^{3+}$: 657.7155, found 658.0492 from TIC at RT 8.66 min).

13. ESI-MS Assays with CysCys-Bombesin and Cyanamide **8**

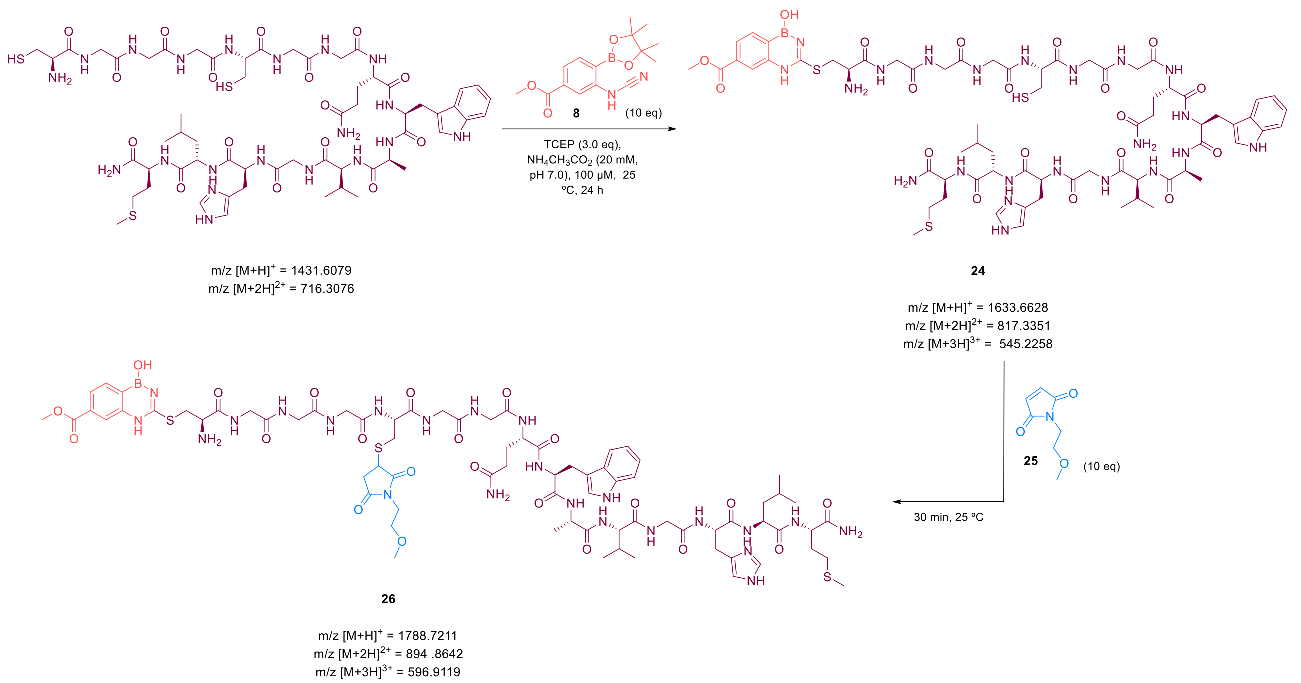

3.0 equivalents of tris-(2-carboxyethyl)phosphine hydrochloride (TCEP, 50 mM, 5 µL, 0.25 µmol) were added to a 100 µM solution of CysCys-Bombesin (1 mM, 50 µL, 0.05 µmol) in ammonium acetate solution 20 mM, pH 7.0 (500 µL) and the mixture reacted at 25 °C for 1 h. Afterwards, 10 equivalents of cyanamide **8** (100 mM, 5.0 µL, 0.50 µmol) were added and the reaction was monitored by ESI-MS and LC-HRMS in Positive Mode. After 24 h, 10 equivalents of 1-(2-methoxyethyl)-1H-pyrrole-2,5-dione (20 mM, 25 µL, 0.5 µmol) were added and the reaction was again followed by ESI-MS and LC-HRMS in Positive Mode. The HPLC runs were carried out with a gradient of A (Milli Q water containing 0.1 % v/v Formic acid, FA) and B (acetonitrile containing 0.1 % v/v FA, Honeywell HPLC-grade). The mobile phase was t = 0 min, 5 % B; t = 10-15 min, 95 % B; t = 17 min, 5 % B; t = 20 min, stop at a flow rate of 0.2 mL/min, detection EIC.

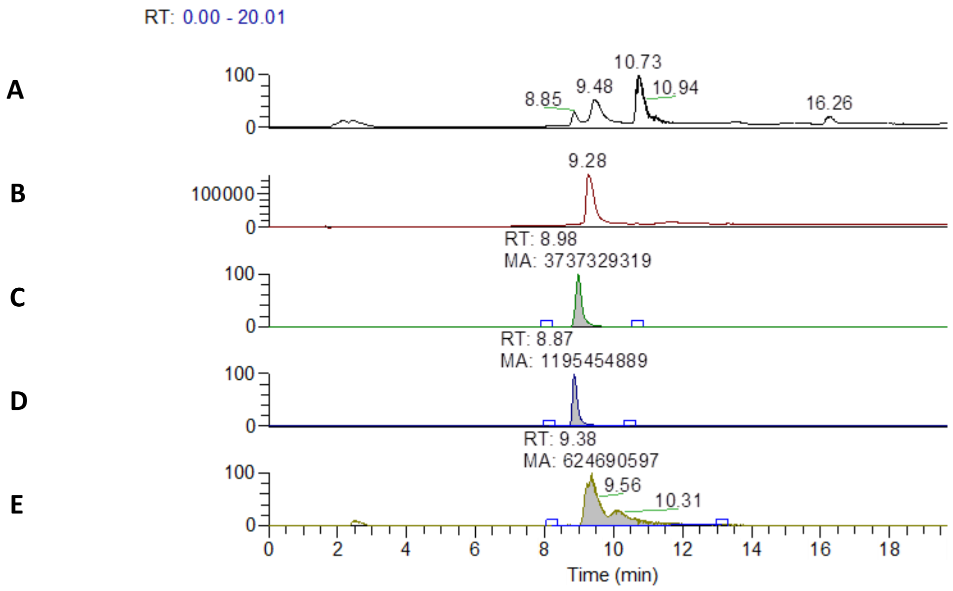

Figure S55. LC-MS chromatograms of CysCys-Bombesin conjugate **24** and CysCys-Bombesin peptide before the addition of maleimide reagent. **A** – Reaction mixture TIC after 24 h; **B** – Reaction detection at 210 nm, after 24 h; **C** – Reaction t_0 – unreacted CysCys-Bombesin EIC (base peak *m/z* 716.3076); **D** – Unreacted CysCys-Bombesin EIC after 24 h with 10 equivalents of **8**; **E** – CysCys-Bombesin conjugate **24** EIC (base peak *m/z* 817.3351) after 24 h reaction. CysCys-Bombesin conversion was calculated based on the EIC intensity (AUC) within δ 5 ppm range.

Table S15. CysCys-Bombesin peptide conversion in 24 h reaction with cyanamide **8** according to AUC from EIC chromatograms of base peak *m/z* 716.3076, within δ 5 ppm range.

Peptide	t_0	24 h	Conversion
CysCys-Bombesin (AUC A.U.)	3737329319	1195454889	68 %

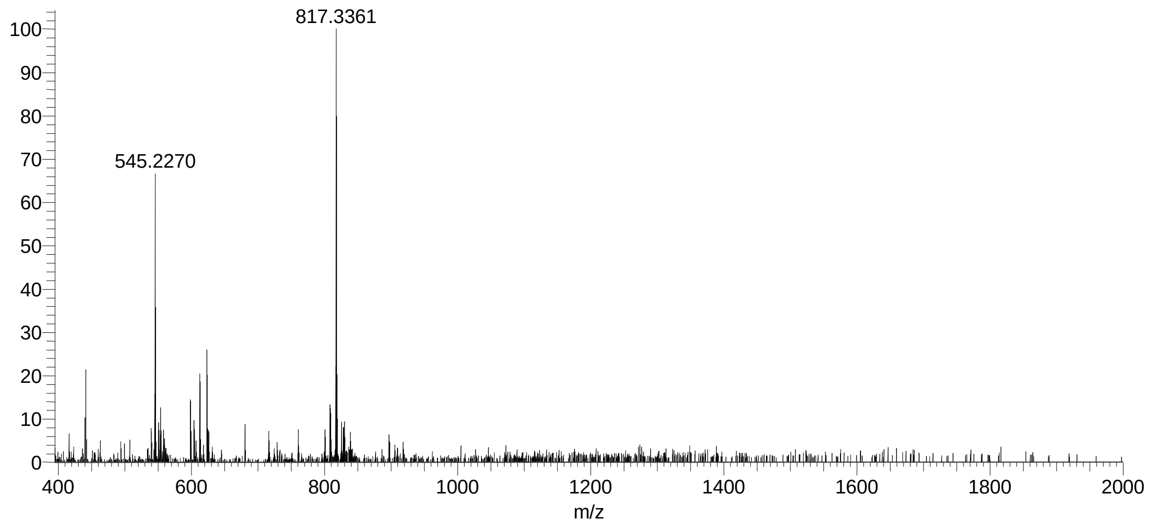

Figure S56. Full ESI$^+$-HRMS spectrum of CysCys-Bombesin conjugate **24** before the addition of maleimide reagent (calculated $C_{68}H_{97}BN_{22}O_{19}S_3$ *m/z* [M+2H]$^{2+}$: 817.3351, found 788.3361; *m/z* [M+3H]$^{3+}$: 545.2258, found 545.2270 from TIC at RT 9.38 min).

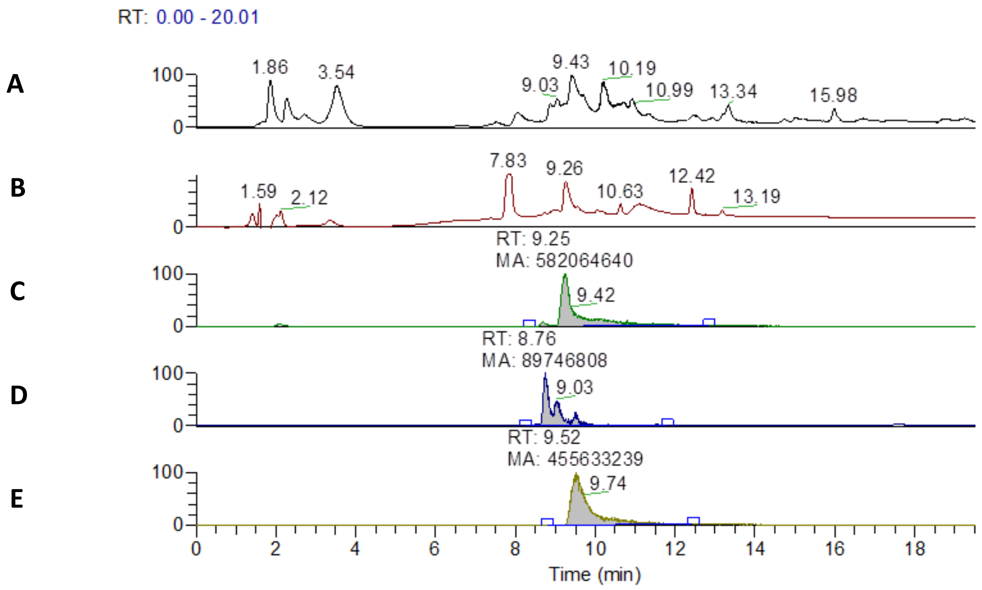

Figure S57. LC-MS chromatograms of CysCys-Bombesin conjugate **26** and CysCys-Bombesin conjugate **24** after the addition of 10 equivalents of maleimide reagent **25**. **A** – Reaction mixture TIC after 30 min; **B** – Reaction detection at 210 nm, after 30 min; **C** – Reaction t_0 - Unreacted conjugate **24** EIC (base peak *m/z* 545.2258); **D** – Unreacted conjugate **24** EIC (base peak *m/z* 545.2258) after 30 min with 10 equivalents of maleimide **25**; **E** - CysCys-Bombesin conjugate **26** EIC (base peak *m/z* 596.9119) after 30 min reaction. Conjugate **24** conversion was calculated based on the EIC intensity (AUC) within δ 5 ppm range.

Table S16. CysCys-Bombesin conjugate **24** conversion in 30 min reaction with maleimide **25** according to AUC from EIC chromatograms of base peak *m/z* 596.9119, within δ 5 ppm range.

Peptide	t_0	30 min	Conversion
CysCys-Bombesin Conjugate 24 (AUC A.U.)	582064640	89746808	85 %

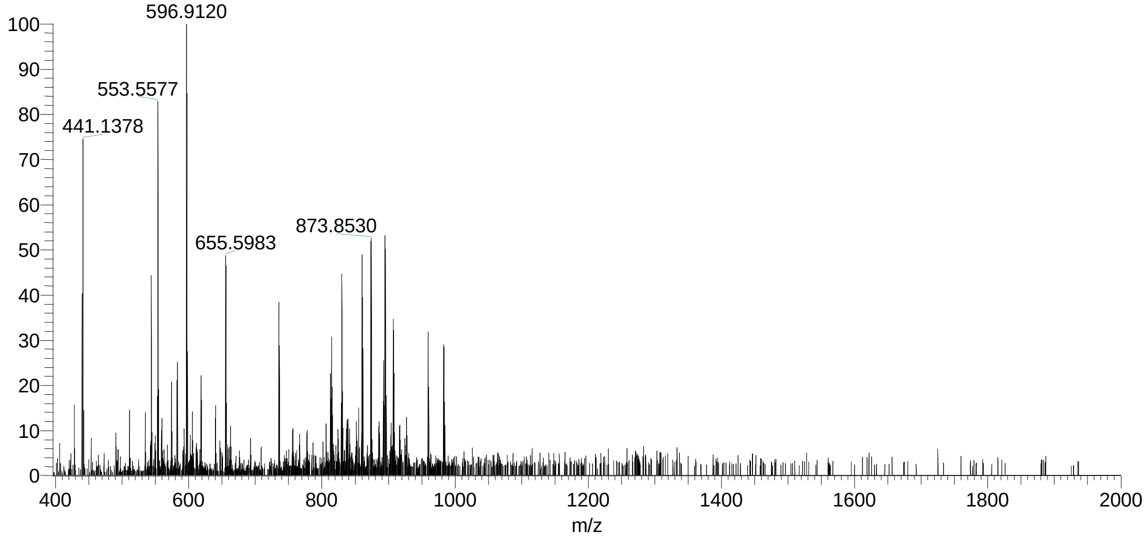

Figure S58. Full ESI$^+$-HRMS spectrum of CysCys-Bombesin conjugate **26** (calculated $C_{75}H_{106}BN_{23}O_{22}S_3$ *m/z* $[M+2H]^{2+}$: 894.8642, found 894.8627; *m/z* $[M+3H]^{3+}$: 596.9119, found 596.9120 from TIC at RT 9.52 min).

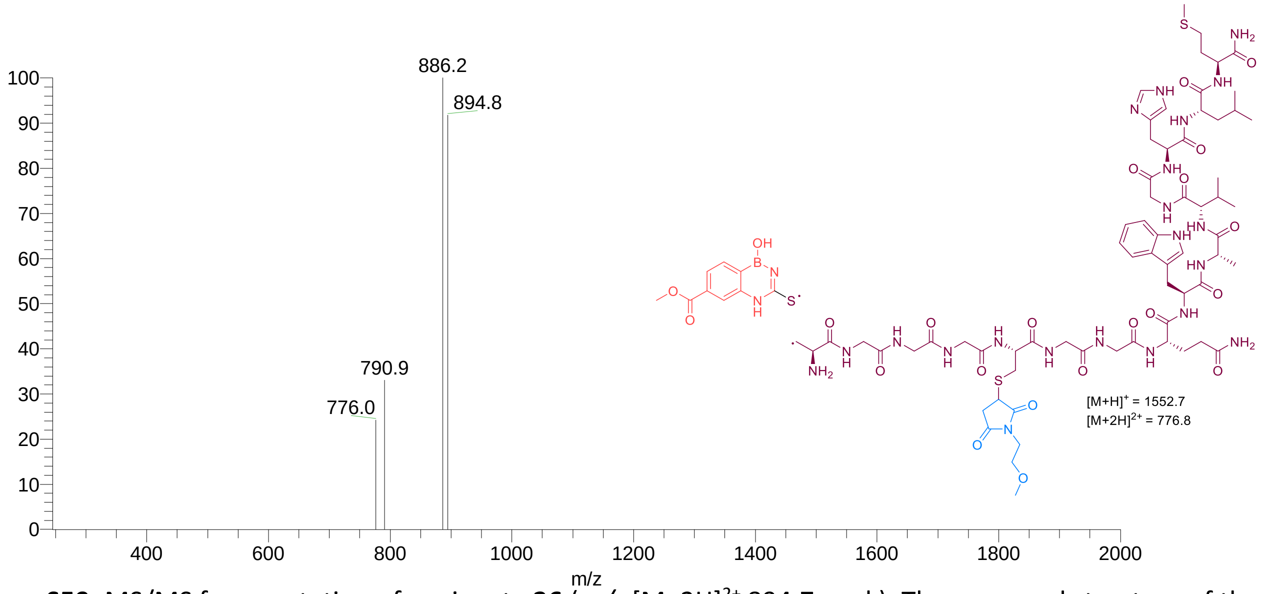

Figure S59. MS/MS fragmentation of conjugate **26** (*m/z* $[M+2H]^{2+}$ 894.7 peak). The proposed structure of the fragment *m/z* 790.1 is coherent with a single cyanamide modification at the *N*-terminal cysteine.

14. Computational Studies

All of the calculations were performed using the Gaussian09 program.[10] Computations were done using wb97xd functional[11] in conjunction with standard basis sets def2SVP and def2TZVP.[12] Geometry full optimizations were made at wb97xd/def2SVP level. Single point calculations using def2TZVP basis set were carried out over optimized geometries to obtain the energy values. Solvent effects (toluene) were considered using the PCM model.[13] The nature of stationary points was defined on the basis of calculations of normal vibrational frequencies (force constant Hessian matrix). The optimizations were carried out using the Berny analytical gradient optimization method.[14] Minimum energy pathways for the reactions studied were found by gradient descent of transition states in the forward and backward direction of the transition vector (IRC analysis).[15] Analytical second derivatives of the energy were calculated to classify the nature of every stationary point, to determine the harmonic vibrational frequencies, and to provide zero-point vibrational energy corrections. The thermal and entropic contributions to the free energies were also obtained from the vibrational frequency calculations, using the unscaled frequencies. Correction to free energy was made by substracing Strans contribution and considering a 1 M concentration.[16] NCI (non-covalent interactions) were computed using the methodology previously described.[17] Data were obtained with the NCIPLOT program.[18] A density cutoff of ρ=0.1 a.u. was applied and the pictures were created for an isosurface value of s=0.5 and colored in the [-0.03,0.03] a.u. sign(λ2)ρ range using VMD software.[19] Structural representations were generated using CYLView.[20]

Models of addition

We studied the reaction of cysteine with substrates **1** and **6**. There are two orientations (**1** and **2** series) of the cyano group as illustrated in **Figure S58**. Consequently, four transition structures were calculated. For the comparison between NH (**a** series) and NMe (**b** series) derivatives the best orientation in each case was selected.

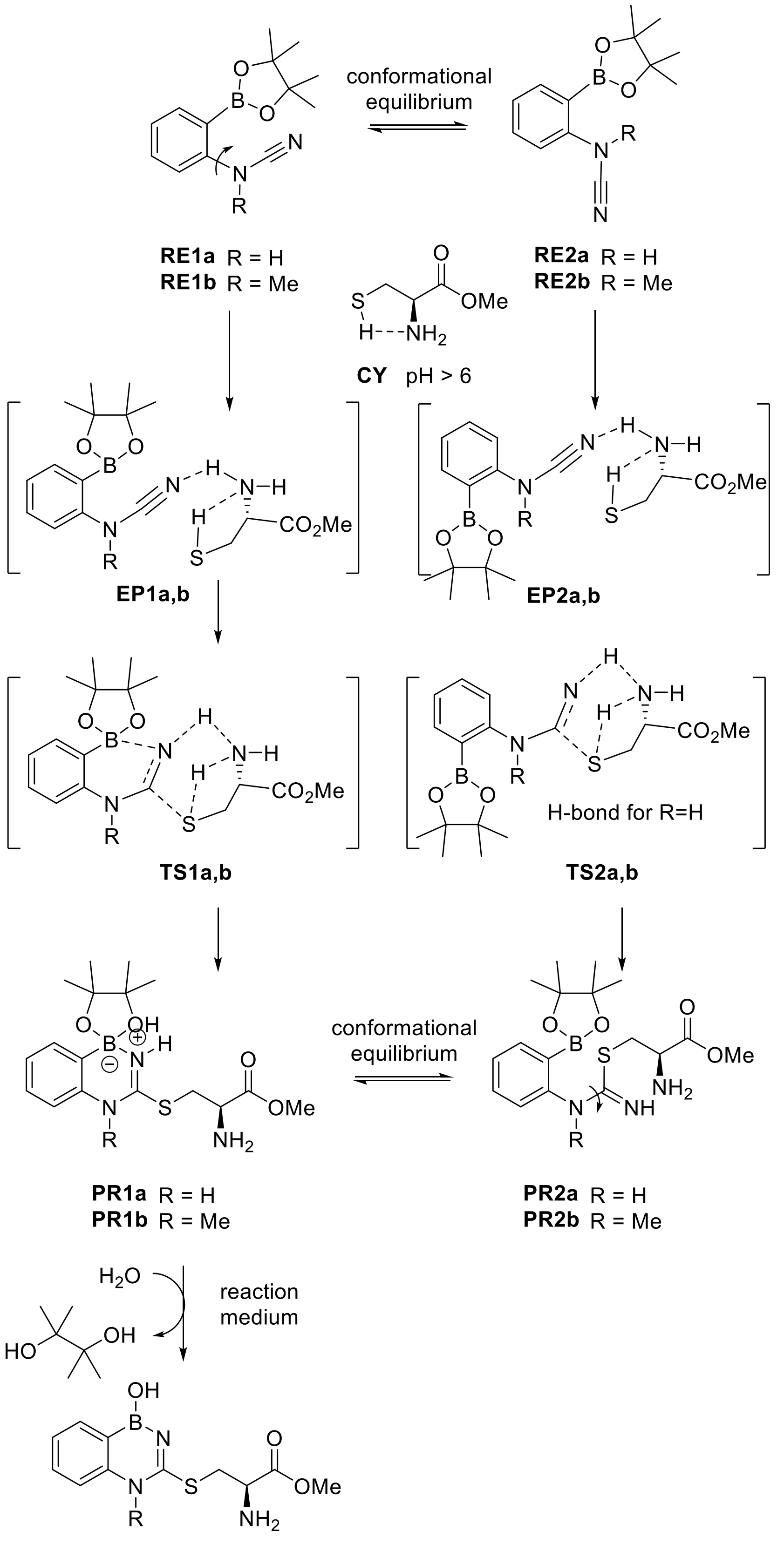

Figure S60. Reaction between *L*-cysteine and compounds **1** (**RE1a/RE2a)** and **6 (RE1b/RE2b)**

Table S17. Calculated (wb97xd/def2TZVP//wb97xd/def2SVP) absolute (hartree) and relative (kcal/mol) energies for the located stationary points corresponding to the addition of CysOMe to **1**

	E(0)	Δ(E0)	Δ G		im. freq
**RE1a**	-1550.039223	3.23	-1550.117200	5.7	
**EP1a**	-1550.023558	13.06	-1550.099279	16.9	
**TS1a**	-1550.001859	26.67	-1550.075364	31.9	-282.5
**PR1a**	-1550.072123	-17.42	-1550.144854	-11.7	
**RE2a**	-1550.044366	0.00[b]	-1550.126242	0.0[b]	
**EP2a**	-1550.028248	10.11	-1550.108178	11.3	
**TS2a**	-1550.011104	**20.87**	-1550.087395	**24.4**	-351.3
**PR2a**	-1550.057406	-8.18	-1550.134874	-5.4	

[a] Correction to free energy was made by subtracting Strans contribution and considering a 1 M concentration. [b] Minimum energy.

Table S18. Calculated (wb97xd/def2TZVP//wb97xd/def2SVP) absolute (hartree) and relative (kcal/mol) energies for the located stationary points corresponding to the addition of CysOMe to **6**

	E(0)	Δ(E0)	Δ G		im. freq
**RE1b**	-1589.274350	0.77	-1589.353409	2.3	
**EP1b**	-1589.274365	0.76	-1589.353432	2.3	
**TS1b**	-1589.234471	25.79	-1589.311620	**28.5**	-217.5
**PR1b**	-1589.307383	-19.96	-1589.382115	-15.7	
**RE2b**	-1589.275572	0.00[b]	-1589.357027	0.0[b]	
**EP2b**	-1589.253114	14.09	-1589.334835	13.9	
**TS2b**	-1589.234961	**25.48**	-1589.310975	28.9	-173.6
**PR2b**	-1589.286319	-6.74	-1589.364801	-4.9	

[a] Correction to free energy was made by subtracting Strans contribution and considering a 1M solution of compound **1**.

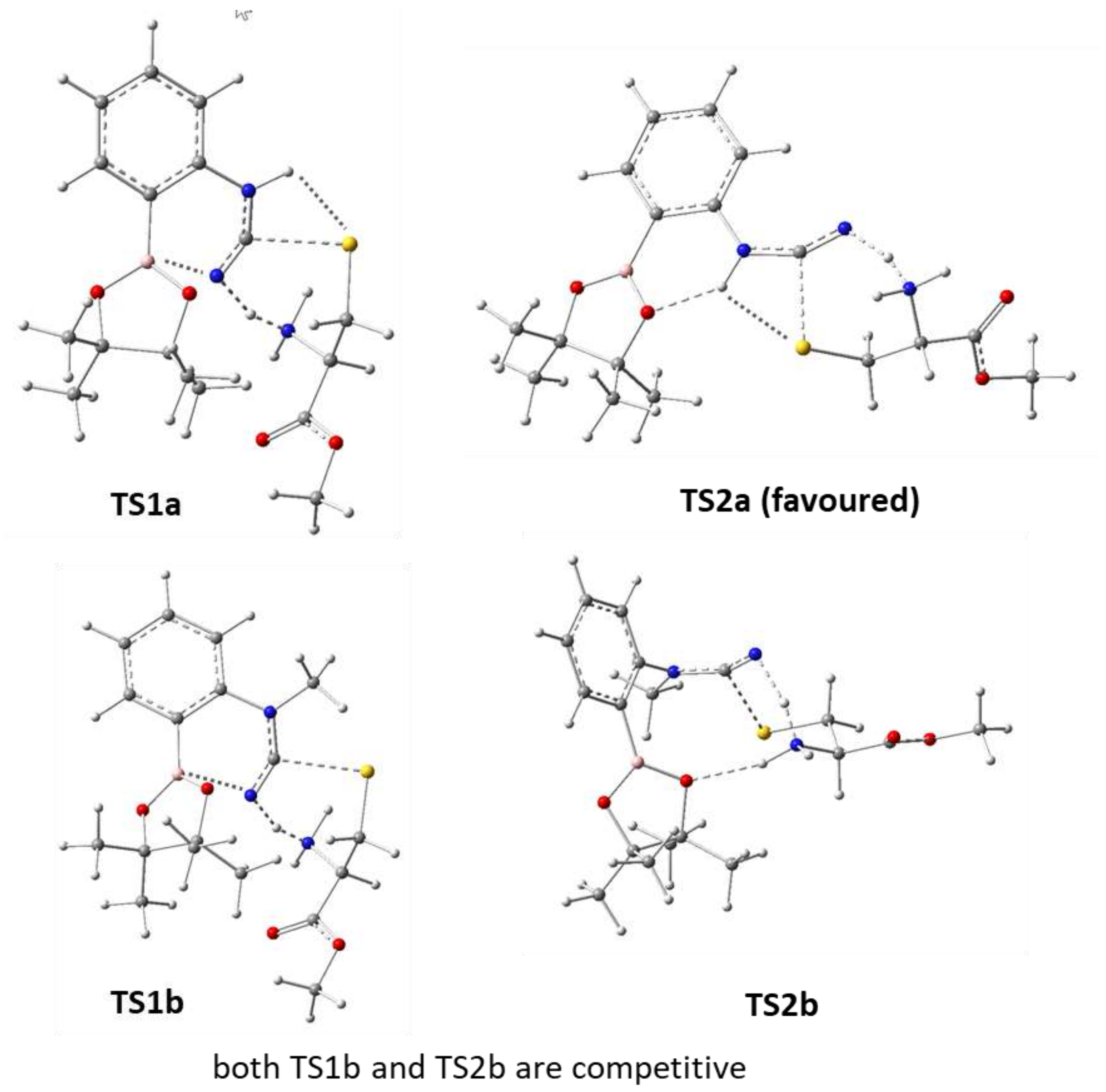

Figure S61. Optimized transition structures

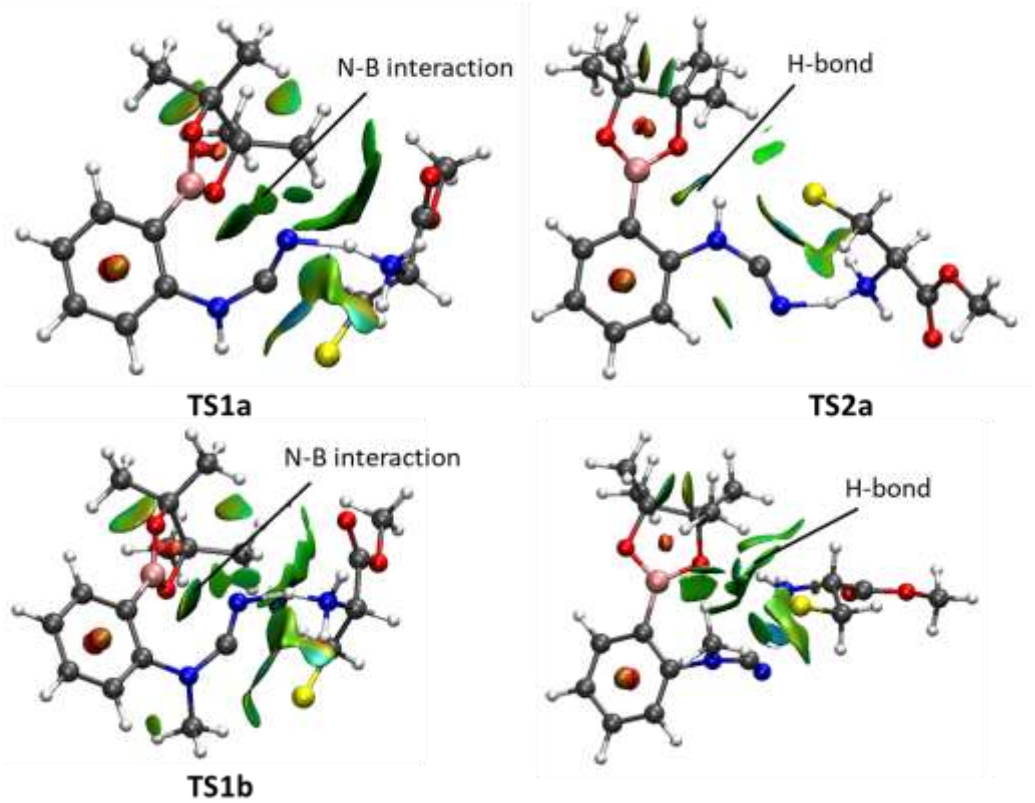

Figure S62. NCI-analysis of the optimized transition structures. Weak non-covalent Van der Waal interactions are represented in green. Higher forces like H-bond are indicated as blue discs. In red unfavorable interactions are represented.

Cartesian coordinates

EP1a

0 1

C	-2.8485193833	-0.2711077929	0.3071449433
C	-2.4509379754	-1.6187809589	0.2391281098
C	-3.3913296448	-2.6337737683	0.0430578111
C	-4.7433626866	-2.3218767624	-0.0605639667
C	-5.1673495326	-0.9986770983	0.0584107440
C	-4.2236421356	0.0075291979	0.2414662550
H	-3.0497798766	-3.6691748499	-0.0097209887
H	-5.4712014666	-3.1215675006	-0.2151074684
H	-6.2293999070	-0.7527885030	-0.0023466859
H	-4.5461657287	1.0493743947	0.3082899847
N	-1.0929692276	-2.0047117733	0.3630781369
C	-0.2551105551	-1.5542894704	1.2705798635
N	0.5761133780	-1.2484000532	2.0289199353
S	1.2042571720	-3.2072627601	-1.3974963515
H	-0.5971914933	-2.5802698431	-0.3680530201
B	-1.8626513320	0.9410319226	0.2742279635
O	-2.2379290519	2.2019833192	0.6435856187
O	-0.5956602365	0.8985124123	-0.2354324108
C	1.9015973266	-1.5194002917	-1.3810295260
H	2.1870823550	-1.1821605527	-2.3871523244
H	1.1847743696	-0.7821147013	-0.9791910360
C	3.1834863084	-1.5287658102	-0.5113695267
H	3.9628669190	-2.0663722308	-1.0744975865
N	2.8920884714	-2.3142548509	0.6981809828
H	3.7162993756	-2.6938868798	1.1615336206
H	2.3250849447	-1.7719531756	1.3798436185
C	3.7263824945	-0.1715477465	-0.1217492494
O	3.9273640676	0.1752344714	1.0134364836
O	3.9705142702	0.5799091863	-1.1889752293
C	4.5145673996	1.8712630038	-0.9410245446
H	5.4974063406	1.7878396191	-0.4560125740
H	4.6112181366	2.3529215370	-1.9195281377
H	3.8462554733	2.4531921825	-0.2913569826
H	2.2192115293	-3.0835728556	0.2447544976
C	0.0093817569	2.1869905898	-0.0320366168
C	-1.2497982835	3.1181954710	0.1425369718
C	-1.7827213426	3.6637320655	-1.1825424304
H	-2.7633783844	4.1279804844	-1.0064642757

H	-1.1108581808	4.4226538610	-1.6084429755
H	-1.9165659526	2.8598693413	-1.9210765386
C	-1.0714869981	4.2506182178	1.1417832946
H	-0.2557625918	4.9193680697	0.8280215266
H	-1.9964335845	4.8418305212	1.1996607243
H	-0.8494298007	3.8701178314	2.1462297074
C	0.8762095835	2.5037260249	-1.2411888832
H	1.3129526940	3.5104947285	-1.1549650521
H	1.6935344609	1.7719989016	-1.3052530491
H	0.3072424127	2.4444179288	-2.1771602471
C	0.8661267975	2.0957965449	1.2282219974
H	0.2567889464	1.8578814594	2.1112864308
H	1.5978099935	1.2848652047	1.1208413459
H	1.4073723753	3.0350047370	1.4139631098

EP1b

0 1

C	-3.0475198281	-0.3679069277	0.1128848024
C	-2.8237058725	-1.7512996237	-0.0236136627
C	-3.8975451277	-2.6442268132	-0.0966326660
C	-5.2057219505	-2.1764321132	-0.0119542393
C	-5.4522743141	-0.8173205991	0.1653716499
C	-4.3783907239	0.0655076315	0.2283973359
H	-3.7117615601	-3.7140360915	-0.2000098704
H	-6.0345976627	-2.8855542994	-0.0676916860
H	-6.4760216992	-0.4470196225	0.2460087415
H	-4.5623106781	1.1356629837	0.3467961506
N	-1.5019549723	-2.2791881075	-0.0974075640
C	-0.5886584965	-1.9217849476	0.7980537731
N	0.2333179629	-1.6540380719	1.5750806714
S	2.5920854788	-3.1321608841	-1.3995390881
B	-1.9531065297	0.7502243819	0.0300987656
O	-2.2243307880	2.0274521718	0.4362187158
O	-0.7078346675	0.6227833431	-0.5125431615
C	2.7309417122	-1.3762807902	-0.9298180581
H	3.0583932931	-0.8347994947	-1.8259783678
H	1.7432695680	-0.9875879650	-0.6366035431
C	3.7403554572	-1.1769537624	0.2049024580
H	4.7119767387	-1.5696392684	-0.1522930400
N	3.3464604682	-1.9165800573	1.3766674635
H	3.9928729690	-1.7453064574	2.1420583540
H	2.4147786400	-1.6330105382	1.6895364109

C	4.0098854059	0.3032785492	0.4891872119
O	4.1605807354	0.7666295883	1.5879637627
O	4.1244090552	1.0284079638	-0.6315382909
C	4.4434734041	2.3999091727	-0.4646079642
H	5.4131071761	2.5162675542	0.0404640909
H	4.4846014823	2.8312534530	-1.4709919583
H	3.6747506290	2.9077337789	0.1353963414
H	2.6625323522	-3.5693541333	-0.1213897662
C	0.0060214418	1.8465944758	-0.2432940704
C	-1.1692599084	2.8722433762	-0.0545816379
C	-1.6550302383	3.4750174303	-1.3728793841
H	-2.6013480241	4.0051964955	-1.1947361553
H	-0.9282157034	4.1908165317	-1.7825185205
H	-1.8386448424	2.6943053856	-2.1254074953
C	-0.8971657258	3.9720622919	0.9592747572
H	-0.0250357035	4.5703040102	0.6550074079
H	-1.7665413879	4.6418214089	1.0233446176
H	-0.7108902005	3.5611567722	1.9589742785
C	0.9313727886	2.1488197153	-1.4107460857
H	1.3791008808	3.1477317165	-1.2940411830
H	1.7475892740	1.4144167757	-1.4425012319
H	0.3978896776	2.1130593238	-2.3688920577
C	0.8105411895	1.6266199334	1.0347994283
H	0.1561916760	1.4462041920	1.8990220870
H	1.4318308174	0.7307892653	0.9215919774
H	1.4635049968	2.4819670342	1.2586424168
C	-1.1986482117	-3.4251693644	-0.9511489266
H	-1.4601332886	-4.3827057123	-0.4714983741
H	-1.7623410104	-3.3230379704	-1.8876947625
H	-0.1241561054	-3.4228106513	-1.1839837988

EP2a

0 1

C	2.2637903807	1.7990192623	0.2934459926
C	1.0002444254	2.2345755761	-0.1589243039
C	0.6339414137	3.5817030051	-0.0502132798
C	1.5179101483	4.4994581163	0.5067654011
C	2.7717451989	4.0934568483	0.9632913544
C	3.1268993246	2.7535158537	0.8524317726
H	-0.3461033523	3.9101386724	-0.4030571736
H	1.2180576968	5.5471176135	0.5835205166
H	3.4624943986	4.8163943834	1.4008735512

H	4.1043568659	2.4177506026	1.2076721882
N	0.0992813259	1.3065762813	-0.7171345472
C	-1.1213273308	1.6271397611	-1.1035696301
N	-2.1990476808	1.9155655388	-1.4332232370
S	-1.2962257292	-1.7465162461	-1.3052822976
H	0.3485487573	0.3075781548	-0.8020403908
B	2.7224775133	0.3144162491	0.2076124240
O	3.8500739618	-0.1825170576	0.7916825792
O	2.0097586625	-0.6401553720	-0.4607638511
C	-2.4926774395	-1.3779084584	0.0154134010
H	-2.5775143366	-2.1899560850	0.7510366079
H	-2.2177503028	-0.4646341086	0.5713311868
C	-3.8951556858	-1.1751674386	-0.6180675490
H	-4.2984433133	-2.1679626393	-0.8701202019
N	-3.7092768401	-0.4351923295	-1.8726109766
H	-4.4581372441	-0.5592488759	-2.5515830666
H	-3.5468035006	0.5767451327	-1.7025863355
C	-4.8798861494	-0.4547978404	0.2773483734
O	-5.4283234881	0.5774268515	-0.0062053058
O	-5.0550902818	-1.1134484426	1.4171666502
C	-5.9360036708	-0.5218931273	2.3631895170
H	-6.9460269130	-0.4190055886	1.9418706015
H	-5.9507214580	-1.1938330311	3.2276324116
H	-5.5740265936	0.4727786342	2.6587602347
H	-2.7045161718	-0.8655794748	-2.1765916978
C	2.5503179835	-1.9281543135	-0.1042623226
C	4.0129686098	-1.5395912473	0.3313782196
C	4.9931084685	-1.4988523142	-0.8402547869
H	5.9280723005	-1.0264717555	-0.5069273511
H	5.2241390192	-2.5089103534	-1.2074001642
H	4.5916567338	-0.9076289888	-1.6760390660
C	4.5788426947	-2.3802941302	1.4645193543
H	4.6219943921	-3.4404269032	1.1727098842
H	5.5993668816	-2.0471258018	1.7015543900
H	3.9721913698	-2.2899560472	2.3740640817
C	2.4547250789	-2.8548360416	-1.3046537101
H	2.9518954674	-3.8130685096	-1.0896541585
H	1.3949835112	-3.0492757636	-1.5233304025
H	2.9143159465	-2.4101085818	-2.1963409613
C	1.6952339660	-2.4647114298	1.0422340114
H	1.7549735986	-1.8123879884	1.9263812302
H	0.6510479848	-2.4975928765	0.6982622068
H	2.0063064020	-3.4782473748	1.3334496251

EP2b

0 1

C	-2.8258627566	-0.9932414192	-0.5939007942
C	-2.1234154968	-2.1429784523	-0.1945198025
C	-2.5769370037	-3.4204602024	-0.5204170625
C	-3.7486522032	-3.5755606662	-1.2581368076
C	-4.4727976259	-2.4524290463	-1.6520647602
C	-4.0143315902	-1.1805127025	-1.3164907317
H	-2.0026612823	-4.2887028163	-0.1912202989
H	-4.0985400862	-4.5770406456	-1.5173571189
H	-5.3965364251	-2.5679702852	-2.2228182630
H	-4.5818634101	-0.2996731423	-1.6253058046
N	-0.9223874978	-2.0220260581	0.5846361067
C	0.2350740253	-2.1718020797	-0.0229400273
N	1.2531221973	-2.2928547291	-0.5799318272
S	1.9736776319	0.4831626011	2.3469648848
B	-2.3719448698	0.4778775927	-0.2940611402
O	-3.2648173313	1.5084180652	-0.3646332366
O	-1.1134580543	0.8889594574	0.0435712437
C	3.4630565835	-0.1140575273	1.4921309562
H	4.3899314417	0.1644501060	2.0128125136
H	3.4580554817	-1.2131105620	1.3894434579
C	3.5088606249	0.5156947834	0.0806415122
H	3.7428212501	1.5856755983	0.2001233799
N	2.1546968153	0.4064148707	-0.4753577664
H	1.9365486783	1.0722420027	-1.2136648784
H	1.9592882400	-0.5628139284	-0.8020549562
C	4.5127746354	-0.0946623476	-0.8739716876
O	4.2441213016	-0.5053414051	-1.9725470161
O	5.7321769467	-0.1014111714	-0.3468417308
C	6.7647258524	-0.6615608682	-1.1458915536
H	6.8700821080	-0.1077810103	-2.0897173435
H	7.6834683993	-0.5846378513	-0.5549635767
H	6.5467659163	-1.7135935392	-1.3773648267
H	1.5596446270	0.5521268145	0.4581026195
C	-1.2262897939	2.2556888591	0.5073237012
C	-2.5255439646	2.7361644844	-0.2324235935
C	-2.2543012984	3.2501420548	-1.6461594794
H	-3.2111497837	3.3589810510	-2.1760782807
H	-1.7530695329	4.2283036098	-1.6309094568
H	-1.6296706161	2.5455833924	-2.2147634998
C	-3.3629234059	3.7399859736	0.5432724238

H	-2.7807116995	4.6508339094	0.7485658833
H	-4.2462760150	4.0228820477	-0.0467863175
H	-3.7105605284	3.3227693157	1.4963141409
C	0.0324906017	3.0294313419	0.1490868421
H	-0.1069340378	4.0990120760	0.3672799079
H	0.8713437730	2.6643261596	0.7621822182
H	0.2793959026	2.9287922443	-0.9173015148
C	-1.3823948293	2.1966383437	2.0252475160
H	-2.3055527366	1.6710599401	2.3128106314
H	-0.5220671773	1.6542067390	2.4417537833
H	-1.4049879151	3.2036935015	2.4650380366
C	-0.9829258005	-1.6838442199	2.0063765471
H	-1.7268681877	-0.8892574402	2.1528610989
H	-1.2688882397	-2.5638711393	2.6032875437
H	-0.0036428380	-1.2911166807	2.3246202051

PR1a

0 1

C	-2.7015461123	-0.5202086355	-0.0134621292
C	-2.6098458196	-1.8594862619	-0.4162210048
C	-3.7341631528	-2.6420779802	-0.7009315906
C	-4.9991734256	-2.0833026411	-0.5642249724
C	-5.1304036375	-0.7634542141	-0.1258190251
C	-3.9938138745	-0.0065921293	0.1479675787
H	-3.6195105940	-3.6832710812	-1.0161704013
H	-5.8841674484	-2.6837653987	-0.7858824011
H	-6.1241878850	-0.3275558780	-0.0005319760
H	-4.1005815288	1.0231791056	0.4969436024
N	-1.3281898513	-2.4565902817	-0.5153650868
C	-0.2338717258	-1.9254483859	0.0500448736
N	-0.2426812382	-0.7476895238	0.5746829271
S	1.2272538651	-2.9436947542	-0.0056394988
H	-1.2324393948	-3.3362854303	-1.0053065688
B	-1.3540690427	0.3573064208	0.1975194208
O	-1.3508832713	1.3609750326	1.2319392128
O	-0.9253113855	1.0245522739	-1.0217707593
C	2.4623392328	-1.6649198443	-0.4240244000
H	3.1512760388	-2.1249382464	-1.1438185588
H	1.9276916825	-0.8542142317	-0.9386331858
C	3.2415939239	-1.1205571301	0.7850663088
H	3.9614762484	-1.9004290763	1.1045865867
N	2.3742108703	-0.6973677777	1.8654480480

H	2.7757492429	0.1160331968	2.3311281049
H	0.6031289061	-0.4892694009	1.1044638540
C	4.1063233413	0.0687213014	0.3713567484
O	4.2698473137	1.0430540413	1.0562697255
O	4.6698124893	-0.1113982174	-0.8173192633
C	5.4853710221	0.9505346392	-1.3002149210
H	6.3212729369	1.1397071894	-0.6125997375
H	5.8592427417	0.6269127465	-2.2770598966
H	4.8932659129	1.8706080861	-1.4022957271
H	2.2359470924	-1.4343258049	2.5506911342
C	-0.3238544063	2.2508835362	-0.6740526002
C	-1.0671695822	2.6236075761	0.6642019833
C	-2.3901461201	3.3521330370	0.3985851623
H	-2.9707015129	3.3734942829	1.3328245155
H	-2.2370390372	4.3880635595	0.0600579789
H	-2.9812437217	2.8207525731	-0.3611233836
C	-0.2308658392	3.4368019229	1.6476270646
H	0.0954583733	4.3897446592	1.2019266899
H	-0.8298473012	3.6642079355	2.5419253091
H	0.6561164778	2.8778343645	1.9740143457
C	-0.5267065253	3.2460853963	-1.8129513232
H	-0.1743027934	4.2513210801	-1.5327011940
H	0.0407435170	2.9173000190	-2.6966610453
H	-1.5835436555	3.3074650066	-2.1009282367
C	1.1868221796	2.0453918313	-0.4658249695
H	1.3977369289	1.4009922967	0.3982731382
H	1.6002166938	1.5653247990	-1.3675948479
H	1.7197568516	2.9950464160	-0.3081516085

PR1b

0 1

C	2.6651562414	-0.1197714016	-0.1524955646
C	2.7564428319	-1.4519992758	0.2770175977
C	3.9914306379	-2.1127054798	0.3558622766
C	5.1540279989	-1.4399838017	-0.0050021395
C	5.0874601326	-0.1230788849	-0.4595576044
C	3.8519654641	0.5126562288	-0.5375658931
H	4.0615114727	-3.1530194996	0.6747976634
H	6.1142906773	-1.9570778844	0.0558552196
H	5.9988994256	0.4013128821	-0.7556848059
H	3.7897290146	1.5378535304	-0.9098640283
N	1.5594689007	-2.1687874161	0.6167058870

C	0.3801474757	-1.7718219758	0.0874563875
N	0.2378691785	-0.6204671295	-0.4814035144
S	-1.0070519857	-2.8957587025	0.1915772360
B	1.2241902911	0.6068573430	-0.2057380800
O	1.0058896443	1.5922694267	-1.2383921991
O	0.8469577621	1.2404199407	1.0492678045
C	-2.3519833414	-1.7112560968	0.5316973333
H	-3.0028483629	-2.1957442802	1.2706926517
H	-1.8971025762	-0.8310783020	1.0064005070
C	-3.1720808866	-1.2982312201	-0.7030989056
H	-3.7995641608	-2.1618735966	-0.9990633399
N	-2.3431233140	-0.8233572734	-1.7911451808
H	-2.7933022970	-0.0341248991	-2.2538610373
H	-0.6304663611	-0.4821541053	-1.0212641756
C	-4.1609849783	-0.1949729211	-0.3310096731
O	-4.3824024625	0.7637331393	-1.0218496384
O	-4.7559057796	-0.4293990767	0.8328422752
C	-5.6826090891	0.5534133968	1.2811050656
H	-6.5027977992	0.6697195021	0.5592208495
H	-6.0666309591	0.1939012022	2.2413523803
H	-5.1804571743	1.5225706066	1.4081794184
H	-2.1598289063	-1.5486650918	-2.4778012911
C	0.0807773832	2.3860947559	0.7564581731
C	0.6494321151	2.8236010553	-0.6454220000
C	1.9088534255	3.6874034253	-0.5052999311
H	2.3911875627	3.7642785706	-1.4911772351
H	1.6823024911	4.7039377151	-0.1494126684
H	2.6226290594	3.2241325875	0.1911587207
C	-0.3571483378	3.5374186452	-1.5428295527
H	-0.7365721521	4.4554060979	-1.0667015796
H	0.1267263507	3.8180506327	-2.4901608783
H	-1.2086599434	2.8870106463	-1.7833364091
C	0.2713644841	3.4118932665	1.8697969255
H	-0.2150898854	4.3680141650	1.6198196034
H	-0.1739677309	3.0334441032	2.8023049525
H	1.3363629271	3.5941366819	2.0603477858
C	-1.4091842311	2.0083807314	0.6845539239
H	-1.6158915261	1.3362368136	-0.1596856549
H	-1.6828230455	1.4936335615	1.6195033507
H	-2.0587549675	2.8896907984	0.5739746124
C	1.6759686136	-3.3324738465	1.4813248065
H	2.0140179530	-4.2248112210	0.9307446263
H	2.4005282298	-3.1118080684	2.2757266668
H	0.7086465092	-3.5522740010	1.9439092797

PR2a

0 1

C	2.5590518847	1.6568236826	0.1579087565
C	1.1782943573	1.9789958250	0.0713075396
C	0.7763328103	3.3241629360	0.0644171808
C	1.7326207031	4.3327408789	0.1277943592
C	3.0934320372	4.0388787206	0.2101887181
C	3.4881251401	2.7061942233	0.2278064172
H	-0.2864194983	3.5519908284	0.0187980036
H	1.3993383772	5.3736364724	0.1182805316
H	3.8340830664	4.8390674105	0.2641025983
H	4.5492115158	2.4531328177	0.2965854877
N	0.2649161393	0.9237696444	-0.0022291904
C	-1.1000383713	0.9578169282	-0.1514268946
N	-1.8311991889	1.9858777623	-0.0627946209
S	-1.6618915074	-0.7418870630	-0.5179997743
H	0.6808641784	-0.0032652885	0.0467621822
B	3.1044439860	0.2038614055	0.1889149543
O	4.4175476389	-0.1206658425	0.3847499880
O	2.3203166235	-0.9188809843	0.0341509868
C	-3.3230999659	-0.7505456698	0.2174855501
H	-3.4552097856	-1.7420615494	0.6699455783
H	-3.3450976079	-0.0011030984	1.0225075250
C	-4.4453741810	-0.4775785240	-0.7923851062
H	-4.4578795346	-1.3223003367	-1.5131476209
N	-4.2880312872	0.8092944117	-1.4335158310
H	-5.1963911964	1.1806211831	-1.7044007500
H	-2.8024176927	1.7996923954	-0.3357647809
C	-5.8171967758	-0.5267368747	-0.1301644662
O	-6.7140595820	0.2346235374	-0.3802130259
O	-5.9254428903	-1.5401064300	0.7269144974
C	-7.1849594326	-1.6986078817	1.3649651415
H	-7.9770643803	-1.8719130098	0.6227473942
H	-7.0858615880	-2.5668114000	2.0251594937
H	-7.4397919667	-0.8027101646	1.9483175280
H	-3.6966063240	0.7396032279	-2.2568670884
C	3.1127504720	-2.0706154357	0.3709656106
C	4.5723284426	-1.5277515190	0.1413338202
C	5.0458601606	-1.6850013611	-1.3036979130
H	5.9700214914	-1.1055502598	-1.4380647997
H	5.2548040991	-2.7358455220	-1.5501506040

H	4.2991201696	-1.2997491816	-2.0131212104
C	5.6132220409	-2.0859014280	1.0995759053
H	5.6819547367	-3.1800192581	1.0030486107
H	6.5983932648	-1.6585220026	0.8650877880
H	5.3771087670	-1.8367533762	2.1415459964
C	2.7072949272	-3.2283714858	-0.5270412568
H	3.3582269729	-4.0985642991	-0.3541254265
H	1.6718418496	-3.5229832759	-0.3041446494
H	2.7587412885	-2.9537291440	-1.5878374633
C	2.8165947755	-2.4033118287	1.8328991180
H	3.1134486960	-1.5795455427	2.4980774143
H	1.7347230571	-2.5603258237	1.9487712184
H	3.3369230869	-3.3171704301	2.1530735788

PR2b

0 1

C	-2.7145370114	-1.0244550542	-0.2449439688
C	-2.1070282478	-2.1466157831	0.3488568325
C	-2.6777786243	-3.4117701628	0.2280685078
C	-3.8658798919	-3.5830008170	-0.4796619962
C	-4.4819280455	-2.4861679759	-1.0780708315
C	-3.9050545568	-1.2238344583	-0.9595138554
H	-2.1705465493	-4.2615614530	0.6885426949
H	-4.3054345228	-4.5789679655	-0.5707724251
H	-5.4100629386	-2.6149474297	-1.6391060085
H	-4.3856701817	-0.3616968710	-1.4282534892
N	-0.9072263861	-2.0132539247	1.1193709584
C	0.2589284253	-1.7789653584	0.4194843433
N	0.3082663645	-1.9658844693	-0.8342652901
S	1.5915289679	-1.2113835137	1.5095539864
B	-2.1578053285	0.4301942457	-0.1363001729
O	-2.7957876898	1.4918450408	-0.7193146845
O	-1.0533814579	0.8271324579	0.5768888583
C	2.9906825844	-1.0068748628	0.3791562452
H	3.8743467992	-1.1229869862	1.0196953689
H	3.0032455060	-1.8254131913	-0.3568814927
C	3.0323762198	0.3566688937	-0.3172604854
H	2.9801434885	1.1262385534	0.4867614736
N	1.9901466221	0.4835862401	-1.3066188359
H	2.1579248381	1.2861289031	-1.9080552559
H	1.1777307836	-1.6708110619	-1.2705993732
C	4.3677554077	0.6321357542	-0.9992651451

O	4.4820812478	1.2724358871	-2.0106578137
O	5.4035939938	0.1436054307	-0.3163092452
C	6.6934468768	0.4172292994	-0.8433085695
H	6.8730868448	1.5008962659	-0.8885017691
H	7.4089630772	-0.0586404003	-0.1641265398
H	6.7951342771	0.0027586491	-1.8561681056
H	1.0727184552	0.5563088967	-0.8713853206
C	-1.1228938294	2.2555657609	0.7207958629
C	-1.9815690328	2.6601137611	-0.5305611393
C	-1.1432811509	2.8381267659	-1.7964183136
H	-1.8167793948	2.9008331462	-2.6624961520
H	-0.5396742026	3.7562888303	-1.7571007835
H	-0.4727489610	1.9816474912	-1.9568101414
C	-2.8835035802	3.8665801057	-0.3217014420
H	-2.2874630485	4.7567932996	-0.0697921670
H	-3.4405015550	4.0776095956	-1.2455576794
H	-3.6126708282	3.6909347783	0.4790733338
C	0.2845428253	2.8291996778	0.7567290208
H	0.2532247472	3.9266052336	0.8291356633
H	0.8143051498	2.4362816502	1.6365005592
H	0.8611114833	2.5534578786	-0.1338719534
C	-1.8316091818	2.5347197136	2.0469034043
H	-2.8559332984	2.1340116600	2.0449817481
H	-1.2744980532	2.0424511518	2.8568870364
H	-1.8779977285	3.6113775797	2.2638462863
C	-1.0674172022	-1.7258923050	2.5307417390
H	-1.1444776486	-0.6416977715	2.7276487328
H	-1.9865911666	-2.2131231169	2.8816519114
H	-0.2307606903	-2.1377486656	3.1131448774

RE1a

0 1

C	2.8766742488	0.1730010477	-0.5100757092
C	2.6894036057	-1.2197656439	-0.5160091246
C	3.7697015695	-2.0921108542	-0.3662658636
C	5.0589083057	-1.5864908157	-0.2264187204
C	5.2772261101	-0.2102643926	-0.2690559464
C	4.1933310848	0.6512029631	-0.4139622763
H	3.5914864079	-3.1691905263	-0.3815605111
H	5.8988595300	-2.2744628822	-0.1077199343
H	6.2895090417	0.1894448556	-0.1823724573
H	4.3562477014	1.7315312154	-0.4268662698

N	1.3996072967	-1.7922065422	-0.6866689831
C	0.5217677152	-1.3787082087	-1.5876166114
N	-0.2713338616	-1.0603806058	-2.3764522921
S	-0.9290156624	-3.2402577188	1.1899567098
B	1.7184561914	1.2241834076	-0.4379768931
O	1.8834036284	2.5189111476	-0.8341523178
O	0.5036473472	0.9985938934	0.1443125161
C	-1.7416667883	-1.6061134680	1.0749040538
H	-1.9140124346	-1.2580904749	2.1011132306
H	-1.0715956459	-0.8830405228	0.5833149181
C	-3.0769538072	-1.7123901836	0.3256258443
H	-3.7203891182	-2.4004849167	0.9045948258
N	-2.8882408646	-2.2616541466	-0.9919319027
H	-3.7787701423	-2.3886026100	-1.4644993049
H	-2.3233030492	-1.6407220538	-1.5754153456
C	-3.8217631263	-0.3760497453	0.3251384087
O	-4.2893346142	0.1445095326	-0.6520828164
O	-3.9384595992	0.1359302056	1.5565030781
C	-4.6841108178	1.3390243264	1.6704557861
H	-5.7213726924	1.1846726311	1.3402145777
H	-4.6597938238	1.6161589617	2.7299862762
H	-4.2361299602	2.1341895220	1.0577437994
H	-1.4027876631	-3.6361584195	-0.0181170513
C	-0.2994339413	2.1826349019	-0.0293411774
C	0.7943403426	3.2874546890	-0.2935264544
C	1.3113565641	3.9431294768	0.9871052571
H	2.1956612347	4.5490041284	0.7437558028
H	0.5551912739	4.5997836891	1.4404280008
H	1.6114114800	3.1894395170	1.7297663332
C	0.3917166631	4.3517345166	-1.3021226475
H	-0.4958179818	4.9003972957	-0.9527564777
H	1.2130099103	5.0715082179	-1.4278374338
H	0.1715725986	3.9129919023	-2.2828475808
C	-1.1222907331	2.3918935813	1.2327499623
H	-1.6961033447	3.3289983654	1.1698238456
H	-1.8304113571	1.5601848636	1.3510410855
H	-0.4921307066	2.4237873297	2.1302431292
C	-1.2157165470	1.9456460102	-1.2260001240
H	-0.6422700213	1.7649283762	-2.1452374647
H	-1.8283903081	1.0521032994	-1.0521205305
H	-1.8931397575	2.7966492539	-1.3852196078
H	1.0346338881	-2.4579323729	0.0033855688

RE1b

0 1

C	3.0581481893	0.2381606188	-0.4215338791
C	3.0726700046	-1.1487361140	-0.1789333659
C	4.2765430174	-1.8604333242	-0.1777485003
C	5.4775445923	-1.2076372876	-0.4400039254
C	5.4849329805	0.1556441897	-0.7242371902
C	4.2836876106	0.8581376026	-0.7149511693
H	4.2752087183	-2.9350331232	0.0090791557
H	6.4098436125	-1.7766828518	-0.4385155779
H	6.4226307732	0.6695776799	-0.9439549582
H	4.2816605645	1.9316464687	-0.9165622651
N	1.8667998999	-1.8636770712	0.0775468150
C	0.8191110773	-1.7169408048	-0.7253079444
N	-0.1123637402	-1.6362217356	-1.4159031283
S	-1.8795820308	-3.2212099922	1.8975219097
B	1.8148223079	1.1808692982	-0.2791526651
O	1.8356405858	2.4494952303	-0.7890433037
O	0.6696398721	0.9086832137	0.4106808689
C	-2.3439855291	-1.5480669605	1.3425735289
H	-2.6570115480	-0.9944269543	2.2364574236
H	-1.4669825056	-1.0385971449	0.9137730140
C	-3.4860637905	-1.5921537637	0.3227939025
H	-4.3398882699	-2.0982516667	0.8133711641
N	-3.1051026452	-2.3525022809	-0.8399836245
H	-3.8474331649	-2.3408897570	-1.5341359562
H	-2.2686857328	-1.9560856619	-1.2754188548
C	-4.0155641538	-0.1962290661	-0.0170457905
O	-4.3521617612	0.1525446716	-1.1169400417
O	-4.1257438078	0.5877966288	1.0637228234
C	-4.6741250410	1.8782844437	0.8525078554
H	-5.6974868509	1.8073907823	0.4568665390
H	-4.6774198507	2.3751246701	1.8290801277
H	-4.0629447965	2.4483333414	0.1381365946
H	-2.0157560582	-3.7613252107	0.6648158345
C	-0.2536831945	1.9863333093	0.1538332161
C	0.7174909295	3.1602686475	-0.2302551496
C	1.2374678631	3.9286823696	0.9848099287
H	2.0636029578	4.5805286338	0.6670990054
H	0.4534880276	4.5557060900	1.4327478363
H	1.6207968027	3.2446219530	1.7559429499
C	0.1691822580	4.1254414709	-1.2691684587
H	-0.7498833106	4.6066062133	-0.9019016824

H	0.9096587190	4.9119410948	-1.4731489004
H	-0.0537114673	3.6157617553	-2.2144123418
C	-1.0872357362	2.2338511814	1.4006232358
H	-1.6975537810	3.1417389260	1.2774977369
H	-1.7686533492	1.3891921888	1.5702518676
H	-0.4570200548	2.3526471819	2.2909283447
C	-1.1430422783	1.5501256624	-1.0071611104
H	-0.5629196217	1.4048347177	-1.9290878160
H	-1.5990723075	0.5821293529	-0.7698189263
H	-1.9432130310	2.2774308783	-1.2042500242
C	1.8412367440	-2.9759243600	1.0243153449
H	2.2000007002	-3.9154572922	0.5728101418
H	2.4766041546	-2.7203349461	1.8823835151
H	0.8105654465	-3.1194290970	1.3790418705

RE2a

0 1

C	2.5965789370	1.8666232439	0.1855725399
C	1.2721063077	2.3476881202	0.2601129404
C	1.0125699206	3.7224735744	0.2831241594
C	2.0688720781	4.6255784583	0.2345440593
C	3.3884170087	4.1772386377	0.1669849728
C	3.6357920737	2.8091217606	0.1445124194
H	-0.0174042701	4.0810738406	0.3405979454
H	1.8537244370	5.6964034284	0.2520497972
H	4.2137149685	4.8904751673	0.1317076122
H	4.6637922548	2.4422837746	0.0909302013
N	0.2118378616	1.4267562955	0.3202473793
C	-1.0668455605	1.7702542091	0.3588416385
N	-2.1885660214	2.0749572935	0.3889432691
S	-1.3871043347	-1.7390858897	0.3395353185
H	0.4325634493	0.4264991955	0.3218311145
B	2.9430486956	0.3513355668	0.1409801651
O	4.2054286760	-0.1506998081	0.2422623031
O	1.9971069260	-0.6354608185	-0.0010908927
C	-3.0975826742	-1.1994603094	0.6669558515
H	-3.5444393620	-1.9291255533	1.3532402747
H	-3.0842108098	-0.2168800793	1.1616455574
C	-3.9227560792	-1.1156277457	-0.6224031186
H	-3.9619639675	-2.1300971558	-1.0603231682
N	-3.3024052684	-0.2245956139	-1.5725176193
H	-3.8431878277	-0.1917210628	-2.4328836353

H	-3.2883779237	0.7256174477	-1.1959757717
C	-5.3753008415	-0.7412610201	-0.3157271317
O	-5.9995720221	0.0968117031	-0.9086664160
O	-5.8895284855	-1.4854221626	0.6686123339
C	-7.2435861130	-1.2322049245	1.0098724004
H	-7.9030937256	-1.4117796127	0.1485959562
H	-7.4893002491	-1.9209901984	1.8255856553
H	-7.3741092007	-0.1912535549	1.3383643317
C	2.6388644270	-1.9038283071	0.2480387853
C	4.1459195925	-1.5588293670	-0.0515227114
C	4.5155442198	-1.7224566183	-1.5254937459
H	5.5038595114	-1.2725115673	-1.6949903851
H	4.5611625355	-2.7814542465	-1.8162681291
H	3.7935711088	-1.2110527366	-2.1785850040
C	5.1471476856	-2.2909570705	0.8278057288
H	5.0559143293	-3.3792296712	0.6930348190
H	6.1690273075	-1.9955586695	0.5509513226
H	5.0016901750	-2.0533360583	1.8888405451
C	2.0342132171	-2.9586557094	-0.6643880642
H	2.5880701248	-3.9051919664	-0.5737858564
H	0.9893655635	-3.1474915802	-0.3786756860
H	2.0488591649	-2.6415592258	-1.7146008233
C	2.3751750337	-2.2519746258	1.7116936140
H	2.8216223086	-1.5078067632	2.3873836045
H	1.2889659241	-2.2602415219	1.8801871776
H	2.7763128079	-3.2427012464	1.9679909061
H	-1.3243271049	-1.1160753062	-0.8610998707

TS1a

0 1

C	2.8575148838	-0.4414113523	-0.1216427014
C	2.5819203930	-1.7764908806	0.2226016613
C	3.6175150360	-2.7053872692	0.3725080398
C	4.9384845751	-2.3232087753	0.1616077371
C	5.2329725178	-1.0193996429	-0.2322810497
C	4.1954852187	-0.1008579160	-0.3731287651
H	3.3778240472	-3.7380713282	0.6372553919
H	5.7379877394	-3.0574628354	0.2840399087
H	6.2652732842	-0.7189709426	-0.4223423051
H	4.4217097913	0.9278762158	-0.6648757496
N	1.2602031642	-2.2297609746	0.4130063540
C	0.2228318619	-1.8037023859	-0.3290435669

N	-0.1829542560	-1.0445140854	-1.1504198227
S	-1.4552182391	-3.3161138058	0.6145574348
H	1.0726210778	-3.0182182469	1.0178695429
B	1.8321113774	0.7459077537	-0.0859064076
O	1.9215592574	1.8011390367	-0.9504799507
O	0.9508045079	0.9749440110	0.9356721265
C	-2.4987045379	-1.8525791679	0.8873843472
H	-3.1662606873	-1.9950585929	1.7482441559
H	-1.8671138336	-0.9784623449	1.1043903524
C	-3.3731163555	-1.5435014370	-0.3450679219
H	-4.2609118560	-2.1982448489	-0.3287359839
N	-2.6127979209	-1.8402468079	-1.5715956463
H	-3.1239414717	-1.6198295599	-2.4265442950
H	-1.6224020432	-1.3259321759	-1.5434358713
C	-3.8708491722	-0.1141985559	-0.4340036103
O	-3.8449592001	0.5396852875	-1.4451042042
O	-4.3642096540	0.3030319897	0.7225031798
C	-4.8718552932	1.6312648157	0.7588573131
H	-5.6530811007	1.7693329739	-0.0013736838
H	-5.2846950223	1.7712693854	1.7631493856
H	-4.0633726552	2.3523168466	0.5754730818
H	-2.3230578305	-2.8343774815	-1.4837747441
C	0.2651995757	2.2073345125	0.6725413223
C	1.2134936420	2.9082619029	-0.3792603208
C	2.2630699704	3.8138438743	0.2670749132
H	2.9919571115	4.1134971643	-0.4994339085
H	1.8137461337	4.7222532539	0.6933240432
H	2.8080355786	3.2853530613	1.0628874452
C	0.4902523603	3.6565695901	-1.4893589332
H	-0.1216223139	4.4728718452	-1.0762468042
H	1.2269485328	4.0945831278	-2.1778042191
H	-0.1565758272	2.9859346023	-2.0681869073
C	0.1062505194	2.9633099549	1.9851654028
H	-0.3444222714	3.9527358534	1.8133173004
H	-0.5545765268	2.3959194415	2.6561483350
H	1.0676172039	3.0972189961	2.4962968733
C	-1.1115121248	1.8688339573	0.1033572412
H	-1.0288046616	1.2745075176	-0.8152555475
H	-1.6608309163	1.2701604490	0.8456154800
H	-1.6927905897	2.7787669939	-0.1057274489

TS2a

0 1

C	2.4239118876	1.7657477507	0.1009408359
C	1.0632871479	2.0647135668	-0.1592798876
C	0.6029777661	3.3859792549	-0.0852410646
C	1.4907700296	4.4087168286	0.2329038889
C	2.8357383031	4.1404832951	0.4897181547
C	3.2832331211	2.8247972845	0.4270563407
H	-0.4506226657	3.5971373416	-0.2716419199
H	1.1194091032	5.4351989532	0.2838537603
H	3.5239128785	4.9493418179	0.7421789286
H	4.3307872488	2.5958372668	0.6395886997
N	0.2009972459	1.0065734343	-0.4689677033
C	-1.1328998735	1.0960383889	-0.5886265715
N	-2.1130691359	1.7744881410	-0.5593177428
S	-1.5415845170	-1.2688291528	-0.9970198923
H	0.6026422357	0.0729147700	-0.5295097698
B	2.9677236410	0.3086982630	0.0811602378
O	4.1323997313	-0.1053364035	0.6597482464
O	2.2801744502	-0.7337501539	-0.4931332316
C	-2.9971173528	-1.1927486676	0.0876847271
H	-3.1869722757	-2.1639190124	0.5651050927
H	-2.8321215906	-0.4580211765	0.8912419351
C	-4.2590506469	-0.7868289237	-0.6905015813
H	-4.6169505024	-1.6530050503	-1.2732492928
N	-3.9283274833	0.2989566565	-1.6310268003
H	-4.7605584081	0.7184843699	-2.0485244640
H	-3.2780956328	1.0875397435	-1.1161589771
C	-5.4201646932	-0.3161446356	0.1649017313
O	-6.0881811275	0.6519632323	-0.0959546404
O	-5.6260182020	-1.1161739045	1.1995401295
C	-6.7052057963	-0.7777893717	2.0625576171
H	-7.6553171500	-0.7716572592	1.5101819337
H	-6.7220492498	-1.5453116366	2.8430922596
H	-6.5463357009	0.2149333036	2.5059649110
H	-3.2705217679	-0.0925952833	-2.3209587716
C	2.8662117318	-1.9621266439	-0.0316118968
C	4.3261590766	-1.4929691765	0.3246901924
C	5.2751425513	-1.5378236382	-0.8721168193
H	6.2031514130	-1.0103372469	-0.6098676919
H	5.5281128275	-2.5713775433	-1.1486116920
H	4.8361699236	-1.0385079933	-1.7480103031
C	4.9436065564	-2.2072759226	1.5164970816
H	5.0119110293	-3.2887747270	1.3246285492
H	5.9587820266	-1.8249786910	1.6945920401

H	4.3565668585	-2.0475544129	2.4293984649
C	2.7670685727	-2.9998662182	-1.1375951275
H	3.2809415218	-3.9278667164	-0.8446669484
H	1.7083277792	-3.2316065370	-1.3222340196
H	3.2032990890	-2.6343891956	-2.0756076035
C	2.0495695581	-2.4050724650	1.1824520483
H	2.1294548375	-1.6783251418	2.0044961695
H	0.9922679495	-2.4663537828	0.8837835490
H	2.3737746799	-3.3883369793	1.5523768879

TS2b

0 1

C	-3.0409683280	-1.0680044907	-0.3255624524
C	-2.3880297534	-2.2633040781	0.0108487119
C	-3.0204553497	-3.4993840596	-0.1171305040
C	-4.3293829244	-3.5621129337	-0.5885589016
C	-4.9917022940	-2.3909992309	-0.9536780103
C	-4.3472494592	-1.1625029003	-0.8296382095
H	-2.4720323190	-4.4074949634	0.1410132776
H	-4.8249998966	-4.5300684906	-0.6888199146
H	-6.0122442279	-2.4365465617	-1.3395014644
H	-4.8656848672	-0.2454564199	-1.1192166778
N	-1.0337107942	-2.2440382161	0.4920062343
C	-0.1001687270	-1.7604900627	-0.3147125148
N	0.7116630004	-1.3679323310	-1.0504998304
S	2.2328015471	-0.1656656402	2.1569790753
B	-2.4285192654	0.3617694556	-0.1160188223
O	-2.8607596855	1.4297144019	-0.8422771044
O	-1.5108403339	0.6993330906	0.8415097071
C	3.3750581680	-0.4166810125	0.7656913844
H	4.3290146925	-0.7144110620	1.2188759734
H	3.0161232048	-1.2524612568	0.1464280343
C	3.5836205916	0.8227233632	-0.1084701320
H	3.9491988747	1.6360445917	0.5466883274
N	2.3544052797	1.2345260881	-0.7419348434
H	2.5525702444	1.9173020340	-1.4699329820
H	1.9125072140	0.4323157460	-1.1977305002
C	4.7084642147	0.5872821453	-1.1220766210
O	4.6412812096	0.8708669149	-2.2886483658
O	5.8047208461	0.0739162431	-0.5540847767
C	6.9188016036	-0.1364020873	-1.4069381413
H	7.2499246725	0.8105873422	-1.8571249056

H	7.7126662038	-0.5552402136	-0.7787014389
H	6.6644294915	-0.8364920616	-2.2155359377
C	-1.5120079397	2.1460907594	0.9304475513
C	-2.0210350113	2.5497626935	-0.4968184963
C	-0.8952511696	2.5986231697	-1.5282588703
H	-1.3360781109	2.6431133370	-2.5341499719
H	-0.2583124612	3.4826777661	-1.3840447184
H	-0.2596197945	1.7043042529	-1.4627898923
C	-2.8501082590	3.8225524493	-0.5431590824
H	-2.2612948884	4.6781213031	-0.1797494581
H	-3.1520641704	4.0304874172	-1.5795006545
H	-3.7597426853	3.7361795215	0.0646420307
C	-0.1213783157	2.6537382472	1.2664045601
H	-0.1081946707	3.7540444221	1.2212501306
H	0.1508144988	2.3596220408	2.2907119815
H	0.6454946152	2.2599779015	0.5826151163
C	-2.4973029172	2.5109709031	2.0407575672
H	-3.5195778945	2.1824836718	1.8008474523
H	-2.1865405910	2.0105588582	2.9687850496
H	-2.5120696065	3.5949499932	2.2225294197
C	-0.8002862410	-2.3073782175	1.9332781173
H	-1.2162287970	-1.4162751699	2.4289153463
H	-1.2752894435	-3.2123305352	2.3371674451
H	0.2792836875	-2.3441152041	2.1263593588
H	1.1582772720	0.1730311751	1.4159272326

TS1b

0 1

C	2.7853971135	0.0813827747	-0.3592701533
C	2.7870788961	-1.2706978478	0.0278219521
C	3.9313010814	-2.0570893249	-0.1367419114
C	5.0816298074	-1.5115407732	-0.7024951028
C	5.0899669135	-0.1861325447	-1.1275231046
C	3.9455537908	0.5909044786	-0.9564541847
H	3.9197892130	-3.1070315859	0.1595078731
H	5.9681311049	-2.1375057043	-0.8274968020
H	5.9834430823	0.2415995265	-1.5869569834
H	3.9491338846	1.6355061205	-1.2784183707
N	1.6129946977	-1.8727387625	0.5791664416
C	0.4520647073	-1.5634491725	-0.0269127205
N	-0.0910781288	-0.8102957242	-0.7731604554
S	-1.1816436600	-3.3779396485	0.6442793316

B	1.6209098921	1.0960162534	-0.0652324663
O	1.2632524960	2.0767660042	-0.9449908748
O	1.0614599319	1.2627041822	1.1727928614
C	-2.4517903024	-2.0854696999	0.7265899043
H	-3.2252567120	-2.3370055078	1.4649142124
H	-1.9924464897	-1.1380966251	1.0429914471
C	-3.1514767088	-1.8468680520	-0.6320391293
H	-3.9625441142	-2.5822847850	-0.7630344137
N	-2.1694170839	-2.0357121715	-1.7098042874
H	-2.5498233711	-1.9063317368	-2.6467933231
H	-1.3100678563	-1.3600299454	-1.5062757681
C	-3.7424595852	-0.4579468101	-0.7766538215
O	-3.5505129585	0.2560058158	-1.7277262418
O	-4.4992928425	-0.1392531015	0.2630161896
C	-5.0898611263	1.1542184508	0.2452006576
H	-5.7621529176	1.2616433698	-0.6175846760
H	-5.6513760670	1.2451191874	1.1806644565
H	-4.3125861430	1.9286749060	0.1881864108
H	-1.7430047786	-2.9704546611	-1.5313740838
C	0.0913470504	2.3136497621	1.0966202862
C	0.4807440724	3.0546184124	-0.2505441207
C	1.3870486892	4.2673308932	-0.0360564286
H	1.7414311970	4.6211011490	-1.0147635169
H	0.8577079342	5.0928368487	0.4610806602
H	2.2686442780	4.0025669294	0.5652500333
C	-0.7067781774	3.4387695266	-1.1240592306
H	-1.3628146642	4.1509898535	-0.6004939837
H	-0.3437543157	3.9204720122	-2.0432123661
H	-1.2957809327	2.5597170528	-1.4143785333
C	0.2124679314	3.1722926456	2.3497893063
H	-0.4658166720	4.0375068239	2.2992909818
H	-0.0602508885	2.5731327855	3.2303025245
H	1.2372937406	3.5354472028	2.4956690377
C	-1.2936705410	1.6663660485	1.0560699491
H	-1.3990322406	1.0282738960	0.1698303273
H	-1.4125594013	1.0345753360	1.9484268022
H	-2.0877551497	2.4274094502	1.0609616373
C	1.7522577273	-2.8395218392	1.6548655999
H	1.9298276296	-3.8611439745	1.2799256783
H	2.5997342757	-2.5331610908	2.2825767088
H	0.8355006907	-2.8563066092	2.2512497838

15. References

(1) Froidevaux, V.; Borne, M.; Laborbe, E.; Auvergne, R.; Gandini, A.; Boutevin, B. Study of the Diels–Alder and retro-Diels–Alder reaction between furan derivatives and maleimide for the creation of new materials. *RSC Advances*, **2015**, 5, 37742. https://doi.org/10.1039/C5RA01185J

(2) Yin, Y.; Chen, C.; Yu, R.; Shu, L.; Wang, Z.; Zhang, T. Novel 1*H*-Pyrazolo[3,4-*d*]pyrimidin-6-amino Derivatives as Potent Selective Janus Kinase 3 (JAK3) Inhibitors. Evaluation of Their Improved Effect for the Treatment of Rheumatoid Arthritis. *Bioorganic Chemistry* **2020**, 98, 103720. https://doi.org/10.1016/j.bioorg.2020.103720

(3) Basavaprabhu, H.; Sureshbabu, V. Iron(III) Catalysed Synthesis of Unsymmetrical Di and Trisubstituted Ureas - A Variation of Classical Ritter Reaction. *Organic and Biomolecular Chemistry* **2012**, 10 (13), 2528–2533. https://doi.org/10.1039/c2ob06916d

(4) Vedejs, E.; Kongkittingam, C. Solution-Phase Synthesis of a Hindered *N*-Methylated Tetrapeptide Using Bts-Protected Amino Acid Chlorides: Efficient Coupling and Methylation Steps Allow Purification by Extraction. *Journal of Organic Chemistry* **2000**, 65 (8), 2309–2318. https://doi.org/10.1021/jo9914115

(5) Granchi, C; Rizzolio, F.; Palazzolo, S.; Carmignani, S.; Macchia, M.; Saccomanni, G.; Manera, C.; Martinelli, A.; Minutolo, F.; Tuccinardi, T. Structural Optimization of 4-Chlorobenzoylpiperidine Derivatives for the Development of Potent, Reversible, and Selective Monoacylglycerol Lipase (MAGL) Inhibitors. *Journal of Medicinal Chemistry* **2016**, 59 (22) , 10299-10314. https://doi.org/10.1021/acs.jmedchem.6b01459

(6) Bargh, J.; Walsh, S.; Isidro-Llobet, A.; Omarjee, S.; Carroll, J.; Spring, D. Sulfatase-cleavable linkers for antibody-drug conjugates. *Chemical science* **2020**, 11 (9), 2375–2380. https://doi.org/10.1039/c9sc06410a

(7) Auzzas, L., Larsson, A., Matera, R., Baraldi, A., Deschênes-Simard, B., Giannini, G., Cabri, W., Battistuzzi, G., Gallo, G., Ciacci, A., Vesci, L., Pisano, C., & Hanessian, S.. Non-natural macrocyclic inhibitors of histone deacetylases: design, synthesis, and activity. *Journal of medicinal chemistry* **2010**, *53* (23), 8387–8399. https://doi.org/10.1021/jm101092u

(8) António, J. P. M.; Carvalho, J. I.; André, A. S.; Dias, J. N. R.; Aguiar, S. I.; Faustino, H.; Lopes, R. M. R. M.; Veiros, L. F.; Bernardes, G. J. L.; da Silva, F. A.; Gois, P. M. P. Diazaborines Are a Versatile Platform to Develop ROS-Responsive Antibody Drug Conjugates. *Angewandte Chemie - International Edition* **2021**, 60 (49), 25914–25921. https://doi.org/10.1002/anie.202109835

(9) Ding, S.; Dong, X.; Gao, Z.; Zheng, X.; Ji, J.; Zhang, M.; Liu, F.; Wu, S.; Li, M.; Song, W.; Shen, J.; Duan, W.; Liu, J.; Chen, Y. Design, synthesis and biological evaluation of novel N-(3-amino-4-methoxyphenyl)acrylamide derivatives as selective $EGFR^{L858R/T790M}$ kinase inhibitors. *Bioorganic chemistry* **2022**, 118, 105471. https://doi.org/10.1016/j.bioorg.2021.105471

(10) Frisch, M. J.; Trucks, G. W.; Schlegel, H. B.; Scuseria, G. E.; Robb, M. A.; Cheeseman, J. R.; Scalmani, G.; Barone, V.; Mennucci, B.; Petersson, G. A.; Nakatsuji, H.; Caricato, M.; Li, X.; Hratchian, H. P.; Izmaylov, A. F.; Bloino, J.; Zheng, G.; Sonnenberg, J. L.; Hada, M.; Ehara, M.; Toyota, K.; Fukuda, R.; Hasegawa, J.; Ishida, M.; Nakajima, T.; Honda, Y.; Kitao, O.; Nakai, H.; Vreven, T.; Montgomery, J., J. A.; Peralta, J. E.; Ogliaro, F.; Bearpark, M.; Heyd, J. J.; Brothers, E.; Kudin, K. N.; Staroverov, V. N.; Kobayashi, R.; Normand, J.; Raghavachari, K.; Rendell, A.; Burant, J. C.; Iyengar, S. S.; Tomasi, J.; Cossi, M.; Rega, N.; Millam, J. M.; Klene, M.; Knox, J. E.; Cross, J. B.; Bakken, V.; Adamo, C.; Jaramillo, J.; Gomperts, R.; Stratmann, R. E.; Yazyev, O.; Austin, A. J.; Cammi, R.; Pomelli, C.; Ochterski, J. W.; Martin, R. L.; Morokuma, K.; Zakrzewski, V. G.; Voth, G. A.; Salvador, P.; Dannenberg, J. J.; Dapprich, S.; Daniels, A. D.; Farkas, Ö.; Foresman, J. B.; Ortiz, J. V.; Cioslowski, J.; Fox, D. J.; Gaussian, Inc., Wallingford CT,: **2009**

(11) Chai, J. D.; Head-Gordon, M. Long-range corrected hybrid density functionals with damped atom-atom dispersion corrections. *Physical chemistry chemical physics : PCCP* **2008***,* 10 (44), 6615–6620. https://doi.org/10.1039/b810189b

(12) Weigend, F.; Ahlrichs, R. Balanced basis sets of split valence, triple zeta valence and quadruple zeta valence quality for H to Rn: Design and assessment of accuracy. *Physical chemistry chemical physics : PCCP* **2005**, 7(18), 3297–3305. https://doi.org/10.1039/b508541a

(13) (a) Tomasi, J.; Persico, M. Molecular Interactions in Solution: An Overview of Methods Based on Continuous Distributions of the Solvent. *Chem. Rev.,* **1994**, 94, 2027-2094. https://doi.org/10.1021/cr00031a013 (b) Cossi, M.; Scalmani, G.; Rega, N.; Barone, V. New developments in the polarizable continuum model for quantum mechanical and classical calculations on molecules in solution. *J. Chem. Phys.,* **2002**, 117, 43-54. https://doi.org/10.1063/1.1480445

(14) (a) Schlegel, H. B. Optimization of equilibrium geometries and transition structures. *J. Comput. Chem*. **1982**, 3, 214-218. https://doi.org/10.1002/jcc.540030212 (b) Schlegel, H. B. In Modern Electronic Structure Theory; Yarkony, D. R., Ed.; World Scientific Publishing: Singapore, **1994**

(15) (a) Fukui, K. The path of chemical reactions - the IRC approach. *Acc. Chem. Res.* **1981**, 14, 363-368. https://doi.org/10.1021/ar00072a001 (b) Fukui, K. Formulation of the reaction coordinate. *J. Phys. Chem*. **1970**, 74, 4161-4163. https://doi.org/10.1021/j100717a029

(16) Tanaka, R.; Yamashita, M.; Chung, L. W.; Morokuma, K.; Nozaki, K. Mechanistic Studies on the Reversible Hydrogenation of Carbon Dioxide Catalyzed by an Ir-PNP Complex. *Organometallics* **2011**, 30, 6742-6750. https://doi.org/10.1021/om2010172

(17) (a) Johnson, E. R.; Keinan, S.; Mori-Sánchez, P.; Contreras-García, J.; Cohen, A. J.; Yang, W. Revealing noncovalent interactions. *Journal of the American Chemical Society* **2010**, 132(18), 6498–6506. https://doi.org/10.1021/ja100936w. (b) Lane, J. R.; Contreras-García, J.; Piquemal, J. P.; Miller, B. J.; Kjaergaard, H. G. Are Bond Critical Points Really Critical for Hydrogen Bonding?. *Journal of chemical theory and computation* **2013**, 9(8), 3263–3266. https://doi.org/10.1021/ct400420r

(18) Contreras-García, J.; Johnson, E. R.; Keinan, S.; Chaudret, R.; Piquemal, J. P.; Beratan, D. N.; Yang, W. NCIPLOT: a program for plotting non-covalent interaction regions. *Journal of chemical theory and computation* **2011**, 7(3), 625–632. https://doi.org/10.1021/ct100641a

(19) Humphrey, W.; Dalke, A.; Schulten, K. VMD: visual molecular dynamics. *Journal of molecular graphics* **1996**, 14(1), 33–28. https://doi.org/10.1016/0263-7855(96)00018-5

(20) Legault, C. Y. Université de Sherbrooke, **2009**, http://www.cylview.org

16. NMR spectra

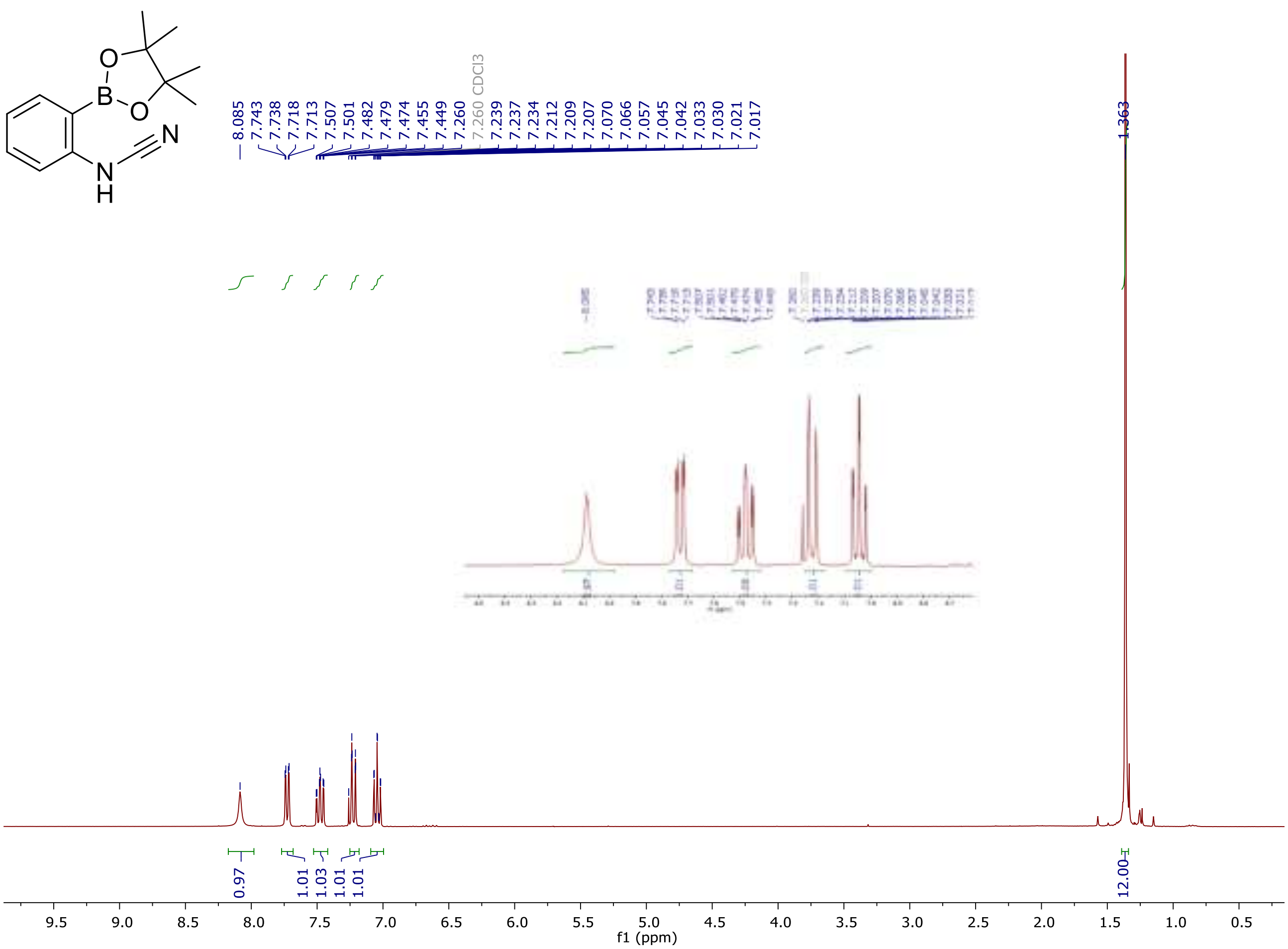

Figure S63. ^{1}H-NMR of cyanamide **1** (300 MHz, $CDCl_3$)

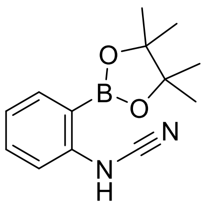

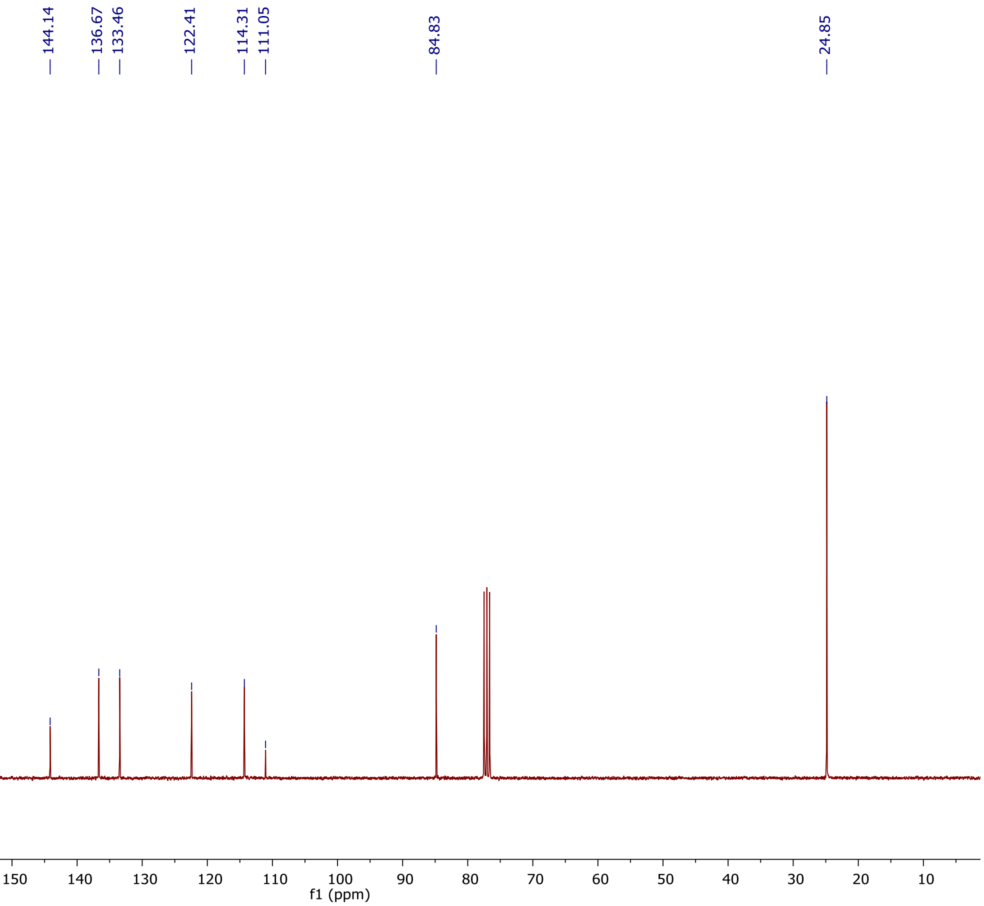

Figure S64. ^{13}C-NMR of cyanamide **1** (75 MHz, $CDCl_3$)

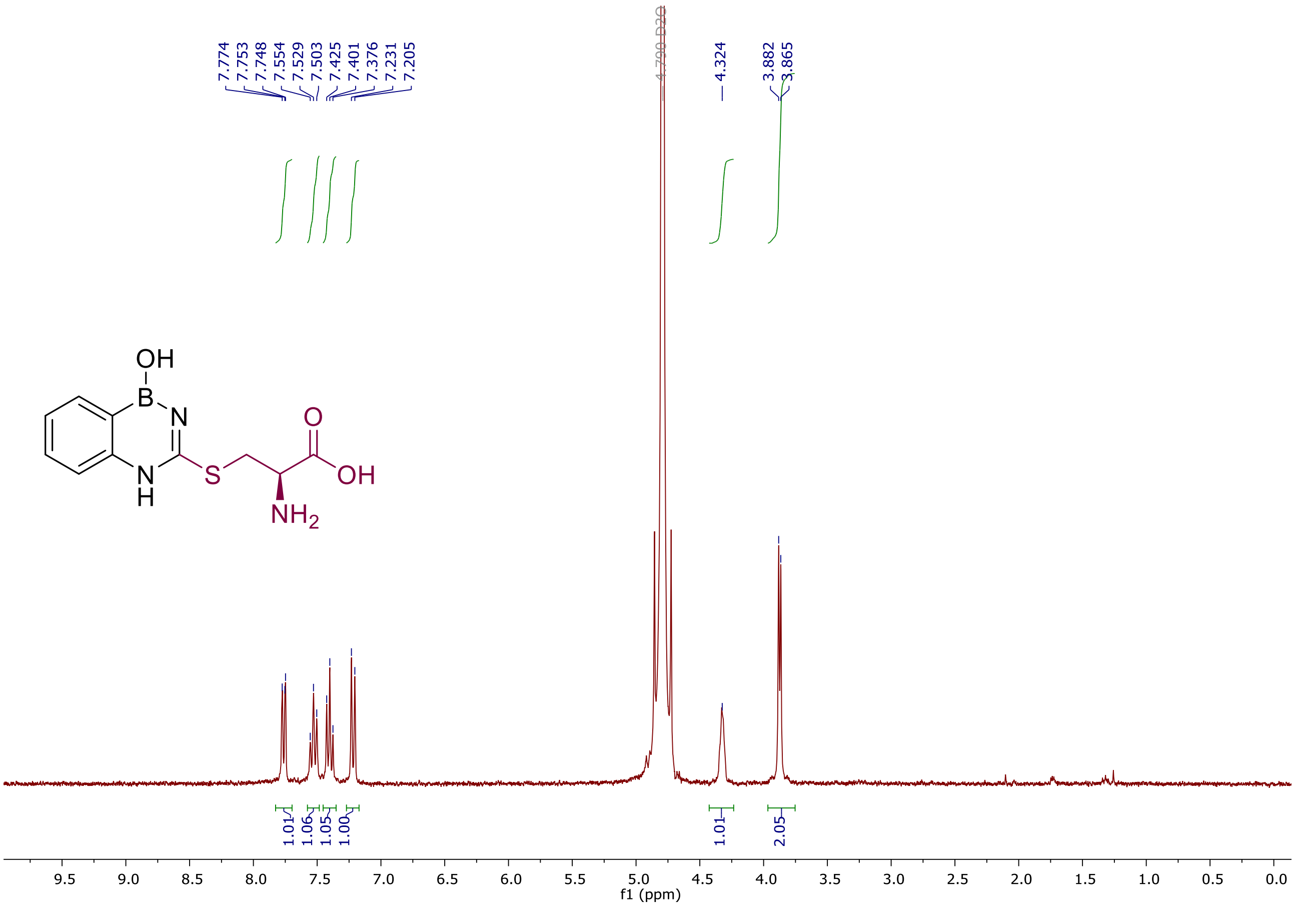

Figure S65. ^{1}H-NMR of BDAB **2** (300 MHz, D_2O)

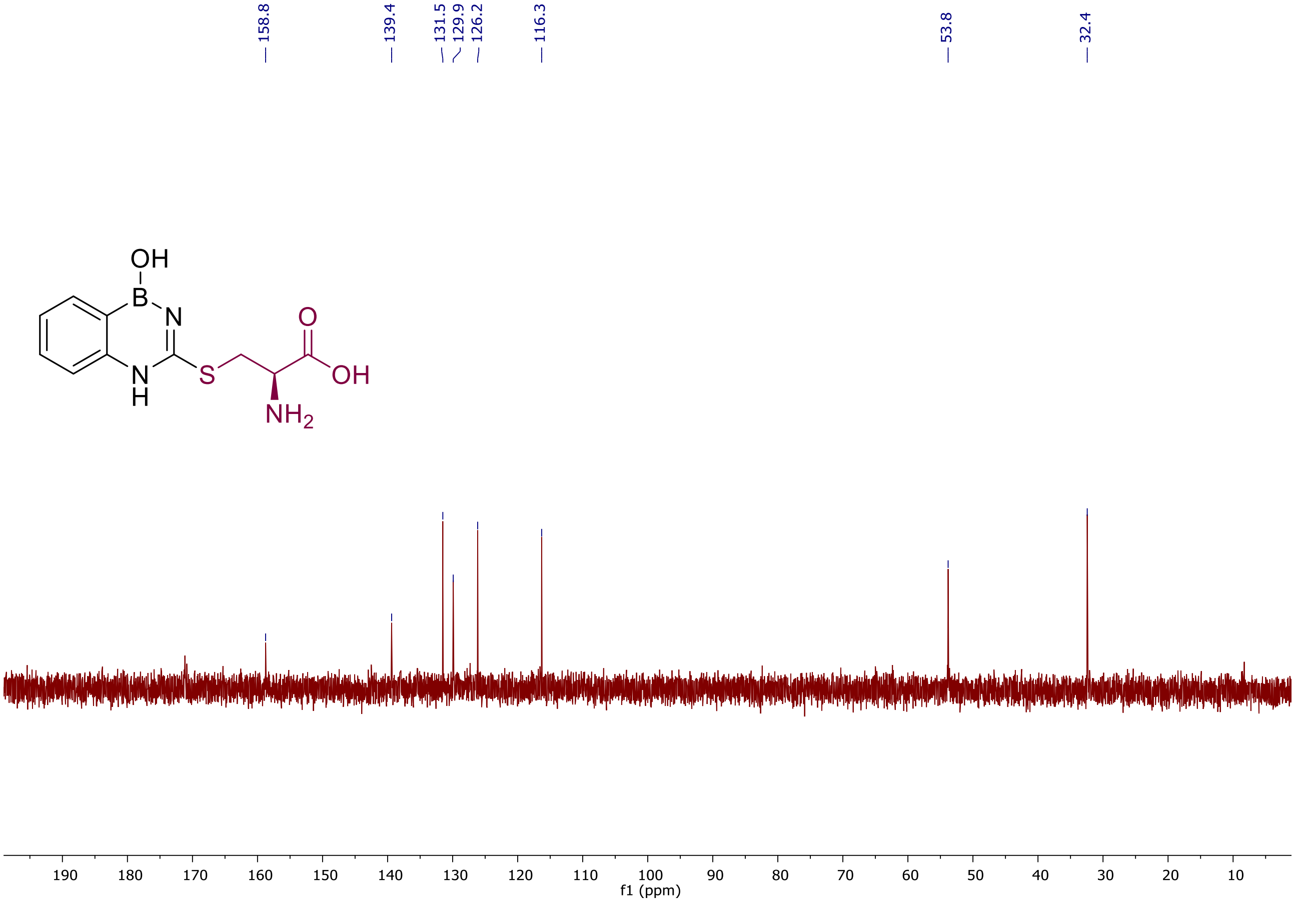

Figure S66. ^{13}C-NMR of BDAB **2** (101 MHz, D_2O)

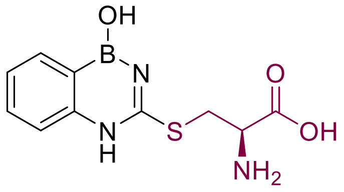

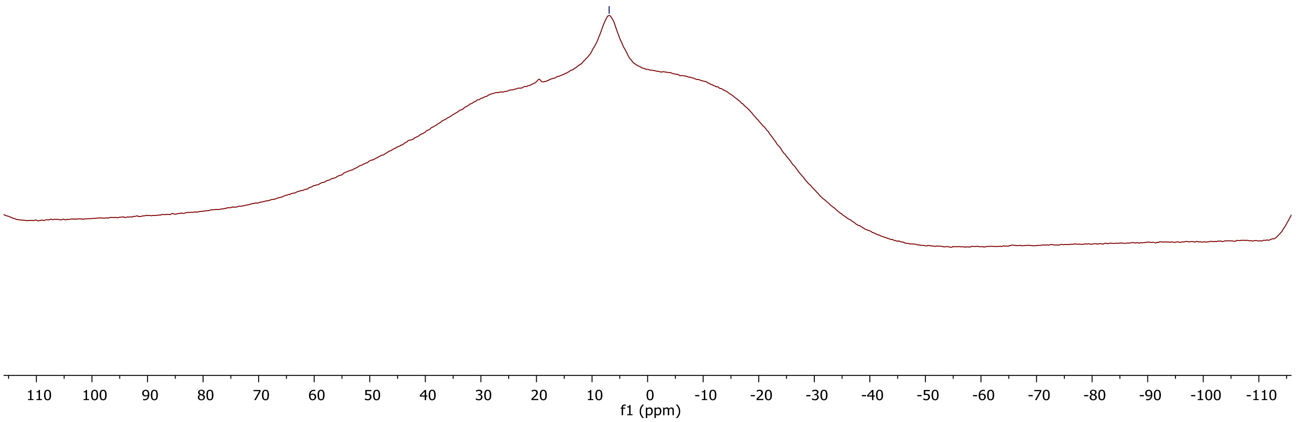

Figure S67. ^{11}B-NMR of BDAB **2** (128 MHz, D_2O)

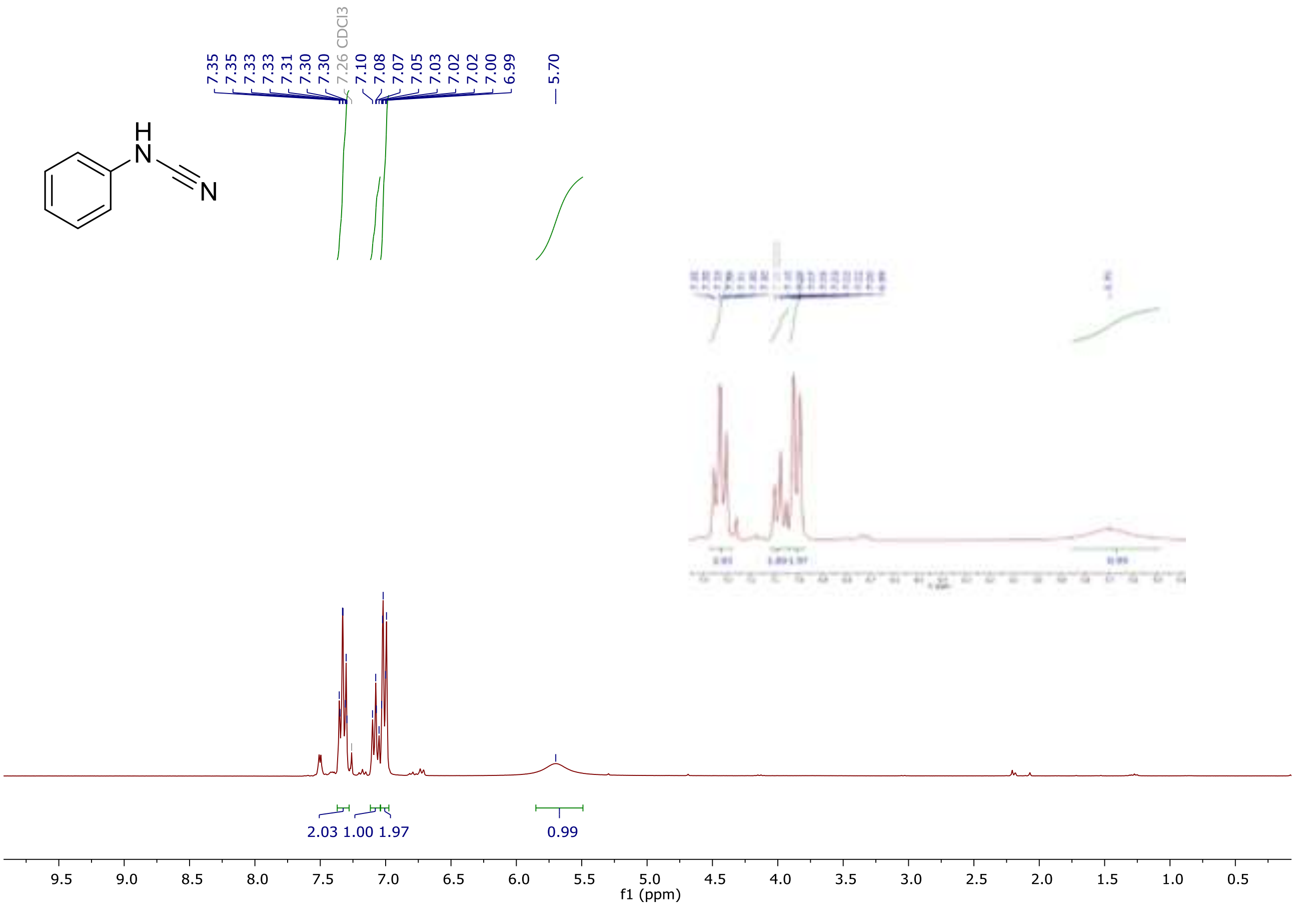

Figure S68. ^{1}H-NMR of cyanamide **3** (300 MHz, $CDCl_3$)

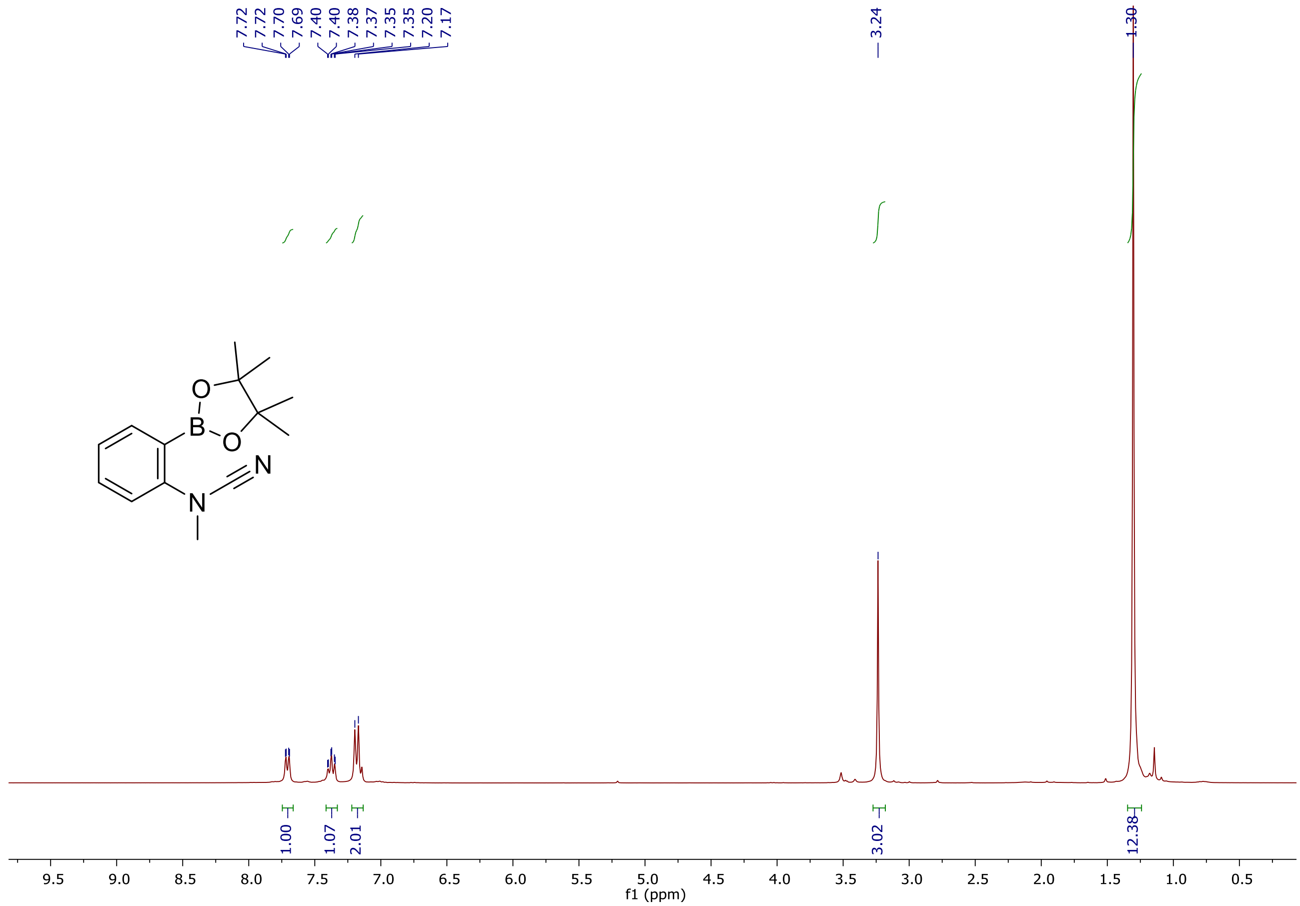

Figure S69. ^{1}H-NMR of cyanamide **6** (300 MHz, $CDCl_3$)

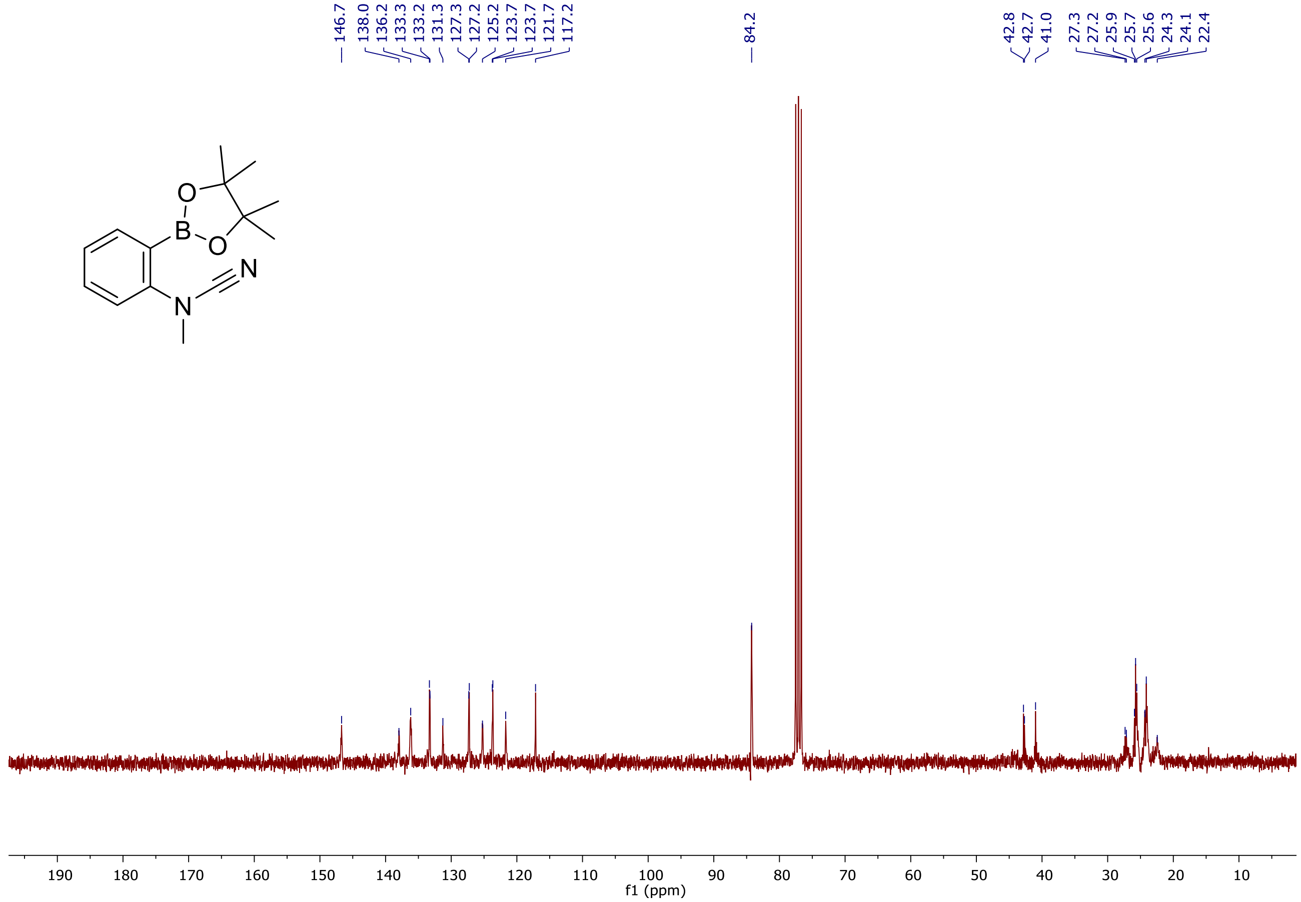

Figure S70. ^{13}C-NMR of cyanamide **6** (75 MHz, $CDCl_3$)

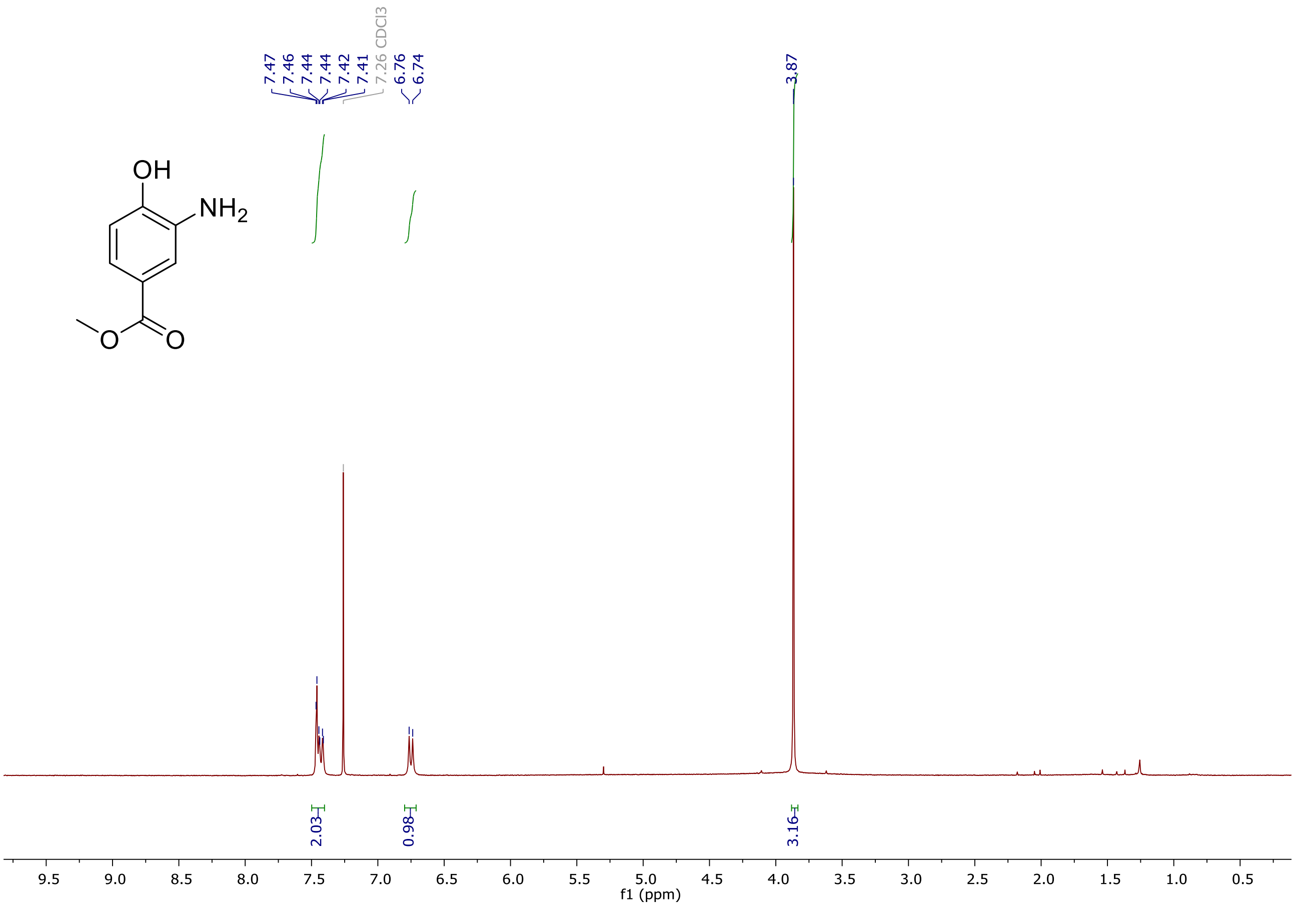

Figure S71. ^{1}H-NMR of compound **S1** (300 MHz, $CDCl_3$)

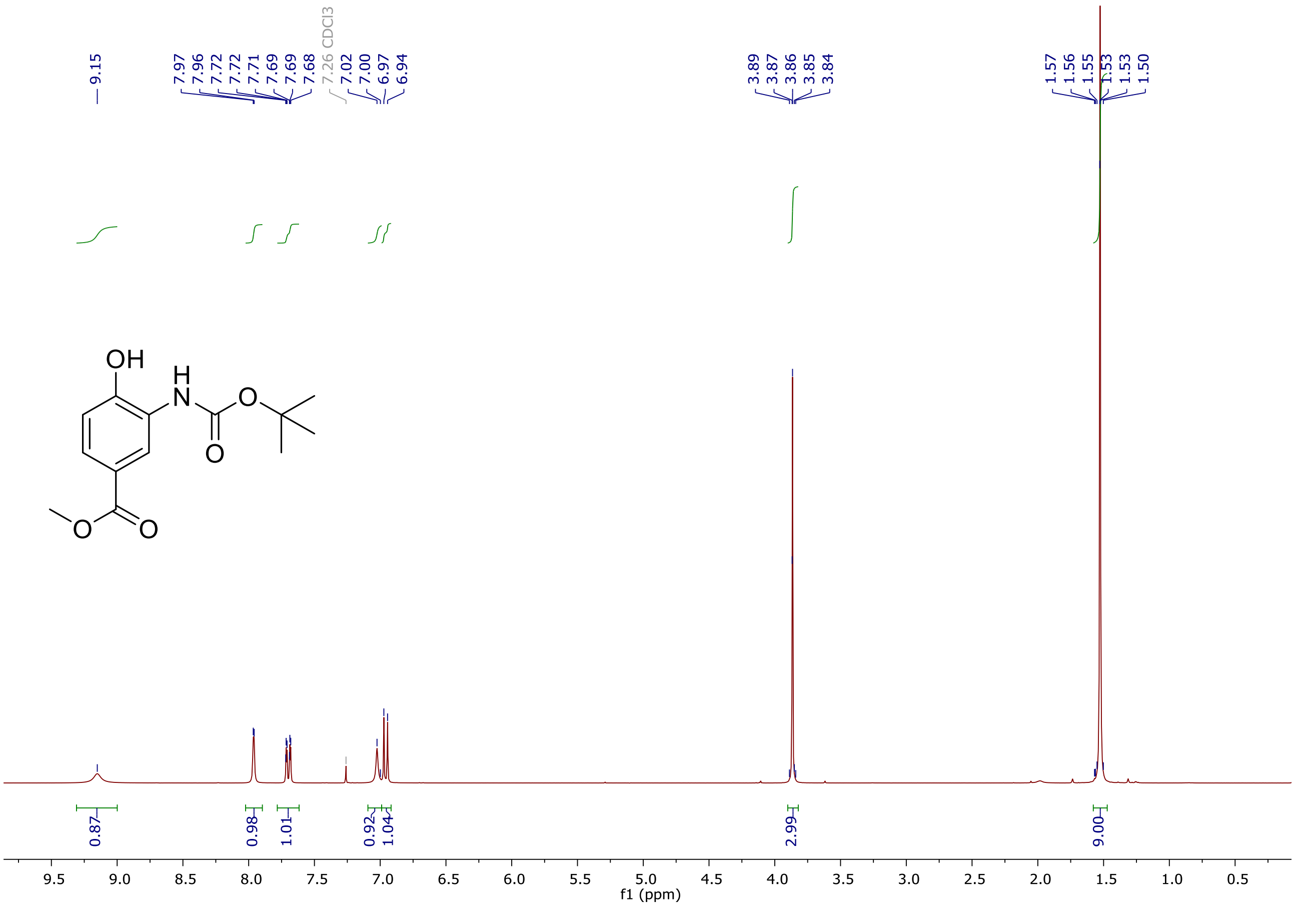

Figure S72. ^{1}H-NMR of compound **S2** (300 MHz, $CDCl_3$)

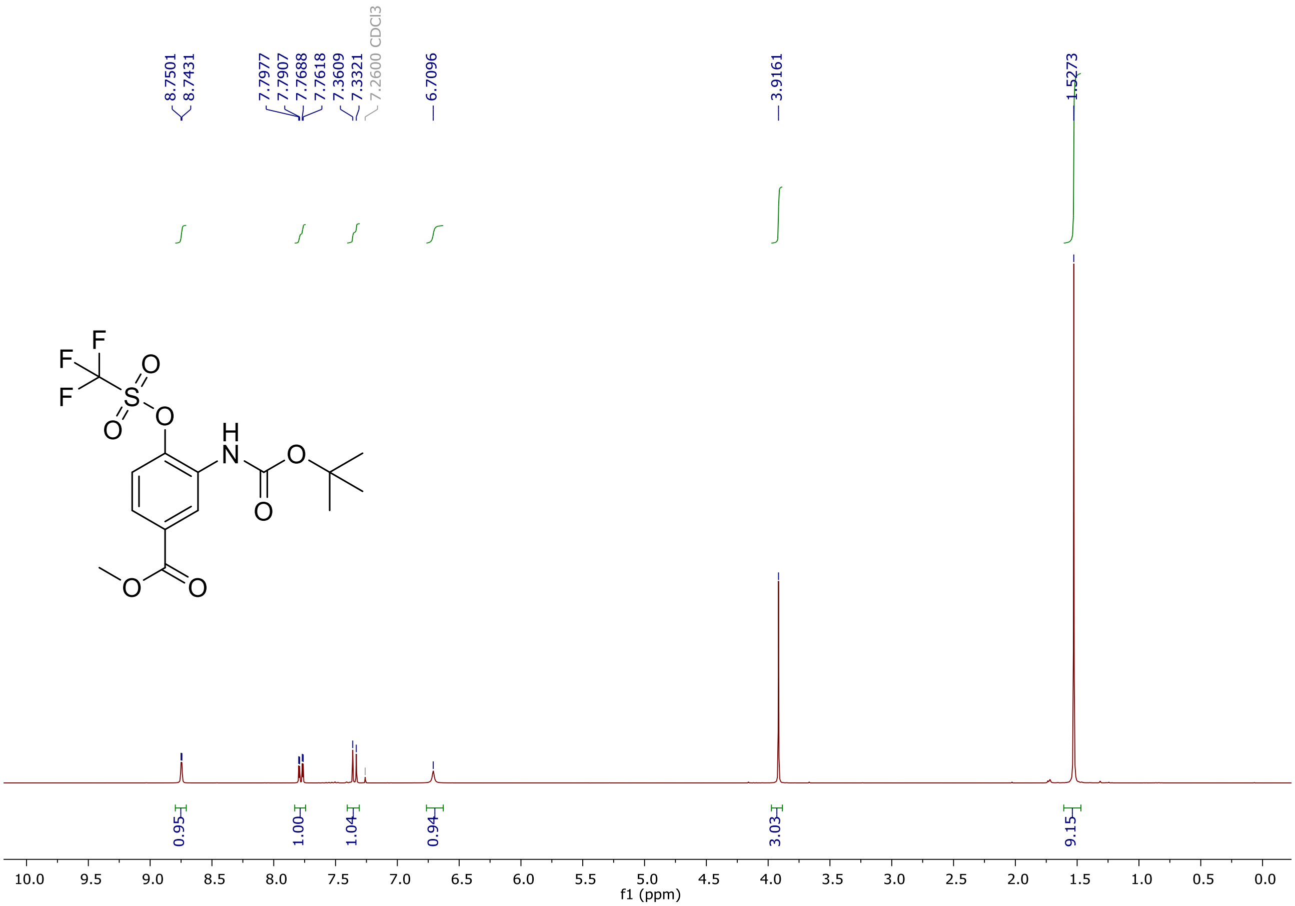

Figure S73. ^{1}H-NMR of compound **S3** (300 MHz, $CDCl_3$)

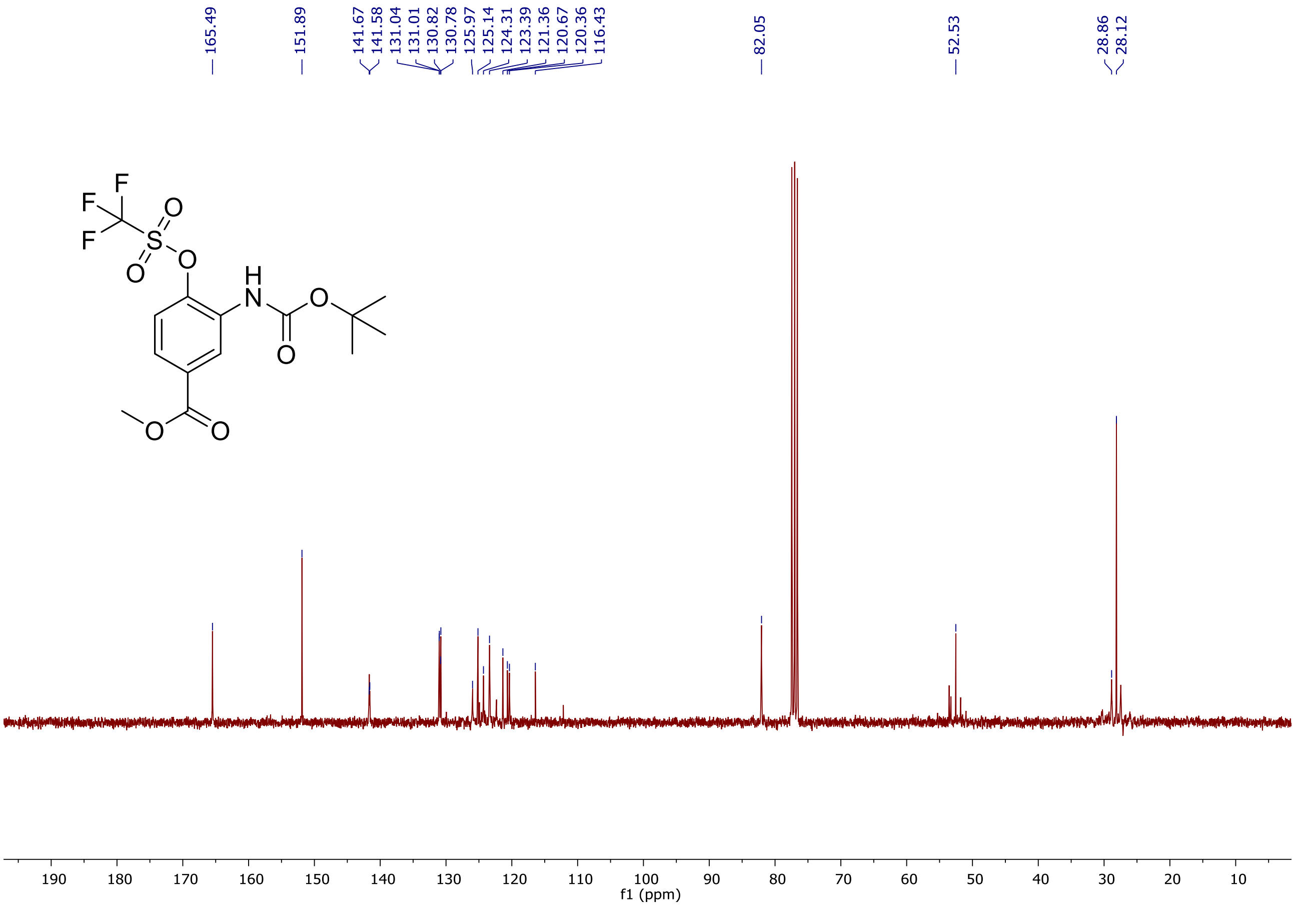

Figure S74. ^{13}C-NMR of compound **S3** (75 MHz, $CDCl_3$)

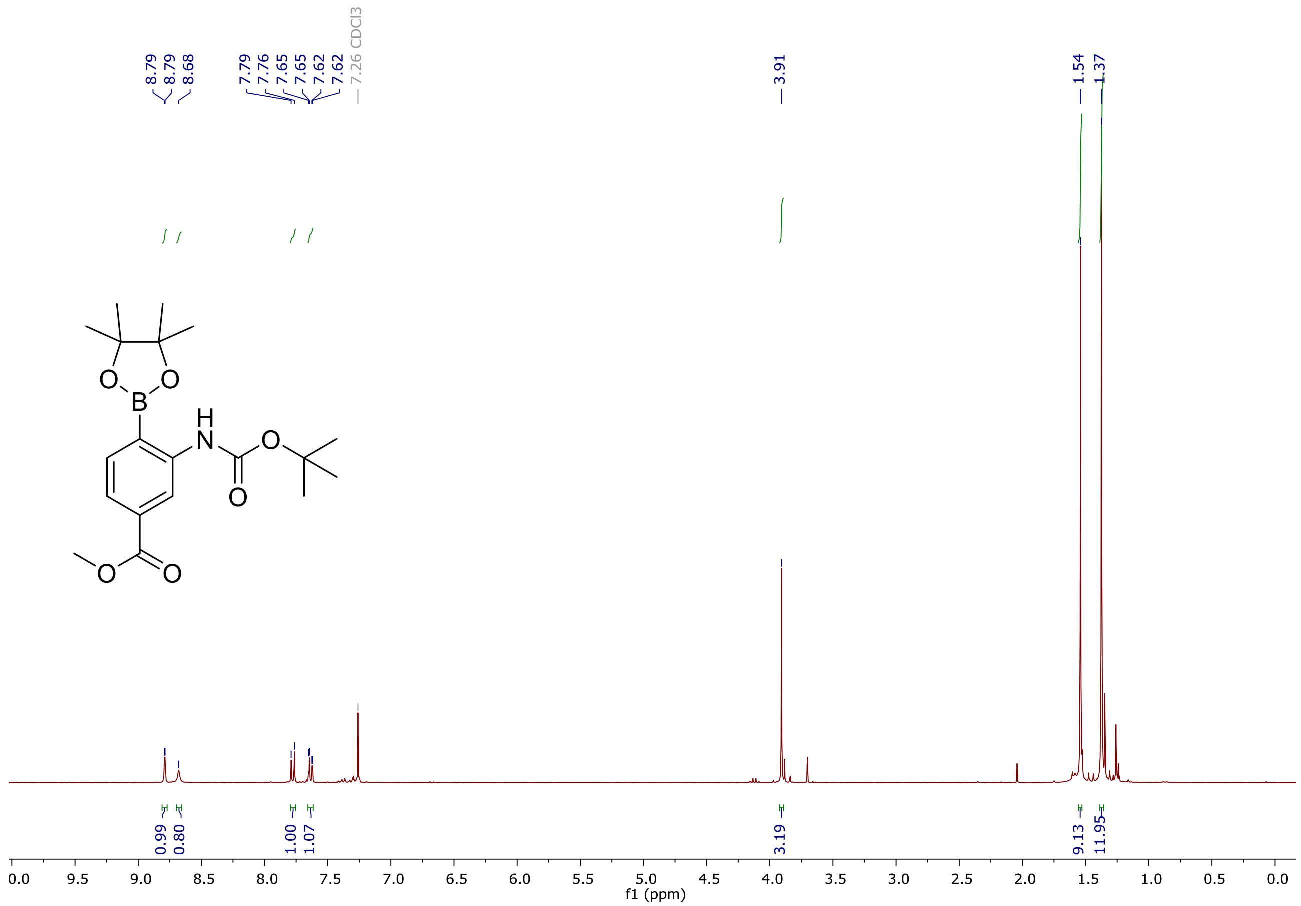

Figure S75. ^{1}H-NMR of compound **S4** (300 MHz, $CDCl_3$)

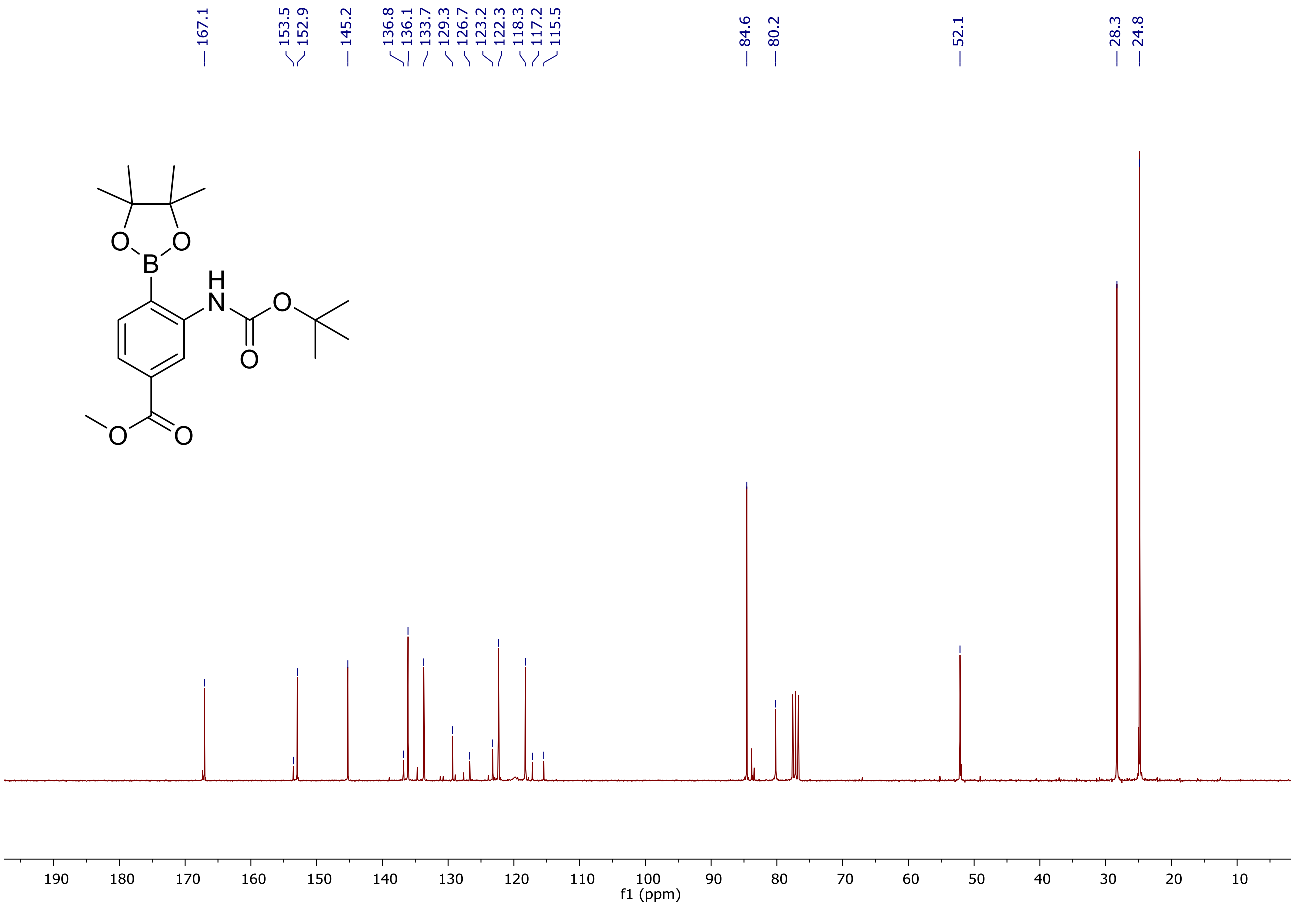

Figure S76. ^{13}C-NMR of compound **S4** (75 MHz, $CDCl_3$)

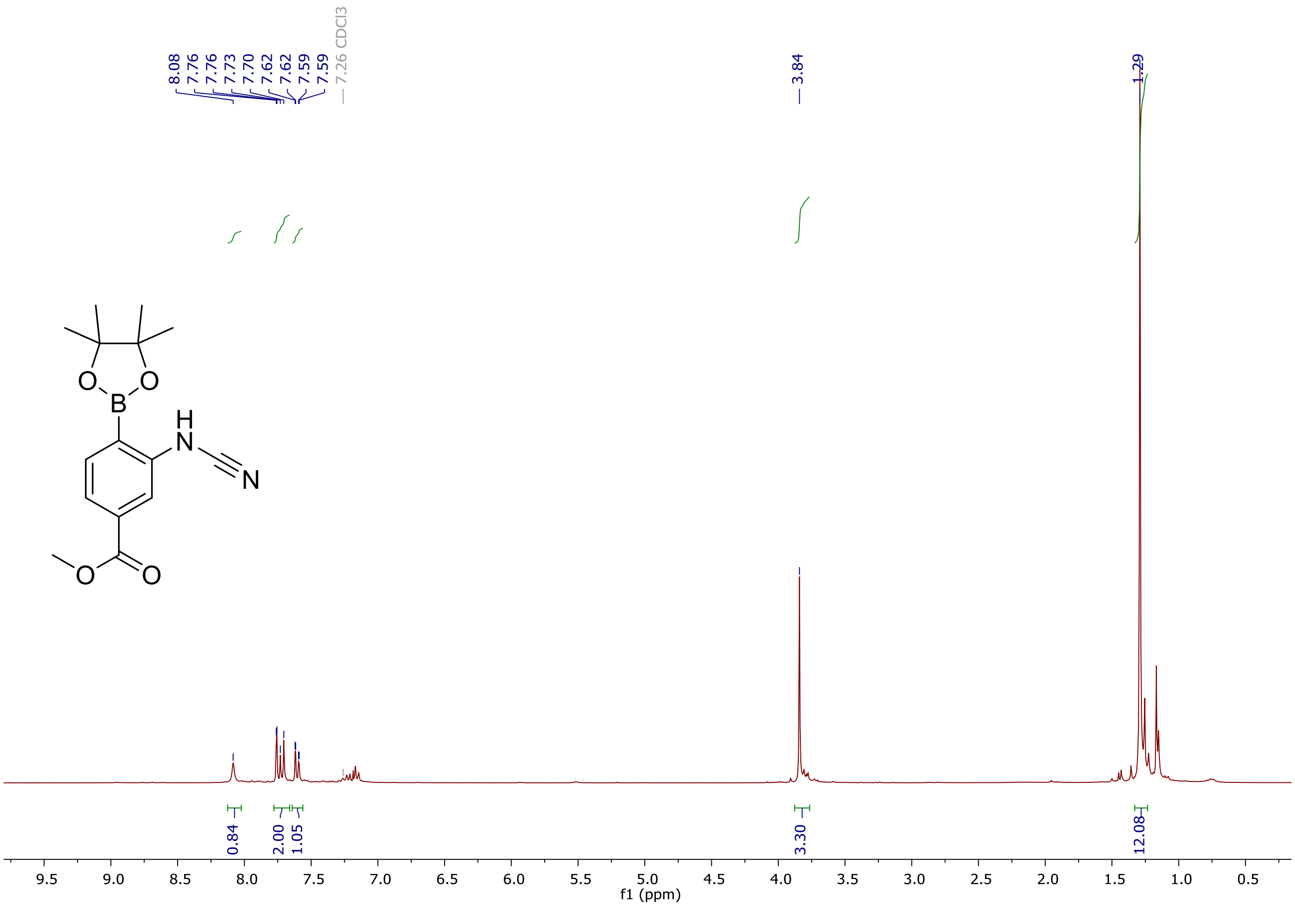

Figure S77. ^{1}H-NMR of cyanamide **8** (300 MHz, $CDCl_3$)

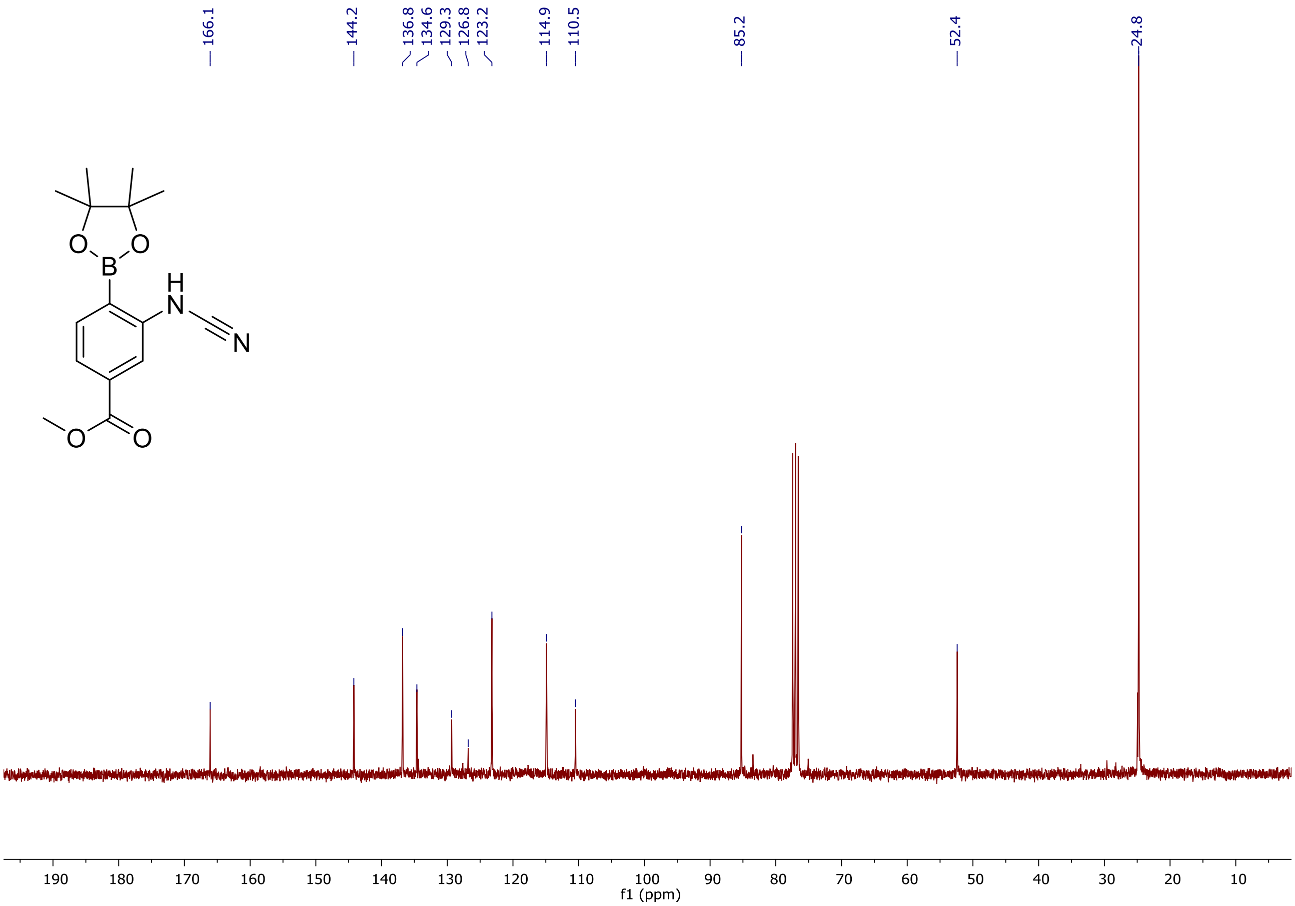

Figure S78. ^{13}C-NMR of cyanamide **8** (75 MHz, $CDCl_3$)
